# Interventions for quitting vaping

**DOI:** 10.1002/14651858.CD016058.pub3

**Published:** 2025-11-25

**Authors:** Ailsa R Butler, Nicola Lindson, Jonathan Livingstone-Banks, Caitlin Notley, Tari Turner, Nancy A Rigotti, Thomas R Fanshawe, Rachna Begh, Angela Difeng Wu, Leonie Brose, Monserrat Conde, Erikas Simonavičius, Jamie Hartmann-Boyce

**Affiliations:** Nuffield Department of Primary Care Health SciencesUniversity of OxfordOxfordUK; Addiction Research Group, Norwich Medical SchoolUniversity of East AngliaNorwichUK; Cochrane AustraliaSchool of Public Health & Preventive MedicineMelbourneAustralia; Tobacco Research and Treatment Center, Department of MedicineMassachusetts General Hospital and Harvard Medical SchoolBostonMassachusettsUSA; Department of Addiction SciencesKing's College LondonLondonUK; Department of Health Promotion and PolicyUniversity of MassachusettsAmherstMAUSA

## Abstract

**Rationale:**

There is limited guidance on how to stop using nicotine‐containing vapes (otherwise known as e‐cigarettes) and ensure long‐term abstinence, whilst minimising the risk of tobacco smoking and other unintended consequences. Treatments could include pharmacological interventions, behavioural interventions, or both.

**Objectives:**

To conduct a living systematic review assessing the benefits and harms of interventions to help people stop vaping compared to each other, placebo or no intervention.

To assess how these interventions affect the use of combustible tobacco, and whether effects vary based on participant characteristics.

**Search methods:**

We searched CENTRAL, MEDLINE, Embase, PsycINFO, ClinicalTrials.gov and WHO International Clinical Trials Registry Platform from 1 January 2004 to 1 July 2025. We also searched the references of eligible studies and abstracts from the Society for Research on Nicotine and Tobacco conferences, and contacted study authors.

**Eligibility criteria:**

We included randomised controlled trials (RCTs) recruiting people of any age using nicotine‐containing vapes, regardless of tobacco smoking status. Studies had to test an intervention designed to support people to quit vaping, and plan to measure at least one of our outcomes.

**Outcomes:**

Critical outcomes: vaping cessation at six months or longer; change in combustible tobacco use at six months or longer; number of participants reporting serious adverse events (SAEs) at one week or longer.

**Risk of bias:**

We used Cochrane's RoB 1 tool to assess risk of bias in the included studies.

**Synthesis methods:**

We followed standard Cochrane methods. We grouped studies by comparisons and outcomes, and calculated individual study and pooled effects, as appropriate. We used random‐effects Mantel‐Haenszel methods to calculate risk ratios (RRs) with 95% confidence intervals (CIs) and random‐effects inverse variance methods to calculate mean differences (MDs) and 95% CIs. We used GRADE to assess the certainty of evidence for our critical outcomes.

**Included studies:**

Fifteen studies (six new to this updated version) involving 5800 participants are included. Fourteen studies included some participants who had previously smoked tobacco; in seven studies, participants were not smoking at baseline. Twelve studies only included participants aged 18 or older (five exclusively included people between 18 and 29); two included some participants under 18 years; and one included 13‐ to 17‐year‐olds only. Fourteen studies were conducted in the USA and one in Italy. We judged five studies at low, six at high, and four at unclear risk of bias.

**Synthesis of results:**

**Pharmacological interventions**

Studies assessed our critical outcomes in relation to combination nicotine replacement therapy (NRT), cytisine, and varenicline compared to placebo or no/minimal support. For combination NRT versus no/minimal support, there was no clear evidence of benefit for vaping cessation rates at six months or longer, with the CI incorporating the possibility of reduced and increased cessation rates (very low‐certainty evidence due to imprecision and risk of bias; RR 0.96, 95% CI 0.73 to 1.25; I² = 0%; 2 studies, 214 participants). One study investigating cytisine versus placebo did not report vaping cessation at six months or longer. When compared to placebo, varenicline increased vaping cessation rates at six months, but evidence was of low certainty due to imprecision (RR 2.71, 95% CI 1.33 to 5.49; I^2^ = 48%; 2 studies, 315 participants).

One study comparing combination NRT versus no/minimal support reported combustible tobacco cessation. There was no clear evidence of higher tobacco cessation in either arm, and CIs were imprecise. The evidence was of very low certainty due to imprecision and risk of bias (RR 0.99, 95% CI 0.71 to 1.37; 198 participants).

Zero participants reported SAEs in the two studies investigating combination NRT versus no/minimal support (706 participants; very low‐certainty evidence due to risk of bias and imprecision) and in the one study investigating cytisine versus placebo (159 participants; low‐certainty evidence due to imprecision). Four studies evaluating varenicline versus placebo measured SAEs, with zero events reported in two studies. Thus, our effect estimate was based on two studies (RR 2.82, 95% CI 0.45 to 17.59; 304 participants; low‐certainty evidence due to imprecision).

**Behavioural interventions**

Studies assessed our critical outcomes in relation to reducing nicotine/vaping behaviour and text message‐based interventions compared to no/minimal support. There was no clear evidence that nicotine/vaping reduction increased vaping cessation at six months compared to minimal support (RR 3.38, 95% CI 0.43 to 26.30; 1 study, 17 participants; very low‐certainty evidence due to imprecision and risk of bias). There was low‐certainty evidence (due to indirectness) that text message‐based interventions may increase vaping cessation rates compared to no/minimal support in 13‐ to 24‐year‐olds specifically (RR 1.32, 95% CI 1.19 to 1.47; I^2^ = 0%; 2 studies, 4091 participants). There was very low‐certainty evidence (due to indirectness and imprecision) that text message‐based interventions for vaping cessation may result in little to no difference in smoking uptake (RR 1.04, 95% CI 0.81 to 1.33; 1 study, 1036 participants) or cessation (RR 1.03, 95% CI 0.90 to 1.19; 1 study, 793 participants) compared to no/minimal support.

The one study investigating nicotine/vaping behaviour reduction versus minimal support did not report SAEs. Three studies investigating text message‐based interventions reported SAEs; however, zero events were reported (2082 participants; low‐certainty evidence due to imprecision).

**Authors' conclusions:**

Low‐certainty evidence suggests that text message‐based interventions to help people stop nicotine vaping may help more youths and young adults to successfully stop compared to no/minimal support, with very uncertain evidence regarding their effect on smoking behaviours. Low‐certainty evidence suggests that varenicline may help people quit vaping. Data exploring the effectiveness of combination NRT, cytisine, and nicotine/vaping behaviour reduction are inconclusive due to risk of bias and imprecision.

Most studies that measured SAEs reported that none had occurred; however, more data are needed to draw clear conclusions. Studies that have investigated these interventions for quitting smoking have not demonstrated serious concerns about SAEs. It is important that future studies measure combustible tobacco outcomes so the complete risk profile of relevant interventions can be considered.

Further RCTs are underway. To ensure this review continues to provide up‐to‐date information to decision‐makers, we will maintain it as a living systematic review by running searches monthly and updating the review when relevant new evidence that will strengthen or change our conclusions emerges.

**Funding:**

Cancer Research UK (PICCTR‐2024/100012); National Institute for Health and Care Research (NIHR206123).

**Registration:**

Protocol (2024) DOI: 10.1002/14651858.CD016058

Original review (2025) DOI: 10.1002/14651858.CD016058.pub2

## Plain language summary

**What are the effects of treatments to help people stop vaping nicotine?**

**Key messages**

- Using text message‐based interventions may help more young people stop vaping compared to no or minimal support.
- Varenicline may help more people stop vaping compared to placebo (dummy treatment).
- We need more information on whether these and other interventions can help people stop vaping, their potential harms and whether they affect the number of people smoking tobacco.

**What are nicotine‐containing vapes?**

Vapes (also known as electronic cigarettes or e‐cigarettes) are handheld devices that heat liquid usually containing nicotine and flavourings. Liquids are stored in a disposable or refillable cartridge, reservoir, or 'pod'. Vapes allow users to inhale nicotine as vapour rather than smoke. Because they do not burn tobacco, vapes expose users to fewer harmful substances than tobacco cigarettes. However, vaping is likely to cause more harm than not vaping. Some people use nicotine vapes to stop smoking; however, some people who vape nicotine have never smoked. People may want to stop using nicotine vapes, but find it difficult due to the addictive properties of nicotine.

**What interventions might help people to stop vaping?**

Medicines like nicotine replacement therapy (NRT; such as gums, patches), varenicline, bupropion, and cytisine, which help people to stop smoking, could also be used to stop vaping. Behavioural interventions to help people stop vaping could include counselling, text messages, vaping reduction programmes and printed materials.

**What did we want to find out?**

There is limited guidance on the best ways to stop nicotine vaping. We wanted to know what tools have been tested and whether they can help people stop vaping. We also looked at the potential harms of these interventions, including whether they could cause more people to smoke tobacco.

**What did we do?**

We searched for studies looking at any intervention designed to support people using nicotine‐containing vapes to stop vaping. We compared and summarised our results and rated our confidence in the evidence based on study methods and sizes.

We looked at:

- how many people stopped vaping at least six months after study start;
- changes in tobacco smoking at least six months after study start;
- how many people experienced unwanted effects at least one week after treatment started.

**What did we find?**

We found 15 studies, including 5800 people who used nicotine vapes. Fourteen studies took place in the USA and one in Italy. Treatments used to help people stop vaping were:

- two types of NRT used together (combination NRT);
- different doses of NRT;
- cytisine (a stop‐smoking medicine);
- varenicline (a stop‐smoking medicine);
- a plan to help people reduce the nicotine in their vapes and the amount they were vaping;
- text messaging with or without apps;
- media literacy e‐learning;
- financial incentives.

**Main results**

Text message support may help more young people (13 to 24 years of age) stop vaping than no/minimal support. Varenicline may also help people quit vaping. There was not enough information to say whether other interventions could help people stop vaping.

Very few studies reported any serious unwanted effects, but there was not enough information to draw any conclusions about potential harms of the treatments. Two studies looked at whether an intervention (combination NRT; text messaging) affected the number of people smoking tobacco after six months; the evidence suggests these interventions did not affect smoking, but we are very uncertain about the results.

**What are the limitations of the evidence?**

We found some evidence that young people may be more likely to stop vaping nicotine using a text messaging intervention than using no or minimal support. However, the studies testing this used the same text message intervention, meaning we do not know whether we would find similar results for other texting interventions or in older adults. We also found that varenicline may help more people to stop vaping nicotine than placebo (dummy treatment), but further evidence could change our conclusions.

We were unable to draw any clear conclusions regarding the number of people who quit tobacco smoking at six months or serious unwanted effects of the interventions under study. More studies will help us feel more confident.

Overall, the findings are based on small numbers of studies and participants, and in some cases there were problems with study methods, limiting our confidence in the evidence. More studies are underway, which will improve our confidence in the evidence.

**How up‐to‐date is this evidence?**

This review updates our previous review. The evidence is current to 1 July 2025. This is a living systematic review. We search for new evidence every month and update the review when we identify new evidence that strengthens or changes our conclusions.

## Summary of findings

**Summary of findings 1:** Summary of findings table ‐ Combination nicotine replacement therapy compared to no/minimal support for nicotine vaping cessation

**Combination nicotine replacement therapy compared to no/minimal support for nicotine vaping cessation**						
**Patient or population:** nicotine vaping cessation **Setting:** any (USA) **Intervention:** combination nicotine replacement therapy **Comparison:** no/minimal support						
Outcomes	Anticipated absolute effects^*^ (95% CI)		Relative effect (95% CI)	№ of participants (studies)	Certainty of the evidence (GRADE)	Comments
	Risk with no/minimal support	Risk with combination nicotine replacement therapy				
Vaping cessation at 6 months or longer	50 per 100	**48 per 100** (36 to 62)	**RR 0.96** (0.73 to 1.25)	214 (2 RCTs)	⊕⊝⊝⊝ Very low^a,^^b^	
Combustible tobacco cessation at 6 months or longer	43 per 100	**42 per 100** (30 to 59)	**RR 0.99** (0.71 to 1.37)	198 (1 RCT)	⊕⊝⊝⊝ Very low^b,^^c^	
Number of participants reporting serious adverse events	Not pooled	Not pooled	Not pooled	706 (2 RCTs)	⊕⊝⊝⊝ Very low^a,^^d^	We did not calculate relative or absolute effects as there were no events across study arms in either study.
*The risk in the intervention group (and its 95% confidence interval) is based on the assumed risk in the comparison group and the **relative effect** of the intervention (and its 95% CI). **CI:** confidence interval; **RR:** risk ratio						
**GRADE Working Group grades of evidence** **High certainty:** we are very confident that the true effect lies close to that of the estimate of the effect. **Moderate certainty:** we are moderately confident in the effect estimate: the true effect is likely to be close to the estimate of the effect, but there is a possibility that it is substantially different. **Low certainty:** our confidence in the effect estimate is limited: the true effect may be substantially different from the estimate of the effect. **Very low certainty:** we have very little confidence in the effect estimate: the true effect is likely to be substantially different from the estimate of effect.						
See interactive version of this table: https://gdt.gradepro.org/presentations/#/isof/isof_question_revman_web_451184852882794857.						

^a^ Downgraded two levels due to risk of bias: both studies contributing were judged to be at high risk of bias. ^b^ Downgraded two levels due to imprecision: 95% CI incorporates the potential for no effect, plus both a potential benefit and harm of the intervention. ^c^ Downgraded two levels due to risk of bias: the one study contributing was judged to be at high risk of bias. ^d^ Downgraded two levels due to imprecision: no events recorded across study arms.

**Summary of findings 2:** Summary of findings table ‐ Cytisine compared to placebo for nicotine vaping cessation

**Cytisine compared to placebo for nicotine vaping cessation**						
**Patient or population:** nicotine vaping cessation **Setting:** any (USA) **Intervention:** cytisine **Comparison:** placebo						
Outcomes	Anticipated absolute effects^*^ (95% CI)		Relative effect (95% CI)	№ of participants (studies)	Certainty of the evidence (GRADE)	Comments
	Risk with placebo	Risk with cytisine				
Vaping cessation at 6 months or longer ‐ not reported	‐	‐	‐	‐	‐	No studies reported this outcome.
Change in combustible tobacco use at 6 months or longer ‐ not reported	‐	‐	‐	‐	‐	No studies reported this outcome.
Number of participants reporting serious adverse events	Not pooled	Not pooled	Not pooled	159 (1 RCT)	⊕⊕⊝⊝ Low^a^	We did not calculate relative or absolute effects as there were no events across study arms.
*The risk in the intervention group (and its 95% confidence interval) is based on the assumed risk in the comparison group and the **relative effect** of the intervention (and its 95% CI). **CI:** confidence interval						
**GRADE Working Group grades of evidence** **High certainty:** we are very confident that the true effect lies close to that of the estimate of the effect. **Moderate certainty:** we are moderately confident in the effect estimate: the true effect is likely to be close to the estimate of the effect, but there is a possibility that it is substantially different. **Low certainty:** our confidence in the effect estimate is limited: the true effect may be substantially different from the estimate of the effect. **Very low certainty:** we have very little confidence in the effect estimate: the true effect is likely to be substantially different from the estimate of effect.						
See interactive version of this table: https://gdt.gradepro.org/presentations/#/isof/isof_question_revman_web_451185324505541376.						

^a^ Downgraded two levels due to imprecision. No events were reported across study arms.

**Summary of findings 3:** Summary of findings table ‐ Varenicline compared to placebo for nicotine vaping cessation

**Varenicline compared to placebo for nicotine vaping cessation**						
**Patient or population:** nicotine vaping cessation **Setting:** any (Italy and USA) **Intervention:** varenicline **Comparison:** placebo						
Outcomes	Anticipated absolute effects^*^ (95% CI)		Relative effect (95% CI)	№ of participants (studies)	Certainty of the evidence (GRADE)	Comments
	Risk with placebo	Risk with varenicline				
Vaping cessation at 6 months or longer ‐ total	11 per 100	**31 per 100** (15 to 63)	**RR 2.71** (1.33 to 5.49)	315 (2 RCTs)	⊕⊕⊝⊝ Low^a^	
Change in combustible tobacco use at 6 months or longer ‐ not reported	‐	‐	‐	‐	‐	No studies reported this outcome.
Number of participants reporting serious adverse events	1 per 100	**2 per 100** (0 to 12)	**RR 2.82** (0.45 to 17.59)	304 (4 RCTs)	⊕⊕⊝⊝ Low^b^	Two of the four studies in this comparison reporting serious adverse events reported zero events in both arms and so only two studies with 304 participants contributed to the effect estimate.
*The risk in the intervention group (and its 95% confidence interval) is based on the assumed risk in the comparison group and the **relative effect** of the intervention (and its 95% CI). **CI:** confidence interval; **RR:** risk ratio						
**GRADE Working Group grades of evidence** **High certainty:** we are very confident that the true effect lies close to that of the estimate of the effect. **Moderate certainty:** we are moderately confident in the effect estimate: the true effect is likely to be close to the estimate of the effect, but there is a possibility that it is substantially different. **Low certainty:** our confidence in the effect estimate is limited: the true effect may be substantially different from the estimate of the effect. **Very low certainty:** we have very little confidence in the effect estimate: the true effect is likely to be substantially different from the estimate of effect.						
See interactive version of this table: https://gdt.gradepro.org/presentations/#/isof/isof_question_revman_web_451184323175229067.						

^a^ Downgraded two levels due to imprecision: small number of events (n = 67) reported across study arms. ^b^ Downgraded two levels due to imprecision: very few events (n = 5) and 95% CI incorporates the potential for benefit, harm, and no effect of the intervention.

**Summary of findings 4:** Summary of findings table ‐ Nicotine/vaping reduction compared to no/minimal support for nicotine vaping cessation

**Nicotine/vaping reduction compared to no/minimal support for nicotine vaping cessation**						
**Patient or population:** nicotine vaping cessation **Setting:** university (USA) **Intervention:** nicotine/vaping reduction **Comparison:** no/minimal support						
Outcomes	Anticipated absolute effects^*^ (95% CI)		Relative effect (95% CI)	№ of participants (studies)	Certainty of the evidence (GRADE)	Comments
	Risk with no/minimal support	Risk with nicotine/vaping reduction				
Vaping cessation at 6 months or longer follow‐up: 6 months	11 per 100	**38 per 100** (5 to 100)	**RR 3.38** (0.43 to 26.30)	17 (1 RCT)	⊕⊝⊝⊝ Very low^a,^^b^	
Change in combustible tobacco use at 6 months or longer ‐ not reported	‐	‐	‐	‐	‐	No studies reported this outcome.
Number of participants reporting serious adverse events ‐ not reported	‐	‐	‐	‐	‐	No studies reported this outcome.
*The risk in the intervention group (and its 95% confidence interval) is based on the assumed risk in the comparison group and the **relative effect** of the intervention (and its 95% CI). **CI:** confidence interval; **RR:** risk ratio						
**GRADE Working Group grades of evidence** **High certainty:** we are very confident that the true effect lies close to that of the estimate of the effect. **Moderate certainty:** we are moderately confident in the effect estimate: the true effect is likely to be close to the estimate of the effect, but there is a possibility that it is substantially different. **Low certainty:** our confidence in the effect estimate is limited: the true effect may be substantially different from the estimate of the effect. **Very low certainty:** we have very little confidence in the effect estimate: the true effect is likely to be substantially different from the estimate of effect.						
See interactive version of this table: https://gdt.gradepro.org/presentations/#/isof/isof_question_revman_web_451185156225608261.						

^a^ Downgraded two levels due to risk of bias: the only study contributing to the comparison and outcome was judged to be at high risk of bias. ^b^ Downgraded two levels due to imprecision: extremely low number of events across study arms and 95% CI encompasses the potential for benefit, harm, and no effect of the intervention.

**Summary of findings 5:** Summary of findings table ‐ Text message‐based interventions compared to no/minimal support for nicotine vaping cessation

**Text message‐based interventions compared to no/minimal support for nicotine vaping cessation**						
**Patient or population:** nicotine vaping cessation **Setting:** any (USA) **Intervention:** text message‐based interventions **Comparison:** no/minimal support						
Outcomes	Anticipated absolute effects^*^ (95% CI)		Relative effect (95% CI)	№ of participants (studies)	Certainty of the evidence (GRADE)	Comments
	Risk with no/minimal support	Risk with text message‐based interventions				
Vaping cessation at 6 months or longer	22 per 100	**29 per 100** (26 to 32)	**RR 1.32** (1.19 to 1.47)	4091 (2 RCTs)	⊕⊕⊝⊝ Low^a^	
Combustible tobacco abstinence at 6 months or longer among people who smoked at baseline	49 per 100	**50 per 100** (44 to 58)	**RR 1.03** (0.90 to 1.19)	793 (1 RCT)	⊕⊝⊝⊝ Very low^a,^^b^	
Combustible tobacco use uptake at 6 months or longer among people who did not smoke at baseline	19 per 100	**20 per 100** (16 to 26)	**RR 1.04** (0.81 to 1.33)	1036 (1 RCT)	⊕⊝⊝⊝ Very low^a,^^b^	
Number of participants reporting serious adverse events	Not pooled	Not pooled	Not pooled	2082 (3 RCTs)	⊕⊕⊝⊝ Low^c^	We did not calculate relative or absolute effects as there were no events across relevant studies and study arms.
*The risk in the intervention group (and its 95% confidence interval) is based on the assumed risk in the comparison group and the **relative effect** of the intervention (and its 95% CI). **CI:** confidence interval; **RR:** risk ratio						
**GRADE Working Group grades of evidence** **High certainty:** we are very confident that the true effect lies close to that of the estimate of the effect. **Moderate certainty:** we are moderately confident in the effect estimate: the true effect is likely to be close to the estimate of the effect, but there is a possibility that it is substantially different. **Low certainty:** our confidence in the effect estimate is limited: the true effect may be substantially different from the estimate of the effect. **Very low certainty:** we have very little confidence in the effect estimate: the true effect is likely to be substantially different from the estimate of effect.						
See interactive version of this table: https://gdt.gradepro.org/presentations/#/isof/isof_question_revman_web_451185315991890681.						

^a^ Downgraded two levels due to indirectness: the contributing studies tested the same intervention in a relatively homogenous population. Unclear if the effects can be generalised to other text message‐based interventions and other populations. ^b^ Downgraded two levels due to imprecision: the CIs demonstrate evidence of clinically significant benefit and clinically significant harm. ^c^ Downgraded two levels due to imprecision. No events were recorded across study arms.

## Background

### Description of the condition

Vapes or electronic cigarettes are handheld electronic devices that produce an aerosol by heating an e‐liquid [1]. The e‐liquid, usually comprising propylene glycol (a synthetic liquid substance that absorbs water) and/or glycerol (a naturally occurring alcohol), with or without nicotine and flavours, is stored in disposable or refillable cartridges or a reservoir or 'pod' [2]. Evidence suggests that nicotine‐containing vapes or electronic cigarettes are less harmful to health than tobacco cigarettes, and in some countries they are endorsed as smoking cessation aids [3, 4, 5, 6]. However, there are concerns about their potential harm to health if used long‐term by people who stopped smoking a long time ago or by people who have never smoked, with particular concerns relating to young people [7, 8, 9, 10]. Young people who take up nicotine vaping who have never smoked may develop a dependence on nicotine, which has led to concerns that they may be more likely to try other more harmful nicotine‐containing products, such as combustible tobacco cigarettes [11, 12, 13]. We do not yet have any evidence on the long‐term harms of nicotine vaping in the absence of a tobacco smoking history; therefore, potential health harms of vaping itself are an additional, as yet unquantified, concern. Even if modest in comparison to smoking tobacco, it is unlikely that vaping nicotine will be completely risk or harm free. Consequently, there are clear reasons to support people to stop nicotine vaping. There are also various reasons that people (of any age) who have used vapes for tobacco smoking cessation may ultimately want to stop using them. Commonly cited reasons include cost, concerns around health, perceptions of friends and family, concerns about dependence on nicotine, and stigma [14]. However, as nicotine is an addictive substance, and advancements in vaping technology make it increasingly effective at delivering nicotine to the brain, it may not be easy for people to discontinue use. In addition, people who have used vapes to stop smoking need to ensure that they are no longer at risk of relapsing to smoking if they stop using vapes. Where nicotine vaping is supported as a smoking cessation aid, there is a growing awareness that support to stop using vapes may be needed once people have fully stopped smoking.

There is currently a paucity of evidence on the best methods to stop using vapes. However, more research is emerging, with a number of ongoing studies currently registered. More relevant evidence is likely to emerge in the near future, making a living systematic review approach appropriate. Our living review approach is well suited to collating and assessing the evidence from new and ongoing studies as this information emerges.

### Description of the intervention and how it might work

Treatments to support people to stop vaping may include both pharmacological and behavioural interventions. Potential pharmacological treatments include nicotine replacement therapy (gums, patches, lozenges, etc., which can be used alone or in combination), varenicline, bupropion, and cytisine, which are already used as aids to support people to stop smoking. Behavioural stop‐smoking interventions may also be adopted or adapted to support people to stop vaping, such as in‐person or telephone‐based counselling (one‐to‐one or group‐based), print‐based support and/or incentives to stop. Alternative therapies such as hypnotherapy or acupuncture may also be tested. Additionally, intervention delivery approaches, such as text message support, smartphone apps, or online support tools, could be used. In the UK, National Institute for Health and Care Excellence (NICE) guidance recommends that the National Health Service (NHS) provide support to help people who vape to stop when they are ready to do so, but does not set out how best to achieve this [15].

When used for smoking cessation, nicotine replacement strategies offer an approach based on harm reduction principles, substituting the nicotine consumed through smoking with nicotine delivered in other forms (e.g. transdermally or across oral mucous membranes). This in turn alleviates symptoms of nicotine withdrawal and breaks associations between nicotine delivery and reward. Cytisine and varenicline (nicotine receptor partial agonists) work by blocking some of the receptors in the brain associated with nicotine addiction, thereby also reducing the rewarding effects of smoking cigarettes containing nicotine [16]. Bupropion increases dopamine release in the brain's mesolimbic pathways that are stimulated by other addictive substances [17]. It is unclear exactly how this impacts mechanisms of nicotine addiction. These interventions have been well tested in smoking cessation trials, with evidence of effectiveness for smoking cessation [18]. Their use for vaping cessation is in its infancy, and no clear conclusions have been drawn on their effectiveness for this purpose as yet. However, as nicotine is the common addictive substance inhaled through both smoking and vaping and is central to these pharmacotherapies' mechanisms of action, it is reasonable to assume that these interventions may also be effective in helping people to stop vaping nicotine. In licencing these pharmacotherapies for smoking cessation, their unintended effects have been taken into account and considered minimal relative to the risks of combustible tobacco smoking. As the risks of nicotine vaping are different from those of combustible tobacco smoking, it is important that the relative risks of these pharmacotherapies versus vaping are taken into account when considering them as vaping cessation aids.

Behavioural interventions, whether delivered via counselling or using digital delivery techniques, are usually based on a psychological theory of change [19]. For example, text messages may be developed to address different aspects of addictive behaviour, such as withdrawal and triggers to relapse, and thus to attempt to intervene with specific ‘behaviour change techniques’ [20]. It is generally accepted that nicotine addiction is a complex behaviour, and behavioural interventions will thus seek to address different aspects of the behaviour (e.g. motivation, self‐efficacy, beliefs) in order to influence it.

The evidence base for how alternative therapies (e.g. hypnosis; acupuncture) may work is somewhat unclear, with different theories suggesting how these therapies may work for some people. Personal beliefs can be powerful drivers of behaviour, thus ‘belief’ in a therapy, the placebo effect, or being persuaded to change (e.g. through hypnotherapy), could be potential mechanisms of change for some people.

### Why it is important to do this review

There is currently limited guidance based on direct evidence on: how to stop vaping nicotine; the most effective ways to ensure long‐term vaping cessation; or minimising the risk of tobacco smoking relapse and other unintended effects of treatment. A systematic review of the evidence, including literature published to September 2021, concluded that very little interventional research had been conducted, precluding any conclusions on the benefits and harms of vaping cessation interventions [21]. More recent reviews of the literature have concluded that there is still limited evidence on the best ways to support people to quit vaping, and that although interventions show promise, further studies are required [22, 23]. Vaping cessation is a growing area of research, with several trials completed since 2024 and many more underway.

## Objectives

To conduct a living systematic review assessing the benefits and harms of interventions to help people stop vaping compared to each other, placebo or no intervention.

To assess how these interventions affect the use of combustible tobacco, and whether effects vary based on participant characteristics.

## Methods

The living review format means that this review will be updated as new evidence becomes available that may change the existing conclusions. We conduct database searches monthly, contact authors of ongoing studies, and make our monthly search updates publicly available at www.cebm.ox.ac.uk/research/electronic‐cigarettes‐for‐smoking‐cessation‐cochrane‐living‐systematic‐review‐1. An update to the review is triggered when the accumulating evidence leads to changes in any one of the following:

- the direction of effect or clinical significance of the findings for one or more outcomes;
- the certainty (e.g. GRADE rating) of one or more outcomes; or
- the availability of studies investigating new settings, populations, interventions, comparisons, or outcomes.

When an update is triggered, we incorporate new data into meta‐analyses and tables in RevMan [24]. We follow the Methodological Expectations for Cochrane Intervention Reviews (MECIR) when conducting this review [25] and PRISMA 2020 for reporting [26].

For full methods relating to the living status of this review, see Supplementary material 7. For differences between iterations of this living systematic review and the published protocol [27], please see the list of all amendments logged via an open repository: doi.org/10.17605/OSF.IO/6AC9J.

### Criteria for considering studies for this review

#### Types of studies

Randomised controlled trials (RCTs) including cross‐over trials. We did not exclude studies based on year or language of publication.

#### Types of participants

People of any age, regardless of tobacco use status, using any kind of nicotine vape at baseline. 'Current vaping' was defined as per study authors, at entry into the study, and could include people concurrently smoking tobacco and vaping. We included studies that enrolled people regardless of vaping behaviour, as long as they provided a group of nicotine vape users with vaping cessation intervention(s) and collected relevant outcomes for the subset of the population considered to be current vape users.

We did not include studies conducted exclusively in people who did not vape nicotine (e.g. in people vaping tetrahydrocannabinol, or non‐nicotine vapes). Where studies did not define type of vaping, or included people who vaped both nicotine and other types of e‐liquid, we planned to include these studies, separating out and only extracting information on the nicotine vaping subgroup, where available. If separate data for this group were not available, we planned to test exclusion of the study in a sensitivity analysis. However, no such studies were included in this version of the review.

#### Types of interventions

Any intervention designed to support people who vape to stop vaping. This could include:

- behavioural interventions of any intensity, modality, or frequency, and from any provider;
- pharmacological interventions, such as cytisine, nicotine replacement therapy (NRT), varenicline, and bupropion, of any dosage or frequency;
- changes in characteristics of vapes, such as reductions in nicotine content;
- any combination of the above interventions.

If interventions were designed to both prevent vaping in people not currently vaping and to encourage cessation in people currently vaping, we planned to include these studies if the data on people using vapes at baseline could be separated out and at least one of the outcomes of interest was reported in this subset. However, no such studies were included in this version of the review.

### Outcome measures

#### Critical outcomes

- Vaping cessation at the longest follow‐up point, at least six months from the start of the intervention, measured on an intention‐to‐treat (ITT; including all participants in their originally assigned groups) basis using the strictest definition of abstinence, preferring biochemically validated results (self‐reported outcomes confirmed using biological tests) where reported.
- Change in combustible tobacco use (smoking) between baseline and the longest follow‐up point, at least six months from the start of the intervention. Combustible tobacco use includes tobacco cigarettes, loose roll‐your‐own, cigars, cigarillos, and pipe tobacco. Dependent on smoking status at baseline, this could be continued smoking, uptake of smoking, or smoking cessation. We measured these as defined by the study authors, using the strictest definition if multiple measures were reported, e.g. preferring continuous abstinence to point prevalence abstinence, and biochemically validated over self‐reported results for smoking cessation.
- Number of participants reporting serious adverse events (SAEs) at one week or longer (as defined by the study authors). If SAEs were reported at more than one time point, we used the measure at longest follow‐up.

#### Important outcomes

- Vaping cessation at the longest follow‐up point, at three or more but less than six months from the start of the intervention, measured as per critical vaping cessation outcome.
- Change in combustible tobacco use between baseline and the longest follow‐up point, three or more but less than six months from the start of the intervention, measured as per critical change in tobacco use outcome.
- Number of participants reporting adverse events (AEs) at one week or longer (as defined by the study authors), at the longest follow‐up point reported.
- Number of people vaping a substance other than nicotine at longest follow‐up, at three months follow‐up or longer.
- Changes in weight between baseline and longest follow‐up point, with the longest follow‐up at three months or longer.
- Changes in alcohol use between baseline and longest follow‐up point, with the longest follow‐up at three months or longer.
- Changes in the following measures at longest follow‐up (one week or longer):
  - carbon monoxide (CO), as measured through breath or blood;
  - blood pressure;
  - heart rate;
  - blood oxygen saturation;
  - lung function measures;
  - cotinine;
  - known toxins/carcinogens, as measured through blood or urine (examples of toxicant names and abbreviations are listed in Appendix 2 of our review on e‐cigarettes for smoking cessation) [18].

Studies needed to plan to measure at least one of the critical or important outcomes above to be eligible for inclusion.

### Search methods for identification of studies

#### Electronic searches

We conducted searches up to 1 July 2025, searching the following databases and employing the targeted search strategy in Supplementary material 1:

- Cochrane Central Register of Controlled Trials (CENTRAL; 2025, Issue 6) via CRS‐Web;
- MEDLINE (via Ovid SP, from 1 January 2004 to 1 July 2025);
- Embase (via Ovid SP, from 1 January 2004 to 1 July 2025);
- PsycINFO (via Ovid SP, from 1 January 2004 to 1 July 2025);
- ClinicalTrials.gov (via CENTRAL; 2025, Issue 6);
- World Health Organization International Clinical Trials Registry Platform (WHO ICTRP) (via CENTRAL; 2025, Issue 6).

Our initial search for this living systematic review was limited to 2004 onwards because vapes were not available before 2004. Following this initial search, we conducted monthly searches of the same databases on the first day of each month. These monthly searches were conducted in combination with the monthly searches for the Cochrane review of *'Electronic cigarettes for smoking cessation'* [18]. The search terms used in these searches are broad enough to retrieve studies eligible for either review, using free text and subject headings relating to vape use, alongside study design filters matching our inclusion criteria. All ongoing search strategies are listed in Supplementary material 1.

As part of our monthly screening process, all new records related to an included study are incorporated into our records for that study, together with any postpublication amendments, including expressions of concern, errata, corrigenda and retractions.

#### Searching other resources

We searched the reference lists of eligible studies found in the literature searches. We contacted authors of known and eligible studies for further information when needed. We also searched abstracts from the 2024 and 2025 Society for Research on Nicotine and Tobacco (SRNT) Annual Meetings.

### Data collection and analysis

#### Selection of studies

We screened citations retrieved by our searches using Covidence [28]. Two review authors independently checked the titles and abstracts for relevance against the eligibility criteria (NL, JHB, ARB, MC, ADW, TT, TRF, RB, CN). Any disagreements were resolved through discussion with a third review author. We obtained the full‐text versions of papers considered to be potentially relevant. Two review authors (of those listed previously) independently assessed the full‐text reports for inclusion in the review. Any disagreements were resolved through discussion with a third review author. Where necessary, we contacted study investigators for further information to aid our decision‐making. We recorded and reported reasons for excluding studies at the full‐text stage.

We screened and included studies reported in any language. Had it been necessary, we would have arranged for the translation of non‐English language papers. Where we found multiple citations relating to the same study, we grouped them into one study record with a single study ID.

#### Data extraction and management

For each included study, two review authors independently extracted data to be used in analyses (including covariates) and for risk of bias assessment (ARB, LB, ES, NL, CN, ADW). Study characteristics were extracted by a single review author. We cross‐checked dual extraction, with any disagreements between review authors resolved through discussion or by involving a third review author. Data extraction processes were carried out using Covidence and piloted before use. We imported the extracted and checked data into RevMan [24, 28]. See Supplementary material 8 for the information extracted from the included studies.

#### Risk of bias assessment in included studies

Two review authors independently assessed the risk of bias for each included study (NL, JHB, ARB, LB, ES, CN, ADW). We used the methods set out by the Cochrane Tobacco Addiction Group [29], which is based on the domains of the Cochrane RoB 1 tool [30]. This approach uses a domain‐based evaluation that addresses seven different areas: random sequence generation; allocation concealment; blinding of participants and providers; blinding of outcome assessment; incomplete outcome data; selective outcome reporting; and other potential sources of bias. We assigned a judgement (low, high, or unclear) for each domain. Any disagreements were resolved through discussion or by consulting a third review author when required.

Specific considerations about judgements for individual domains in this review are outlined below and are in line with our existing review of *‘Electronic cigarettes for smoking cessation’* [18].

- Blinding of participants and providers: we did not assess this domain for studies solely investigating behavioural interventions, as specific risk of bias guidance developed by the Cochrane Tobacco Addiction Group advises this, due to it being impossible to blind these types of interventions [29]. For studies of pharmacological interventions that did not use blinding, we considered studies at low risk of bias for this domain if the intervention was compared to a placebo or an active control of similar intensity, as we judge performance bias to be unlikely in this circumstance. However, if a study was unblinded, and the comparator group was a minimal‐intervention control or of lower intensity than the intervention group, we considered the study to be at high risk of bias for this domain.
- Blinding of outcome assessment: following the standard methods of the Cochrane Tobacco Addiction Group, we considered studies to be at low risk of detection bias if they assessed our primary outcome(s) objectively, or if participants received the same amount of face‐to‐face contact across relevant study groups, or both [29].
- Incomplete outcome data: again, following the standard methods of the Cochrane Tobacco Addiction Group, we rated studies at high risk of attrition bias if loss to follow‐up was greater than 50% overall, or if there was a difference in follow‐up rates of more than 20% between study arms at the longest follow‐up used in our analysis [29].

We judged studies to be at high risk of bias overall if they were rated at high risk in at least one domain, and at low risk of bias overall if they were judged to be at low risk across all domains evaluated. We judged the remaining studies to be at unclear risk of bias overall.

Where a study reported more than one of our outcomes of interest, we assessed risk of bias for our critical vaping and smoking outcomes only.

#### Measures of treatment effect

We calculated risk ratios (RR) and their 95% confidence intervals (CI; the range indicating where the true effect is likely to be) for dichotomous outcomes for each study. For continuous outcomes, we compared the difference between the relevant intervention and control groups using mean differences (MD) and 95% CI for each study.

#### Unit of analysis issues

We only consider combining trial arms if this is how the information is presented by study authors, or where there is no evidence of difference between similar trial arms for the outcome of interest. In this iteration of the review, we did this for two studies. In the case of Klein 2024 [31, 32, 33, 34, 35, 36, 37], for our combined NRT versus control comparison, we combined the combination NRT and combination NRT + text message intervention arms into one intervention arm, and the no/minimal support arm and the text message arm into one control arm. For Klein 2024 and our text message intervention versus control comparison, we combined the text message‐only intervention and the combination NRT + text message intervention into one intervention arm and the no/minimal support arm and the combination NRT arm into one control group. In both instances, this was because there was no evidence of an interaction between combination NRT and the text message‐based behavioural support. In the case of Michaud 2025 [38, 39, 40], for our media literacy e‐learning versus no/minimal support comparison, we combined the media literacy e‐learning + text messaging arm and the media literacy e‐learning + financial incentives + text messaging arm into one intervention arm, and the text messaging alone arm and the financial incentives + text messaging arm into one control arm. For our financial incentives versus no/minimal support comparison, we also combined the financial incentives + text messaging arm and the media literacy e‐learning + financial incentives + text messaging arm into one intervention arm, and the text messaging alone arm and the media literacy e‐learning + text messaging arm into one control arm. In both instances, there was no evidence of an interaction between the intervention components. Therefore, components appearing in both the intervention and control arms were deemed to cancel themselves out and the effect was deemed to be solely that of the additional component that was only present in one of the arms of the relevant comparison.

None of the studies included in this review were cluster‐randomised. Had any been cluster‐randomised, we would have assessed whether study authors adjusted for clustering, and whether this had an impact on the overall result. Had clustering had little impact on the results, we would have used unadjusted quit‐rate data; however, if clustering had impacted results, we would have adjusted for this using the intraclass correlation (ICC) reported by the paper (or where this was not provided, one used in a similar study). Should we include cluster‐RCTs in the future, this is the approach we will take.

In the case of eligible cross‐over trials (ensuring that the first assignment period was sufficiently long to meet our inclusion criteria), we planned to extract and report on results at the end of the first assignment period, where these were available. At this point, we have not identified any eligible cross‐over trials, but will use this approach if we identify them in future.

#### Dealing with missing data

When assessing change in tobacco use, we used the standard Cochrane Tobacco Addiction Group approach, treating participants with missing data as still smoking. We made the same assumption for vaping when assessing whether vaping cessation had taken place, assuming those lost to follow‐up were continuing to vape.

We based the proportion of people affected by SAEs/AEs on the number of people available for follow‐up, and not the number randomised, where reported.

For continuous outcomes, we also used complete‐case data and did not attempt to impute missing values. Where possible, we extracted data demonstrating the change in the outcome between baseline and follow‐up and compared this change data between study arms. However, where this was not reported, we compared the data at follow‐up only between study arms.

#### Reporting bias assessment

As noted above, we took selective reporting into consideration as part of our risk of bias assessment for each study. When interpreting the results, we accounted for this, and also planned to account for potential findings of studies that we knew to have taken place, but for which we did not have results (however, we did not become aware of any such studies).

Reporting bias can be assessed using funnel plots, where 10 or more studies contribute to a given outcome [41]. None of our meta‐analyses included 10 or more studies. However, where 10 or more studies are included in an analysis in future updates, we will generate funnel plots and visually inspect them for asymmetry.

#### Synthesis methods

We took the clinical variance of studies into account when grouping them for analyses. Studies were split into comparisons based on intervention and comparator type (e.g. studies investigating behavioural interventions were not grouped with those investigating pharmacological interventions, and different types of pharmacological interventions were grouped separately).

We carried out pairwise meta‐analyses for comparisons where there was more than one eligible RCT. We used random‐effects Mantel‐Haenszel models to calculate pooled RR with 95% CI for dichotomous outcomes. For continuous outcomes, we calculated pooled MDs, using the inverse variance approach (also with 95% CI). (Note: in future iterations of this review, we will use random‐effects Mantel‐Haenszel models to calculate pooled risk difference (RD) with 95% CI for our SAE outcome only.)

Where meta‐analysis was not possible or appropriate, we synthesised data narratively and using effect direction plots [41].

#### Investigation of heterogeneity and subgroup analysis

We assessed clinical and methodological diversity between studies to guide decisions as to whether data should be pooled. Where we pooled studies using meta‐analysis, we calculated I^2^ statistics [30, 41]. We considered a value greater than 50% as evidence of substantial heterogeneity (difference between the results of studies included in the analysis). Where I^2^ exceeded 75%, we considered whether it was appropriate to present a pooled result based upon the directions of the contributing effects (e.g. where all studies showed a benefit of an intervention, it could still be deemed appropriate to present a pooled estimate despite differing magnitudes of effect across studies).

We planned to use subgroup analyses to investigate the following variables as potential moderators of effects:

- Vaping/smoking history. We expected that some studies may have been carried out in people who had never smoked and some in people who had used vapes to reduce or stop smoking.
- Frequency of vaping. We expected interventions may operate differently based on the levels of vaping at baseline.
- Age. Some interventions may specifically be aimed at young people, and there are specific concerns around vaping in young people who have never smoked. It may be that interventions in young people target different elements of behaviour than those in adults, and we planned to test whether intervention effects differed in younger people compared to adults.
- Relevant intervention characteristics, such as the intensity, provider, or modality of behavioural interventions, or the dose, duration, or timing of pharmacological interventions.
- Interventions conducted in specific groups, e.g. based on level of nicotine addiction.

However, due to the small number of studies in all meta‐analyses, subgrouping was only possible by participant age category for our varenicline versus placebo and text message‐based intervention versus no/minimal support comparisons. This was carried out for illustrative purposes rather than to inform any hypotheses due to the lack of statistical power. However, as more studies contribute to subgroup analyses, we will assess their significance based on whether the effects of subgroups would lead to differing clinical interpretations and using the I^2^ statistic (interpreted according to the thresholds discussed earlier in this section). We will seek to conduct the subgroup analyses specified above in further updates of the review where possible.

None of our analyses included enough studies to make meta‐regression possible. However, we may use meta‐regression to investigate the following variables as moderators of our aggregate outcomes in future updates of this review:

- Average age of participants in the study
- Length of time vaping at baseline (as reported by study authors)

We extracted any reports of analyses of associations between outcomes and our moderators of interest. We synthesised these narratively using effect direction plots [41].

##### Equity‐related assessment

We did not plan to investigate health inequity in this review, beyond the investigations specified above.

#### Sensitivity analysis

Where possible, we carried out sensitivity analyses for all meta‐analyses by removing studies:

- judged to be at overall high risk of bias;
- funded by the manufacturer/provider of the intervention.

We judged effects sensitive to these exclusions if the resulting effect led to a different clinical interpretation than the original effect.

We had also planned to carry out sensitivity analyses removing studies where not all participants vaped nicotine (where we were unable to separate out those people who only vaped nicotine); however, we did not include any studies that explicitly stated that participants were vaping non‐nicotine liquids, so this was not relevant. We will conduct this analysis in future updates, as appropriate.

#### Certainty of the evidence assessment

We (NL, JHB, ARB) carried out GRADE assessments and created summary of findings tables for our critical outcomes (see Critical outcomes) using GRADEpro GDT software [42]:

- vaping cessation at six months follow‐up or longer;
- change in combustible tobacco use at six months follow‐up or longer;
- number of people reporting SAEs at one week follow‐up or longer.

We generated a summary of findings table for each of the following comparisons (testing single components and measuring at least one of our critical outcomes):

- combination NRT versus no/minimal support;
- cytisine versus placebo;
- varenicline versus placebo;
- nicotine concentration and vaping reduction versus no/minimal support;
- text message‐based interventions versus no/minimal support for young people.

Following standard Cochrane methodology, we used the five GRADE considerations (study limitations, consistency of effect, imprecision, indirectness, and publication bias) to assess the certainty of the body of evidence for each outcome, and to draw conclusions about the certainty of the evidence within the text of the review. Any disagreements were resolved through discussion.

### Consumer involvement

We held a consumer planning consultation in June 2023. At this workshop, participants concluded that it would be clearer to use the term 'vape' rather than 'e‐cigarette' in the review title. We amended the title in response to this feedback. We held a second workshop and online consultation between October and December 2024 to discuss a dissemination plan for the results of our review. Our conclusions have not changed since the last version of our review, so we did not hold a further consultation to inform dissemination messages. However, we will hold a further consumer consultation in 2026 to discuss future planning for this project and the interpretation of any changes to conclusions. This will incorporate an evaluation of the living systematic review approach, dissemination used so far, and suggestions for improvements and new ways of working. This will allow us to assess whether it is appropriate and useful to continue the review. We will run a survey disseminated on public‐facing forums, such as Gumtree, Bluesky and X to gain the input of people who may not volunteer to be part of a more formal panel or attend a workshop in person (consumer input has indicated that different groups may be comfortable with different levels of involvement, and we want to be as inclusive as possible).

Our consumer panel have diverse vaping and smoking experiences and are from differing social backgrounds. All consumers are reimbursed for their time. We have a lead consumer contributor (CJ) who has experience of smoking combustible cigarettes and using vapes. Through phone, email, online, and in‐person project meetings, CJ is contributing to the proposed work, meeting with the chair to discuss meeting agendas beforehand, and to debrief afterwards.

We are using Cancer Research UK’s consumer toolkit and Cochrane consumer resources to assist our consumer involvement.

## Results

### Description of studies

#### Results of the search

For this updated living systematic review, our bibliographic database searches identified 2076 deduplicated records (Figure). We screened the titles and abstracts, and retrieved the full texts of 95 potentially relevant records for comprehensive eligibility assessment. We excluded 51 full texts with reasons (see Excluded studies) and included 44 full‐text references, representing six newly included studies (Evins 2025 [43, 44, 45, 46, 47]; Heffner 2025 [48, 49]; Klemperer 2025 [50, 51]; Michaud 2025; NCT05140915 [52, 53]; Palmer 2025 [54, 55, 56]), 18 ongoing studies, and 20 articles linked to the included studies – these secondary study reports are linked to the included studies in the references section of this review.

**1:**
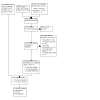
PRISMA study selection flow diagram *Characteristics of 18 excluded studies are summarised in Supplementary material 3.

We brought forward nine included studies and 14 ongoing studies from the previous review version [57]. In total, this updated living systematic review includes 15 studies, with 32 ongoing studies. See Supplementary material 2; Supplementary material 3; Supplementary material 4

#### Included studies

We have included 15 studies that are summarised below and in Table; of these, six are new to this update and nine are brought forward from the previous review version [57]. Further details on each of these 15 included studies are available via Supplementary material 2.

**1:** Overview of included studies and syntheses (all recruited participants vaping nicotine and motivated to quit vaping)

Study ID	**Number randomised**	**Study arms**	**Length of follow‐up (months)**	**Overall RoB judgement**	**Age of participants (years)**	**Participants' baseline 1) tobacco smoking; 2) vaping behaviour**	**Funded by manufacturer or provider of intervention**	**Country**	**Outcomes reported**
Caponnetto 2023	140	1) Varenicline 2) Placebo tablet	6	Low	18 and over	1) Not smoking 2) Daily vaping	Yes	Italy	Vaping cessation at 6 months; vaping cessation at between 3 and 6 months; AEs; SAEs; weight; blood pressure; heart rate
Evins 2025	261	1) Varenicline 2) Placebo tablet	6	Low	16 to 25	1) No prior regular smoking 2) Vaping 5 or more days per week	No	USA	Vaping cessation at 6 months; vaping cessation at between 3 and 6 months; SAEs; AEs
Fucito 2024	40	1) Varenicline 2) Placebo	3	Unclear	18 and over	1) Not smoking 2) Daily vaping	No	USA	Vaping cessation at between 3 and 6 months; SAEs; AEs
Graham 2021	2588	1) Text message (*This is Quitting*) 2) Control (assessment only)	7	Low	18 to 24	1) Smoking or not smoking 2) Vaping in past 30 days	Yes	USA	Vaping cessation at 6 months+; change in combustible tobacco use at 6 months+
Graham 2024	1503	1) Text message (*This is Quitting*) 2) Control (assessment only) 3) Waitlist	7	Low	13 to 17	1) Smoking or not smoking 2) Past 30 day use of vapes	Yes	USA	Nicotine vaping cessation at 7 months; SAEs; AEs
Heffner 2025	61	1) App‐based + text messaging intervention 2) Control	3	High	18 to 30	1) Smoking or not smoking 2) Weekly user of vapes	No (ALG employed by Truth Initiative)	USA	Abstinence from all nicotine and tobacco products at 3 months; SAEs; AEs
Klein 2024	508	1) NRT vs text message 2) NRT + text message 3) Control	3	High	18 to 24	1) Not smoking 2) Regular vaping	No	USA	Vaping cessation at between 3 and 6 months; SAEs; AEs
Klemperer 2025	396	1) NRT + text messages to quit CCs only 2) NRT + text messages to quit CCs and ECs simultaneously 3) text messages alone to quit CCs only 4) text messages alone to quit CCs and ECs simultaneously We compared arms 2 and 4.	6	High	18 to 29	1) Smoking 2) Vaping	No	USA	Vaping cessation at 6 months; vaping cessation at between 3 and 6 months; SAEs; AEs; combustible tobacco abstinence at 6 months or longer; combustible tobacco abstinence at between 3 and 6 months
Michaud 2025	40	1) Media literacy e‐learning 2) Financial incentives 3) Media literacy e‐learning + financial incentives 4) Control	3	Unclear	19 to 29	1) Unclear smoking status 2) Vaping in past 30 days	No	USA	Vaping cessation at between 3 and 6 months
NCT04602494	4	1) Varenicline 2) Placebo 3) Monitoring only	3 (planned to 6 but abandoned due to dropout)	High	18 to 24	1) Not smoking 2) Daily or near daily vaping	No	USA	Vaping cessation at 3 months (had planned at 3 to 6 months, abandoned due to dropout); AEs; SAEs
NCT05140915	71	(1) Text‐based (peer messages, peer coaching and gamification (unclear if delivered via text) 2) Control	6	Unclear	13 to 19	1) Unclear smoking status 2) Current vape user	No	USA	Vaping cessation at between 3 and 6 months; SAEs; AEs
Palmer 2023	30	1) NRT 2) Control (quitline referral)	2	High	18 and over	1) Smoking or not smoking 2) Daily vaping	No	USA	SAEs; AEs
Palmer 2025	46	1) Standard‐dose combination NRT 2) Mid‐dose combination NRT 3) High‐dose combination NRT	2	Unclear	18 and over	1) Smoking 2) Vaping (Dual users)	No	USA	SAEs; AEs
Rigotti 2024	160	1) Cytisinicline 2) Placebo	4	Low	18 and over	1) Not smoking 2) Daily vaping	Yes	USA	Vaping cessation at between 3 and 6 months; tobacco use at FU; SAEs; AEs; blood pressure; heart rate; cotinine
Sahr 2021	24	1) NRT + behavioural 2) Vaper‐taper + behavioural 3) Self‐taper	6	High	18 and over	1) Not smoking 2) Vaping at least 4 days/week	No	USA	Vaping cessation at 6 months; vaping cessation at between 3 and 6 months; weight; blood pressure; heart rate

AE: adverse event CC: combustible cigarettes EC: electronic cigarettes FU: follow‐up NRT: nicotine replacement therapy RoB: risk of bias SAE: serious adverse event

##### Study types

All 15 included studies were RCTs. Two studies employed randomised 2 x 2 factorial study designs (Klein 2024; Klemperer 2025).

##### Participants

The 15 included studies represented 5800 participants relevant to this review. Fourteen studies were conducted in the USA and one in Italy. All studies were conducted in people who were currently using nicotine vapes. In 12 studies, it appeared that participants were interested or motivated to stop using vapes; of the remainder, Heffner 2025 stated that participants of all motivations were included. Klemperer 2025 recruited people who smoked tobacco and used vapes and were motivated to stop tobacco use (but were not recruited based on their motivation to stop vape use). For NCT05140915 the motivation of participants to quit vaping was unclear. Seven studies were carried out among participants not using tobacco cigarettes at baseline (one of these studies purposely recruited young people who had never smoked; Evins 2025). In a further four studies, some of the participants were dual users of vapes and tobacco cigarettes. In two studies, all participants were dual users and in two other studies, tobacco cigarette use at baseline was unclear. Twelve studies were conducted amongst participants over the age of 18; five of these were exclusively in adults aged 18 to 29. Two studies included some participants under 18 years; Evins 2025 recruited 16‐ to 25‐year‐olds and NCT05140915 recruited 13‐ to 19‐year‐olds. Graham 2024 [58, 59] solely recruited participants aged under 18 years (13‐ to 17‐year‐olds).

##### Interventions and comparators

Across the 15 included studies, the following pharmacological interventions were investigated: varenicline, cytisinicline (also known as cytisine and hereafter referred to as such), and combination NRT. The behavioural interventions tested included text message‐based interventions, a smartphone app + text messaging intervention, an intervention focused on reducing the nicotine content of vapes and time spent using them, a media literacy e‐learning intervention, and financial incentives.

Four studies tested varenicline versus placebo. In three of these studies, varenicline or placebo was provided for three months and follow‐up was at six months (Caponnetto 2023 [60, 61, 62, 63, 64]; Evins 2025; NCT04602494 [65]). In the fourth study, varenicline was provided for eight weeks and follow‐up was at 12 weeks (Fucito 2024 [66, 67, 68]).

One study compared cytisine to placebo tablets for 12 weeks with a 16‐week follow‐up period (Rigotti 2024 [69, 70, 71]).

Palmer 2023 [72] followed participants for two months and compared four weeks of combination NRT to referral to a quitline (control). Klemperer 2025 also investigated the effects of combination NRT on stopping vaping in two of the four study arms (the other two arms were not relevant to this review as they only focused on stopping combustible tobacco use). One of the relevant arms provided participants with 12‐week combination NRT alongside text message support; this was compared to a text message‐only control arm. Participants were involved up until a six‐month follow‐up point. A three‐month study recruiting 18‐ to 24‐year‐olds compared four study arms: 1) combination NRT + coaching calls; 2) text message‐based intervention + coaching calls; 3) combination NRT + text message‐based intervention + coaching calls; 4) coaching calls alone (Klein 2024). The NRT was supplied for eight weeks. A final study that investigated combination NRT provided a four‐week supply to all participants, but varied the dosage across study arms (Palmer 2025). All three study arms received 4 mg nicotine lozenges; however, one study arm received 21 mg patches, the second 21 mg +14 mg patches, and the third 2 x 21 mg patches. Follow‐up was six weeks.

Graham 2021 [73, 74, 75, 76, 77] assessed a once‐a‐day text message‐based intervention (*'This is Quitting'*) for vaping cessation among young adults (18 to 24) compared to an assessment‐only control over seven months. A second study also investigated the *'This is Quitting'* text message‐based intervention compared to assessment‐only control over seven months in 13‐ to 17‐year‐olds (Graham 2024).

NCT05140915 looked at a different text messaging intervention that provided text messages written by peers – current and former adolescent e‐cigarette users – and tailored by age and readiness to stop vaping. The intervention also included gamification; however, it is not clear from the information provided whether this was provided through text messaging or another modality. This intervention was compared to printed materials on vaping cessation. The investigators planned to follow‐up participants for six months; however, only three‐month data were available at the time of writing.

Heffner 2025 investigated a smartphone app and text messaging programme called *'ACT on Vaping'* that consisted of six interactive sessions, compared to an assessment‐only control. Participants were followed for three months in total.

Sahr 2021 [78] looked at the effect of two vaping cessation methods over six months of follow‐up: 1) reducing both nicotine concentration and time spent vaping over 12 weeks and 2) 12 weeks of combination NRT. This was explored in a three‐armed study where the two interventions were compared to a minimal support control arm.

Michaud 2025 split participants into four study arms and followed them for 12 weeks. All of these arms received text message support; one arm also received media literacy e‐learning lessons (designed to prevent and reduce vaping), another received financial incentives for providing saliva samples and for test results indicating vaping cessation (participants could earn a total of USD 70), and another had both the media literacy e‐learning and the incentives, alongside text messaging.

Overall, six studies followed up participants for six months or longer, seven for between three and less than six months (NCT04602494 planned to follow up participants for six months but was terminated early and NCT05140915 had only reported three‐month data to date, but planned to follow up to six months and could report this in due course), one for two months and one for six weeks. Further details on the intervention and comparator groups for each study can be found in the 'Characteristics of included studies' table in Supplementary material 2.

##### Outcomes

Of the critical outcomes (see Critical outcomes):

- Six studies reported data on vaping cessation at six months or longer (Caponnetto 2023; Evins 2025; Graham 2021; Graham 2024; Klemperer 2025; Sahr 2021).
- Two studies reported data on change in combustible tobacco use between baseline and six months or longer (Graham 2021; Klemperer 2025).
- 11 studies reported data on the number of participants reporting SAEs at one week or longer (Caponnetto 2023; Evins 2025; Fucito 2024; Graham 2024; Heffner 2025; Klein 2024; Klemperer 2025; NCT04602494; NCT05140915; Palmer 2023; Rigotti 2024).

Of the important outcomes (see Important outcomes):

- 11 studies reported data on vaping cessation at three or more, but less than six, months from the start of the intervention (Caponnetto 2023; Evins 2025; Fucito 2024; Heffner 2025; Klein 2024; Klemperer 2025; Michaud 2025; NCT04602494; NCT05140915; Rigotti 2024; Sahr 2021).
- Two studies reported on change in combustible tobacco product use between baseline and three or more, but less than six, months from the start of the intervention (Rigotti 2024; Klemperer 2025).
- 11 studies reported on the number of participants reporting adverse events at one week or longer in both arms (Caponnetto 2023; Evins 2025; Fucito 2024; Graham 2024; Heffner 2025; Klein 2024; Klemperer 2025; NCT04602494; NCT05140915; Palmer 2025; Rigotti 2024). One further study reported AEs for the intervention arm only (Palmer 2023).
- No studies reported on the number of people vaping a substance other than nicotine at longest follow‐up, at three months follow‐up or longer.
- Two studies reported weight at follow‐up (Caponnetto 2023; Sahr 2021).
- No studies reported alcohol use status. One study, NCT04602494, stated that they would report on substances other than nicotine consumed (including tobacco and alcohol use); however, this was not reported as there were issues with recruitment and follow‐up.
- No studies reported carbon monoxide (CO), measured through breath or blood; blood oxygen saturation; lung function measures or known toxins/carcinogens.
- Three studies reported blood pressure (Caponnetto 2023; Rigotti 2024; Sahr 2021), three studies reported heart rate (Caponnetto 2023; Rigotti 2024; Sahr 2021), and one study reported cotinine (Rigotti 2024).

##### Funding

Of the 15 included studies, four were funded by the manufacturer or provider of the intervention (Caponnetto 2023; Graham 2021; Graham 2024; Rigotti 2024).

Caponnetto 2023 was a trial of varenicline versus placebo, funded by GRAND (Global Research Award for Nicotine Dependence), an independently reviewed competitive grants programme funded by Pfizer Inc (USA). Pfizer is the manufacturer of Chantix/Champix (brand names for varenicline). This study was also funded by ECLAT Srl., which provides consultancy and develops, produces, and markets services and products in the field of combustion‐free devices as an alternative to traditional cigarettes. Rigotti 2024 studied cytisine versus placebo and was funded by Achieve Life Sciences, who are co‐developing a cytisine product called cytisinicline. Both Graham 2021 and Graham 2024 investigated a text message programme (*'This is Quitting'*) provided by the Truth Initiative, who also funded the studies.

#### Excluded studies

We excluded 51 studies at the full‐text screening stage. Reasons for exclusion are provided in the PRISMA flow diagram (Figure). Amongst these 51 studies, the most common reason for exclusion was ineligible intervention.

We summarised 18 studies that appeared to potentially meet the inclusion criteria, but which were ultimately excluded, in the 'Characteristics of excluded studies' table (Supplementary material 3), with reasons for exclusion.

### Risk of bias in included studies

We judged five studies to be at low risk of bias (Caponnetto 2023; Evins 2025; Graham 2021; Graham 2024; Rigotti 2024), four at unclear risk (Fucito 2024; Michaud 2025; NCT05140915; Palmer 2025) and six at high risk (Heffner 2025; Klein 2024; Klemperer 2025; NCT04602494; Palmer 2023; Sahr 2021).

The details of the risk of bias judgements for each domain for each included study can be found in the 'Characteristics of included studies' in Supplementary material 2. Judgements for individual included studies in Figure.

**2:**
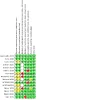
Risk of bias judgements for each domain of the included studies

#### Allocation

We judged eight studies to be at low risk of bias and seven studies at unclear risk of bias for random sequence generation and allocation concealment. The studies judged to be at low risk of bias for random sequence generation used methods that were considered truly random, such as a random number generator or computer‐based randomisation systems, and reported this in full. In the studies judged to be at low risk of allocation bias, concealment methods were used that meant that participants and study staff were unaware of the group to which the participants would be assigned. Where ratings were unclear, this was because reports did not provide enough information to make a judgement.

#### Blinding

We judged seven studies to be at low risk of performance bias. Matched placebos were used in five studies. As per protocol, we did not assess blinding of participants and personnel (performance bias) for studies solely investigating behavioural interventions, as it is impossible to blind these types of interventions. Therefore, five studies were not judged for this domain (Graham 2021; Graham 2024; Heffner 2025; Michaud 2025; NCT05140915). We judged three studies to be at high risk of bias, as the study arms received support of notably different intensities (Palmer 2023) or NRT was not investigated using a placebo control and there was a lack of biochemical verification of the vaping cessation outcome (Klein 2024; Klemperer 2025).

For detection bias, we judged 14 studies to be at low risk and one at high risk. We deemed Sahr 2021 high risk as vaping cessation was not biochemically validated and there was differential face‐to‐face contact between study arms.

#### Incomplete outcome data

We judged 13 studies to be at low risk of attrition bias. We judged two studies to be at high risk, as in NCT04602494 all participants in the control group were lost to follow‐up, and in Heffner 2025 there was a substantial difference in the amount of dropout between arms (8/31 versus 1/30).

#### Selective reporting

We judged 13 studies to be at low risk bias for selective reporting. We judged two studies to be at unclear risk. In one, the primary outcome had already been reported in a conference abstract; however, full results were not yet available, but may become so in due course (Michaud 2025). We could not find evidence that the second study was preregistered (Palmer 2023).

#### Other potential sources of bias

We judged all 15 of the included studies to be at low risk of any other sources of bias.

### Synthesis of results

Data on our outcomes of interest are summarised by comparison below and in Table (combination NRT compared to no/minimal support for nicotine vaping cessation); Table (cytisine compared to placebo for nicotine vaping cessation); Table (varenicline compared to placebo for nicotine vaping cessation); Table (nicotine/vaping reduction compared to no/minimal support for nicotine vaping cessation); and Table (text message‐based interventions compared to no/minimal support for nicotine vaping cessation). Analyses are presented below and in Supplementary material 5.

#### Pharmacotherapy interventions versus controls

##### Combination NRT

###### Critical outcomes

The pooled point estimate for two studies (Klemperer 2025; Sahr 2021), both rated at high risk of bias, indicated no clear evidence of higher or lower nicotine vaping cessation rates at six‐month follow‐up in people randomised to receive combination NRT versus no/minimal support. The results were imprecise, with 95% CIs incorporating the possibility of no difference between arms and both higher and lower quit rates in the combination NRT arm (RR 0.96, 95% CI 0.73 to 1.25; I^2^ = 0%; 214 participants; very low‐certainty evidence; Analysis 1.1). See Figure.

**3:**
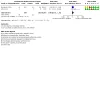
Combination NRT versus no/minimal support for nicotine vaping cessation

Klemperer 2025 (high risk of bias) was the only study in this comparison to assess change in combustible tobacco use, through gathering data on tobacco abstinence at six months. The point estimate was close to the null with an imprecise 95% CI, incorporating benefit, harm and no effect of combination NRT (RR 0.99, 95% CI 0.71 to 1.37; 198 participants; very low‐certainty evidence; Analysis 1.2).

Two studies (Klein 2024; Klemperer 2025), both rated at high risk of bias, reported no SAEs in either the combination NRT or control arms (with behavioural support matched to their respective intervention arms). Therefore, it was not possible to calculate a relative effect. We deemed this evidence to be of very low certainty (706 participants; Analysis 1.3).

###### Important outcomes

Three studies (all high risk of bias) reported nicotine vaping cessation rates between three and six months follow‐up (Klein 2024; Klemperer 2025; Sahr 2021). When pooled, the effect estimate indicated similar rates of quitting in the intervention and control groups; however, the 95% CI also encompassed the potential for both benefit and harm of combination NRT (RR 1.01, 95% CI 0.75 to 1.35; 722 participants; Analysis 1.4). As well as imprecision, this analysis was limited by moderate statistical heterogeneity (I^2^ = 54%).

Klemperer 2025 (high risk of bias) was the only study that reported change in combustible tobacco use between three and six months of follow‐up. The effect estimate suggested that there was a higher chance of tobacco abstinence in the combination NRT arm, but this result was imprecise with 95% CIs also incorporating the null and a possibility of higher quit rates in the control arm (RR 1.65, 95% CI 0.86 to 3.16; 198 participants; Analysis 1.5).

Klein 2024 and Klemperer 2025 (both high risk of bias) reported non‐serious AEs. There was evidence of high statistical heterogeneity across studies (I^2^ = 83%), driven by differences in the direction of effect; therefore, we do not present the pooled result (577 participants; Analysis 1.6).

Sahr 2021 (16 participants; high risk of bias) also reported weight (lbs), systolic blood pressure (mmHg), and heart rate (bpm). In all cases, results were imprecise with 95% CI incorporating potential increases, decreases, and no change in the combination NRT arm versus the no/minimal support arm. In the case of weight and systolic blood pressure, the point estimates indicated higher figures in the combination NRT arm at follow‐up (MD 33.93 lbs, 95% CI ‐17.57 to 85.41; 11 participants; Analysis 1.7; MD 8.32 mmHg, 95% CI ‐3.98 to 20.62; 14 participants; Analysis 1.8), whereas in the case of heart rate, lower bpm at follow‐up was reported in the combination NRT arm (‐4.23 bpm, 95% CI ‐24.29 to 15.83; 14 participants; Analysis 1.9).

None of our other important outcomes were reported in the studies eligible for this comparison.

##### Cytisine

Only one study, judged to be at low risk of bias and funded by the intervention manufacturer, contributed to the comparison of cytisine versus placebo (Rigotti 2024).

###### Critical outcomes

Rigotti 2024 (low risk of bias) reported one of our critical outcomes: number of participants reporting SAEs. Of the 159 participants in the trial, none reported SAEs (Analysis 2.1). Therefore, it was not possible to calculate a relative effect, and we deemed the evidence to be of low certainty due to imprecision.

Nicotine vaping cessation and change in combustible tobacco use at six‐month follow‐up or longer were not measured.

###### Important outcomes

Rigotti 2024 measured nicotine vaping cessation at four months follow‐up and found that more people stopped vaping in the cytisine group than in the placebo control group; however, the 95% CI incorporated the null and a potential benefit of placebo (RR 1.77, 95% CI 0.82 to 3.82; 160 participants; Analysis 2.2). This was the only study that reported change in combustible tobacco use; no participants were smoking at study baseline and their use of combustible tobacco was monitored to four‐month follow‐up. More participants were smoking combustible tobacco at follow‐up in the cytisine arm; however, the 95% CI was wide and incorporated both the null and potentially higher smoking rates in the placebo arm (RR 0.66, 95% CI 0.24 to 1.81; 160 participants; Analysis 2.3). Eleven of the 14 participants who reported smoking across study arms (78.6%) had previously smoked; this was not broken down by study arm.

There was no clear evidence that the number of participants reporting AEs differed between arms in Rigotti 2024, with the 95% CI incorporating benefit, harm, and no effect (RR 0.93, 95% CI 0.68 to 1.27; 159 participants; Analysis 2.4).

Rigotti 2024 also reported mean change in systolic and diastolic blood pressure (mmHg), mean change in heart rate (bpm), and cotinine (ng/mL; a metabolite of nicotine) levels at follow‐up. For all of these outcomes, 95% CIs were wide and showed no clear effect of intervention or control. However, for change in systolic blood pressure (MD 0.90, 95% CI ‐3.35 to 5.15; 130 participants; Analysis 2.5) and heart rate (MD 0.60, 95% CI ‐3.84 to 5.04; 130 participants; Analysis 2.7), the effect estimate suggested lower, more favourable values in the placebo arm, whereas for change in diastolic blood pressure (MD ‐2.50, 95% CI ‐5.72 to 0.72; 130 participants; Analysis 2.6) and cotinine values at follow‐up (MD ‐29.95, 95% CI ‐104.05 to 44.15; 126 participants; Analysis 2.8), the effect estimate suggested lower, more favourable values in the cytisine arm.

No further important outcomes were reported by Rigotti 2024.

##### Varenicline

###### Critical outcomes

Two studies, both judged at low risk of bias, eligible for the varenicline versus placebo comparison measured vaping cessation at six months or longer (Caponnetto 2023; Evins 2025). Caponnetto 2023 was funded by the manufacturer of the intervention. Both studies reported evidence that more participants quit nicotine vaping in the varenicline arms than in the placebo comparator arms (RR 2.71, 95% CI 1.33 to 5.49; 315 participants; Analysis 3.1). See Figure. A moderate amount of statistical heterogeneity was detected (I^2^ = 48%), which could be at least partially as a result of the different age ranges recruited into the studies (18+ years in Caponnetto 2023 and 16 to 25 years in Evins 2025); however, there could be other moderating factors and the subgroups are too underpowered to inform a hypothesis. We deemed the evidence to be of low certainty due to a low number of events across study arms (67 participants). We did not downgrade for heterogeneity as both studies showed a benefit of the intervention.

**4:**
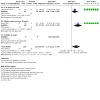
Varenicline versus placebo for nicotine vaping cessation

Four studies reported the number of participants reporting SAEs (Caponnetto 2023; Evins 2025; Fucito 2024; NCT04602494). Two of these studies (one judged at high risk of bias and one at unclear risk of bias) reported no SAEs in either the varenicline or control arms (Fucito 2024; NCT04602494). Therefore, only two studies (Caponnetto 2023; Evins 2025; both low risk of bias) contributed to the effect estimate, which included 304 participants for this outcome (RR 2.82, 95% CI 0.45 to 17.59; Analysis 3.2). No statistical heterogeneity was detected (I^2^ = 0%) and removing Caponnetto 2023 in a sensitivity analysis, due to it being funded by the intervention manufacturer, had no meaningful effect on the interpretation of the outcome. The evidence was judged to be low certainty due to the very small number of events and resulting imprecision.

None of the studies included in this comparison reported change in combustible tobacco use at six‐month follow‐up or longer.

###### Important outcomes

Four studies reported vaping cessation at between three and six months follow‐up (Caponnetto 2023; Evins 2025; Fucito 2024; NCT04602494). Pooling these studies (357 participants) resulted in an RR of 2.04 and a 95% CI of 1.11 to 3.75, indicating higher quit rates in the varenicline groups (Analysis 3.3). There was moderate statistical heterogeneity (I^2^ = 56%). When the only study judged to be at high risk of bias was removed from the analysis (NCT04602494), this resulted in no change in the interpretation of the effect. However, removing the only study funded by the intervention manufacturer (Caponnetto 2023) resulted in an RR of 1.79 and a 95% CI of 0.62 to 5.18, introducing uncertainty in the possibly positive effect. However, this result should be treated with caution due to the small number of studies and events and the overlap in the CI resulting from the main analysis and the sensitivity analysis.

We examined subgroups of the studies according to the age of participants; Evins 2025 recruited participants 16 to 25 years of age and the remaining studies recruited participants aged 18 and over. There was substantial statistical heterogeneity in subgroup effects (I^2^ = 77%), but due to the small number of studies, with only a single study in one of the subgroups, this result should not be used to inform a hypothesis regarding the moderating effect of age.

The same four studies reported the number of participants reporting AEs. The pooled estimate was subject to some imprecision with the 95% CI incorporating both potential harm and no effect of varenicline (RR 1.10, 95% CI 0.98 to 1.24; I^2^ = 0%; 304 participants; Analysis 3.4). Removing the only study judged to be at high risk of bias and the only study funded by the intervention manufacturer in separate sensitivity analyses resulted in similar point estimates, favouring placebo, but increased the imprecision to incorporate the potential for more AEs in the placebo arm (RR 1.11, 95% CI 0.95 to 1.31; RR 1.19, 95% CI 0.85 to 1.66, respectively).

Caponnetto 2023 alone reported weight (lbs), systolic and diastolic blood pressure (mmHg), and heart rate (bpm) at six months follow‐up. For all of these outcomes, the 95% CIs were wide and incorporated potential increases, decreases, and no difference in the varenicline arm versus the placebo arm. However, the point estimates for weight (MD ‐3.30 lbs, 95% CI ‐16.00 to 9.40; 95 participants; Analysis 3.5), systolic blood pressure (MD ‐1.60 mmHg, 95% CI ‐4.93 to 1.73; 95 participants; Analysis 3.6), and heart rate (MD ‐2.2 bpm, 95% CI ‐6.6 to 2.1; 95 participants; Analysis 3.8) indicated lower values in the varenicline group and the point estimate for diastolic blood pressure indicated higher values in the varenicline group (MD 0.80 mmHg, 95% CI ‐2.68 to 4.28; 95 participants; Analysis 3.7).

None of our other important outcomes were reported.

#### Behavioural interventions versus no/minimal behavioural support

##### Nicotine/vaping reduction

###### Critical outcomes

One very small study, judged to be at high risk of bias, compared a behavioural intervention versus minimal support (referral to a tobacco quitline) in reducing the nicotine concentration in vapes and reducing time spent vaping (Sahr 2021). This study reported vaping cessation at six months and found greater quit rates in those using the reduction intervention; however, the 95% CI was wide and also incorporated the possibility of no intervention effect and higher quit rates in the minimal support study arm (RR 3.38, 95% CI 0.43 to 26.30; 17 participants; very low‐certainty evidence; Analysis 4.1). See Figure.

**5:**
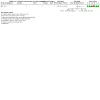
Nicotine/vaping reduction versus minimal support for nicotine vaping cessation

The numbers of participants reporting SAEs and change in combustible tobacco use at six‐month follow‐up or longer were not reported for this comparison.

###### Important outcomes

Sahr 2021 also measured nicotine vaping cessation at three‐month follow‐up. There were marginally higher quit rates in the minimal support group; however, the 95% CI was wide again (RR 0.96, 95% CI 0.57 to 1.64; 17 participants; Analysis 4.2).

The other important outcomes measured by Sahr 2021 were weight (lbs), systolic blood pressure (mm/Hg), and heart rate (bpm) at follow‐up. In all three analyses there was substantial imprecision, indicating potential increases, decreases, or no change in the intervention arm versus minimal support (weight: MD 13.12 lbs, 95% CI ‐26.99 to 53.23; 12 participants; Analysis 4.3; systolic blood pressure: 1.45 mmHg, 95% CI ‐10.01 to 12.91; 15 participants; Analysis 4.4; heart rate: MD ‐3.80, 95% CI ‐20.22 to 12.62; 15 participants; Analysis 4.5).

None of our other important outcomes were reported.

##### Text message‐based interventions

###### Critical outcomes

Graham 2021 and Graham 2024 (both judged at low risk of bias) compared the same text message‐based nicotine vaping intervention to no/minimal support controls and reported nicotine vaping cessation at seven‐month follow‐up. The pooled analysis resulted in an RR of 1.32 (95% CI 1.19 to 1.47; 4091 participants; low‐certainty evidence; Analysis 5.1), with no statistical heterogeneity detected (I^2^ = 0%). See Figure. Therefore, there was no detectable moderating effect (I^2^ = 0%) of subgrouping the studies by the age of participants recruited (under 18 years versus under and over 18 years). Both of the studies included in this analysis were funded by the Truth Initiative, which provided the text message‐based intervention, and both were carried out in young people; one in 13‐ to 17‐year‐olds (Graham 2024) and one in 18‐ to 24‐year‐olds (Graham 2021).

**6:**
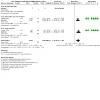
Text message‐based interventions versus no/minimal support for nicotine vaping cessation

Graham 2021 measured change in combustible tobacco use at six‐month follow‐up in two different ways. Firstly, they reported the number of people who were smoking tobacco at baseline and were abstinent from smoking at seven month follow‐up. This resulted in an RR of 1.03 (95% CI 0.90 to 1.19; 793 participants; very low‐certainty evidence; Analysis 5.2) and demonstrated no clear evidence of higher smoking quit rates in either group. Secondly, they reported the number of people who were not smoking tobacco at baseline, but were smoking at seven‐month follow‐up. Again, there was no clear evidence of a difference between groups (RR 1.04, 95% CI 0.81 to 1.33; 1036 participants; very low‐certainty evidence; Analysis 5.3).

Three studies reported the number of participants reporting SAEs for this comparison (Graham 2024; Klein 2024; NCT05140915); zero SAEs were reported in all studies across study arms (2082 participants) and therefore it was not possible to calculate a pooled effect (low‐certainty evidence; Analysis 5.4).

###### Important outcomes

Two studies (Klein 2024; NCT05140915), one judged to be at high risk of bias and one at unclear risk of bias, reported nicotine vaping cessation at between three and six months. Overall cessation rates were slightly higher in the text message‐based intervention; however, 95% CIs also incorporated the null and a potential benefit of control (RR 1.04, 95% CI 0.84 to 1.29; 576 participants; Analysis 5.5). No statistical heterogeneity was detected (I^2^ = 0%) and grouping based on the age of participants and removing the only study at high risk of bias (Klein 2024) had no impact on the interpretation of results.

Three studies involving 1953 participants in this comparison also reported the number of participants reporting AEs (Graham 2024; Klein 2024; NCT05140915). Graham 2024 (low risk of bias) and NCT05140915 (unclear risk of bias) did not report any AEs across their study arms, so it was not possible to pool studies and calculate an average effect. The individual result for Klein 2024 (high risk of bias) was an RR of 1.00 (95% CI 0.70 to 1.44; 379 participants; Analysis 5.6).

None of our other important outcomes were reported for this comparison.

##### Financial incentives

###### Critical outcomes

None of our critical outcomes were assessed for this comparison.

###### Important outcomes

One small study testing two intervention types alone and in combination investigated the effect of financial incentives versus no/minimal support (Michaud 2025; unclear risk of bias). The only outcome the study tested relevant to this review was nicotine vaping cessation at 12 weeks of follow‐up. The data indicated a higher chance of stopping vaping in the financial incentive conditions. However, there was substantial imprecision and the CI also incorporated the null and the potential for higher stopping rates with no/minimal support (RR 2.00, 95% CI 0.75 to 5.33; 30 participants; Analysis 6.1).

None of our other important outcomes were reported for this comparison.

##### Media literacy e‐learning

###### Critical outcomes

None of our critical outcomes were assessed for this comparison.

###### Important outcomes

Michaud 2025 (unclear risk of bias) was also the only study to investigate the effect of a vaping‐specific media literacy e‐learning intervention. More people in the no/minimal support conditions stopped vaping at 12 weeks of follow‐up than in the media literacy e‐learning conditions. However, the 95% CI also encompassed the possibility of no difference and increased stopping with the media literacy e‐learning intervention (RR 0.43, 95% CI 0.11 to 1.68; 30 participants; Analysis 7.1).

None of our other important outcomes were reported for this comparison.

#### Combination behavioural interventions versus no/minimal support

##### App‐based intervention + text messaging

One small study investigated the effects of an app‐based intervention combined with text message support versus no/minimal support (Heffner 2025; high risk of bias).

###### Critical outcomes

Heffner 2025 reported no SAEs across both trial arms (61 participants); therefore, it was not possible to calculate the RR and 95% CI (Analysis 8.1).

None of our other critical outcomes were assessed for this comparison.

###### Important outcomes

Heffner 2025 provided vaping cessation rates at three months follow‐up, with higher rates in the intervention and control arm. However, there was substantial imprecision, meaning that the 95% CI also incorporated a potential lack of effect and a greater effect in the no/minimal support arm (RR 6.78, 95% CI 0.37 to 125.95; 61 participants; Analysis 8.2).

Heffner 2025 also reported AEs and found a lower number of AEs in the app + text messaging arm; however, there was again substantial imprecision and thus uncertainty in the effect estimate (RR 0.32, 95% CI 0.04 to 2.93; 61 participants; Analysis 8.3).

None of our other important outcomes were reported for this comparison.

##### Media literacy e‐learning + financial incentives

###### Critical outcomes

None of our critical outcomes were assessed for this comparison.

###### Important outcomes

Michaud 2025 (unclear risk of bias) assessed the combined effect of media literacy e‐learning and financial incentives compared to no/minimal support. The only one of our important outcomes measured was vaping cessation at between three and six months. Michaud 2025 found slightly higher vaping cessation rates at 12 weeks in the no/minimal support arm. However, the result was very imprecise, also encompassing no effect and a beneficial intervention effect (RR 0.90, 95% CI 0.24 to 3.38; 19 participants; Analysis 9.1).

#### Combination pharmacotherapy and behavioural interventions versus no/minimal support

##### Combination NRT + print‐based self‐help materials

###### Critical outcomes

Palmer 2023, judged to be at high risk of bias, was the only study that compared combination NRT + print‐based self‐help to minimal support. Our only outcome reported by this study was the number of people reporting SAEs. None of the participants reported SAEs across both study arms, and so it was not possible to calculate an effect estimate (23 participants; Analysis 10.1). None of our other critical outcomes were reported.

###### Important outcomes

Palmer 2023 reported that 10 of 12 participants in the intervention arm (combination NRT + print‐based self‐help materials) reported AEs. However, they did not report the AE rate in the minimal support arm and so it was not possible to calculate a relative risk.

None of our other important outcomes were reported.

##### Combination NRT + text message‐based intervention

###### Critical outcomes

Klein 2024, judged to be at high risk of bias, was the only study eligible for this comparison. It reported the number of people reporting SAEs and found that zero participants reported SAEs in both study arms. This meant that it was not possible to calculate an effect estimate (256 participants; Analysis 11.1).

Our other critical outcomes were not reported.

###### Important outcomes

Klein 2024 also reported nicotine vaping cessation at three months follow‐up and the number of participants reporting AEs. More participants quit vaping in the combination NRT and text messaging intervention study arm; however, 95% CI also incorporated the possibility of no effect of the intervention and a benefit in the control arm (RR 1.27, 95% CI 0.93 to 1.72; 256 participants; Analysis 11.2). More adverse events were reported in the combination NRT + text message‐based intervention arm than the control arm, with the 95% CI excluding the null (RR 2.56, 95% CI 1.46 to 4.47; 196 participants; Analysis 11.3).

None of our other important outcomes were reported.

#### Head‐to‐head comparisons

##### Higher versus lower dose NRT

###### Critical outcomes

None of our critical outcomes were assessed for this comparison.

###### Important outcomes

Palmer 2025, judged to be at unclear risk of bias, compared three different NRT doses to one another in three separate study arms. All participants received 4 mg lozenges, plus the standard‐dose arm received 21 mg nicotine patches, the mid‐dose arm received 21 mg patches + 14 mg patches (35 mg total) and the high‐dose arm received two lots of 21 mg patches (42 mg total). Palmer 2025 assessed our AE outcome only. For both the high‐dose versus standard‐dose comparison (RR 1.19, 95% CI 0.68 to 2.08; 31 participants; Analysis 12.1) and the mid‐dose versus standard dose comparison (RR 1.54, 95% CI 0.96 to 2.48; 31 participants; Analysis 13.1) more participants reported AEs in the higher dose arms. However, in the former case, the 95% CI also incorporated the null and the potential for more people reporting AEs in the standard dose arm, and in the latter case, the 95% CI also incorporated the null. When comparing the high‐dose arm to the mid‐dose arm, more participants reported AEs in the mid‐dose arm. However, there was again imprecision, with the CIs incorporating the potential for no difference between arms and more participants reporting AEs in the high‐dose arm (RR 0.77, 95% CI 0.51 to 1.16; 30 participants; Analysis 14.1).

##### Combination NRT versus nicotine/vaping reduction

###### Critical outcomes

Sahr 2021 was the only eligible study to directly compare combination NRT to an intervention combining a reduction in both nicotine concentration and vaping frequency. This was a small study, judged to be at high risk of bias. Nicotine vaping cessation rates were slightly higher in the nicotine/vaping reduction arm; however, there was substantial imprecision and the 95% CI incorporated both benefit, harm, and no effect of either intervention (RR 0.76, 95% CI 0.17 to 3.33; 15 participants; Analysis 15.1). No further critical outcomes were reported by Sahr 2021.

###### Important outcomes

Sahr 2021 also measured nicotine vaping cessation at three‐month follow‐up. Again, cessation rates were higher in the nicotine/vaping reduction arm but the 95% CI also encompassed the null and a potential benefit of combination NRT (RR 0.57, 95% CI 0.22 to 1.47; 15 participants; Analysis 15.2).

The following important outcomes were also reported by Sahr 2021: weight (lbs), systolic blood pressure (mmHg), and heart rate (bpm) at follow‐up. For all of the outcomes, the 95% CIs were wide and incorporated potential increases, decreases, and no change in the combination NRT arm versus the nicotine/vaping reduction arm (weight: MD 20.80 lbs, 95% CI ‐27.49 to 69.09; 11 participants; Analysis 15.3; systolic blood pressure: MD 6.87 mmHg, 95% CI ‐8.67 to 22.41; 11 participants; Analysis 15.4; heart rate: MD ‐0.43 bpm, 95% CI ‐18.96 to 18.10; 11 participants; Analysis 15.5).

None of our other important outcomes were reported.

##### Combination NRT versus text message‐based intervention

###### Critical outcomes

Klein 2024, judged to be at high risk of bias, was the only eligible study that directly compared combination NRT with a text message‐based intervention. The only critical outcome reported was the number of participants reporting SAEs, with zero events reported across study arms. Therefore, it was not possible to calculate an effect estimate (252 participants; Analysis 16.1).

No further critical outcomes were reported.

###### Important outcomes

Klein 2024 also reported nicotine vaping cessation at three‐month follow‐up. Vaping cessation rates were higher in the combination NRT arm than the text messaging arm; however, the 95% CI also incorporated the null and a potential benefit of text messaging over combination NRT (RR 1.16, 95% CI 0.84 to 1.58; 252 participants; Analysis 16.2). The only other important outcome reported by Klein 2024 was the number of people reporting AEs. There was a higher rate of AEs reported in the combination NRT arm than the text messaging arm, with the 95% CI excluding the null (RR 2.99, 95% CI 1.57 to 5.70; 183 participants; Analysis 16.3).

None of our other important outcomes were reported.

##### Media literacy e‐learning versus financial incentives

###### Critical outcomes

None of our critical outcomes were reported.

###### Important outcomes

When directly comparing a vaping‐specific media literacy e‐learning intervention and a financial incentives intervention, Michaud 2025 (unclear risk of bias) found that more people quit vaping at 12 weeks follow‐up with the financial incentives intervention. However, the 95% CI also incorporated the possibility of no effect and higher quit rates in the media literacy e‐learning arm (RR 0.36, 95% CI 0.09 to 1.47; 31 participants; Analysis 17.1).

#### Associations between moderators of interest and vaping cessation

Three studies evaluated the impact of our moderators of interest on our vaping cessation outcome (Fucito 2024; Graham 2021; Rigotti 2024) (Table). While Fucito 2024 found evidence that vaping cessation rates were higher in those with a history of combustible tobacco use, Rigotti 2024 found no evidence of an association between the two. Fucito 2024 considered that this might be related to the fact that participants who smoked in the past were less likely to report near constant vaping prior to study entry than those who had not smoked in the past (15/21, 71.4% versus 18/19, 94.7% respectively). Rigotti 2024 also reported no evidence of an association between age and vaping cessation. Graham 2021 reported that higher vaping intensity (vaping within 30 minutes of waking) was inversely associated with vaping cessation.

**2:** Associations between moderators of interest and vaping cessation

**Study ID**	**Study design**	**Length of follow‐up (months)**	**Study size**	**Overall RoB judgement**	**Age**	**Vaping intensity**		**History of combustible tobacco use**	
Fucito 2024	RCT	3	40	Unclear				↑ *Participants who had used tobacco cigarettes. Vaping cessation: 10/21, 47.6%*	↓ *Participants who had not used tobacco cigarettes. Vaping cessation: 5/19, 26.3%*
Graham 2021	RCT	7	2588	Low		↓ *Higher‐intensity vaping. Lower vaping cessation rates: 22.6% (text message) vs 16.4% (control); P < 0.001*	↑ *Lower‐intensity vaping. Higher vaping cessation rates: 31.4% (text message) vs 28.6% (control); P = 0.51.*		
Rigotti 2024	RCT	4	160	Low	↔ No evidence of effect on cessation in intervention group			↔ No evidence of effect on cessation in intervention group	

KEY: Effect direction as reported by authors: upwards arrow ↑ = more participants quit vaping; downwards arrow ↓ = fewer participants quit vaping. ↔ = no observed association with quitting vaping. RCT: randomised controlled trial; RoB: risk of bias *Authors' judgements* *Fucito: “Adults with tobacco smoking histories were more likely to quit vapes at 3 months than those without (10/21, 47.6% vs 5/19, 26.3%), regardless of group.”* *Graham 2021: Quitting rates at 7 months were lower in both study arms in those who reported vaping within 30 minutes of waking (text message 22.6% vs control 16.4%; P < 0.001) than among those who reported vaping 30 minutes after waking (text message 31.4% vs control 28.6%; P = 0.51).* *Rigotti 2024: In the intervention (cytisinicline and behavioural support) arm “there was no evidence that the effect differed by subgroups defined by age, sex, race, history of cigarette smoking, e‐cigarette dependence, age of vaping initiation, or e‐liquid flavor used.”*

## Discussion

### Summary of main results

This updated review of interventions to aid nicotine vaping cessation includes 15 RCTs (six new to this update) identified in searches up to July 2025. These studies investigated a range of comparisons, looking at pharmacotherapies, behavioural approaches, and combinations of the two. The number of studies contributing to each comparison was minimal, limiting the conclusions that could be drawn due to serious imprecision in most cases.

Three pharmacotherapies were investigated: combination NRT; varenicline; and cytisine. Despite very limited data, there was low‐certainty evidence that varenicline may improve quit rates at long‐term follow‐up when compared to placebo. However, this finding should be treated with caution as it may change as more data become available. Many of the studies that reported SAEs reported zero or one event. This results in low‐certainty evidence due to imprecision, but the low absolute rates of SAEs in the included studies do not indicate concerns over the safety of the pharmacotherapies assessed. The pharmacotherapies tested here are licenced and considered safe when used for smoking cessation.

Four forms of behavioural support were tested in isolation: reducing nicotine concentration and time spent vaping; a vaping‐specific media literacy e‐learning intervention; financial incentives; and text message‐based interventions. In the case of the first three interventions, only one small study contributed data to each, and so conclusions could not be drawn. However, for text message‐based interventions, there was low‐certainty evidence that they may result in higher quit rates than no/minimal support in youths and young adults. There was very low‐certainty evidence that they did not affect smoking outcomes.

Some eligible studies investigated interventions combining multiple components, such as NRT in conjunction with print‐based self‐help and text messaging or a smartphone app combined with text messaging or media literacy e‐learning plus financial incentives. However, the findings were inconclusive due to the limited data available. This was also the case for comparisons between two active interventions, such as higher versus lower dose NRT, combination NRT versus text messaging and media literacy e‐learning versus financial incentives.

### Limitations of the evidence included in the review

We judged five of the included studies to be at low risk of bias, four at unclear risk, and six at high risk. We deemed four studies to be at high risk due to issues with blinding and two due to attrition. Of the 15 included studies, 14 were carried out in the USA and one in Italy; therefore, results may not be generalisable to other countries, particularly where the regulation and availability of vaping products may differ. The majority of the included studies were carried out in adults, with only one exclusively recruiting participants under 18 years of age. Of the 12 studies carried out in adults, five of these only included younger adults (aged 18 to 29 years). This is particularly relevant for our comparison of text message‐based interventions versus no/minimal support; the studies contributing to the low‐certainty evidence that text message‐based interventions may help people to stop vaping nicotine recruited only young people aged between 13 and 24 years. Therefore, it is not yet possible to conclude whether this finding extends into older age groups who may have higher levels of dependence on nicotine. Just over half of the studies appeared to recruit a mixture of people who had smoked combustible tobacco cigarettes and who had never smoked (although this was not always stated explicitly), with only two studies solely recruiting people who had previously smoked and one study explicitly stating that they only recruited people who had never smoked. This is relevant as there may be differences in the nicotine dependence levels of people with and without a history of tobacco smoking that could affect the likelihood of successfully quitting vaping. The evidence would benefit from studies clearly stating eligibility based on baseline combustible tobacco smoking status and history. In addition, other elements besides tobacco smoking may impact dependence levels, which in turn may impact intervention effects; future studies should be clear about the vaping/nicotine dependence measures and criteria used in their studies, and report whether effects differ based on these variables.

Four of the 15 studies included in the review were funded by the manufacturers of the intervention, one investigating varenicline (Caponnetto 2023), one cytisine (Rigotti 2024), and two the same text message‐based intervention (Graham 2021; Graham 2024). We intended to carry out sensitivity analyses for all outcomes, removing these studies to test their potential effects on our pooled outcomes. However, due to the limited number of studies included in each analysis, these were not informative.

We conducted GRADE ratings for our comparisons of individual pharmacotherapies and behavioural interventions when compared to placebo, minimal support, or no support, for our critical outcomes. When considering our vaping cessation outcome, we deemed the evidence of very low (for combination NRT and nicotine/vaping reduction) and low (for varenicline and text message‐based interventions) certainty. No studies reported vaping cessation at six months or longer for our cytisine comparison. We downgraded the evidence on vaping cessation for text message‐based interventions twice, to low certainty, due to indirectness. This was because the intervention being tested in both of the contributing studies was the same, and the intervention was only tested in young people. Therefore, we do not know if this finding would be generalisable to other text message‐based interventions and to older adults. One of these studies also reported on smoking cessation and smoking uptake; we considered these findings to have very low certainty due to the aforementioned issues with indirectness, as well as imprecision (as the CI incorporated the possibility of both benefit and harm). In all other instances where we judged the evidence to be of very low certainty, this was because the only study contributing to a comparison was judged to be at high risk of bias, plus there was serious imprecision. For the varenicline versus placebo comparison, where we judged the evidence to be of low certainty, this was because there was serious imprecision alone. For the combination NRT versus no/minimal support comparison, we deemed the evidence for change in combustible tobacco use at six months or longer to be of very low certainty. The single study for this comparison had a high risk of bias and the results were substantially imprecise; for both reasons, we downgraded the evidence twice. We also deemed the evidence on this outcome for text message‐based interventions to be of very low certainty, due to imprecision and indirectness. Again, for both reasons, we downgraded the evidence twice. For comparisons where our SAE outcome was measured by at least one study, we deemed the evidence to be of low certainty or very low certainty, either through downgrading by two levels due to imprecision in the former case or by also downgrading by one level due to risk of bias in the latter case. Few studies contributed to these analyses, the number of events was zero or very low and where the 95% CIs could be calculated, these encompassed the possibility of a benefit, harm, and no effect of the intervention.

As well as only two studies contributing to our critical outcome, change in the use of combustible tobacco use at six‐month follow‐up or longer, only one study reported combustible tobacco use at four‐month follow‐up. In all cases, the results were inconclusive. It is important that future studies measure this outcome, as encouraging people who have formerly smoked tobacco to stop vaping could lead to a relapse to combustible tobacco smoking. Additionally, encouraging people who have never smoked to stop vaping could encourage them to access nicotine elsewhere through tobacco smoking. Regardless of whether an intervention increases vaping quit rates, if it leads to greater smoking this would be a concern, as although vapes are unlikely to be risk‐free, evidence suggests they are considerably less harmful than combustible tobacco [22].

As the analyses conducted in this review contained small numbers of studies, we were unable to statistically assess publication bias. We made every effort to identify eligible unpublished studies, and we assessed the possibility of selective reporting for each study. We did not find any clear evidence of missing data relevant to the comparisons and outcomes in this review.

Overall, the evidence on interventions for quitting vaping demonstrates considerable uncertainty and more studies are needed to draw and strengthen conclusions. We identified 32 ongoing studies that may be eligible for inclusion in this review upon completion. This review will continue to be updated as a living review, with monthly searches and updates triggered as findings become available that could change our conclusions (see Supplementary material 7 for more information).

### Limitations of the review processes

We consider the methods used to carry out this systematic review to be robust. Our search strategy included a key topic‐specific conference abstract book and trial registries searched through CENTRAL, meaning we were able to capture a number of ongoing studies. However, it is possible that there are unpublished data our searches did not uncover.

For outcome assessment, we followed standard methods used for Cochrane Tobacco Addiction Group smoking cessation reviews and extended them to our vaping cessation outcomes. It is standard practice in smoking cessation research to consider participants lost to follow‐up as continuing to smoke. In this case, we assumed that participants vaping at baseline continued to vape if they were lost to follow‐up.

One of our review authors is an author of one of the included studies (NAR). This author was not involved in the decision about inclusion of their study, the risk of bias assessment for their study, or the GRADE assessments for outcomes that included their study. This approach is standard across all Cochrane reviews (regardless of subject area) and has been approved by the Cochrane editorial office as sufficient to avoid bias.

### Agreements and disagreements with other studies or reviews

This is an update of a Cochrane living systematic review [57]. For this updated version, we added six new studies, as we discovered studies investigating new comparisons and new relevant outcomes for existing comparisons. However, our conclusions have not changed as adding the new data did not change the interpretation of our findings.

A non‐Cochrane systematic review published in 2022 (with searches updated to September 2021) sought to evaluate the evidence related to the outcome of vaping cessation [21]. Seven of the studies identified investigated the outcome of an intervention for helping a person or people to quit vaping. Two of the studies were RCTs that were also included in this review (Graham 2021; Sahr 2021), one was a single‐arm intervention study, three were case studies, and another was a case series. The interventions tested were NRT, varenicline, a text message‐based intervention, nicotine/vaping reduction, and financial incentives. All of these interventions were included in this review. The authors concluded that due to a paucity of evidence (only one study, also included in this review, was adequately powered) there was little to no evidence for effective vaping cessation strategies. This was also concluded by a more recent review of the vaping cessation evidence conducted as part of a Royal College of Physicians' report looking at e‐cigarettes and harm reduction, published in 2024 [22]. The evidence review, carried out in 2023, found that there was little evidence on the best ways to support people to stop vaping, and that further studies were required. A more recent systematic review and meta‐analysis published in 2025, with searches up to date until January 2024, included seven RCTs [23]. Four of these studies are included in this review; two were excluded due to insufficient follow‐up length and one was excluded as the intervention did not aim to help people to quit vaping (although vaping cessation was measured). Different types of vaping cessation interventions were grouped together for the analyses and there was some evidence that vaping cessation interventions in general can increase vaping cessation rates.

A Cochrane review was published in 2023, investigating interventions to prevent or cease electronic cigarette (vape) use specifically in children and adolescents [79]. It did not find any eligible studies up to their search date of May 2023, although 22 ongoing studies were identified.

The pharmacological treatments investigated in this review are those with the strongest evidence for smoking cessation, i.e. cytisine, varenicline, and combination NRT [80]. This review has provided low‐certainty evidence that varenicline is also effective for quitting vaping, when compared to placebo. Clear evidence of a benefit of cytisine and combination NRT has not been demonstrated; however, this is likely (at least in part) due to a paucity of evidence, and in the case of all three pharmacotherapies, the incorporation of further data may change our conclusions.

A Cochrane review of behavioural interventions for smoking cessation found high‐certainty evidence of the effectiveness of financial incentives for smoking cessation [81]. There was also high‐certainty evidence and moderate‐certainty evidence that counselling and text message‐based interventions were effective, respectively [81]. The current review found moderate‐certainty evidence that text message‐based interventions may be effective for vaping cessation in young adults. There was very limited evidence available on the use of financial incentives for vaping cessation and, due to imprecision, it is not possible to draw conclusions on their use for vaping cessation. However, this would be a reasonable direction for further study.

## Authors' conclusions

### Implications for practice

- There is low‐certainty evidence (downgraded two levels due to indirectness) that a text message‐based intervention may increase nicotine vaping quit rates in youths and young adults (13 to 24 years old), in comparison to no or minimal support, seven months after intervention start. There is very low‐certainty evidence (downgraded two levels due to indirectness and two levels due to imprecision) that it does not affect smoking uptake or cessation.
- There is low‐certainty evidence (limited by imprecision) that varenicline may increase nicotine vaping quit rates versus placebo; however, further data may change this conclusion.
- Risk of bias and imprecision preclude conclusions regarding the effects of combination nicotine replacement therapy (NRT), cytisine, and a nicotine concentration and vaping behaviour reduction programme on nicotine vaping quit rates.
- There is very limited evidence looking at serious unintended consequences of pharmacotherapies or behavioural interventions for quitting nicotine vaping, making it difficult to draw conclusions on potential harms. Where these were measured, rates of serious adverse events (SAEs) were extremely low across arms. The pharmacological interventions tested (combination NRT, cytisine, and varenicline) are licenced for the purposes of quitting smoking globally and considered safe for that indication.
- There is very limited evidence on the effectiveness and potential harms of interventions combining behavioural support and pharmacotherapies, combining more than one behavioural component, and comparing relevant interventions head‐to‐head for vaping cessation.
- Results of one other study reporting whether nicotine vaping cessation interventions had an effect on the number of people smoking combustible tobacco cigarettes at six months or longer, and results of the one study measuring this at four months, were inconclusive.

### Implications for research

Further randomised controlled trials (RCTs) are needed investigating interventions to help people to stop vaping, with a follow‐up period of at least six months and as long as is feasible. The interventions tested so far largely reflect interventions that have been found to be effective for tobacco smoking cessation. Further studies should continue to investigate these approaches and potential others, including counselling, which are also deemed effective for smoking cessation. It would also be helpful if studies were conducted with a comparator arm where vaping cessation was not encouraged (i.e. no treatment provided) in order to assess the effect of providing vaping cessation interventions on people's tobacco smoking rates. As well as measuring rates of vaping cessation, studies should measure unintended harms of the interventions, including serious adverse events and the impact of the interventions on rates of combustible tobacco smoking.

RCTs should be adequately powered and have processes in place to counteract risks associated with blinding and attrition (for example, using placebo as a comparator where appropriate, balancing face‐to‐face contact between study arms, biochemically validating vaping and smoking status, and optimising follow‐up contact procedures).

It is possible that the effects of interventions may differ based on the dependence levels of intervention users, which could differ according to nicotine vaping and/or tobacco smoking history and frequency of vaping. Investigators should consider the range of people to whom vaping cessation interventions are relevant, based on both tobacco smoking and vaping history, and clearly specify the baseline characteristics of participants in terms of both of these characteristics. We found low‐certainty evidence that varenicline and a text message‐based intervention may help more people quit nicotine vaping than no or minimal support. In the latter case, further research is needed to see if these findings are relevant to older adults (as well as young people), and if they extend to other text message‐based interventions.

## Supporting Information

Supplementary materials are available with the online version of this article: 10.1002/14651858.CD016058.pub3.

Supplementary materials are published alongside the article and contain additional data and information that support or enhance the article. Supplementary materials may not be subject to the same editorial scrutiny as the content of the article and Cochrane has not copyedited, typeset or proofread these materials. The material in these sections has been supplied by the author(s) for publication under a Licence for Publication and the author(s) are solely responsible for the material. Cochrane accordingly gives no representations or warranties of any kind in relation to, and accepts no liability for any reliance on or use of, such material.

**Supplementary material 1** Search strategies

**Supplementary material 2** Characteristics of included studies

**Supplementary material 3** Characteristics of excluded studies

**Supplementary material 4** Characteristics of ongoing studies

**Supplementary material 5** Analyses

**Supplementary material 6** Data package

**Supplementary material 7** Justification and methods for 'Living Review' approach

**Supplementary material 8** Data to be extracted from included studies

## Figures and Tables

**Table:** 

**Date**	**Event**	**Description**
25 November 2025	New search has been performed	This is a Living Systematic Review. Searches are updated and screened monthly. Last search date 1 July 2025. Six new studies included.
25 November 2025	New citation required but conclusions have not changed	Main conclusions remain unchanged. Update of this Living Systematic Review was triggered as two new studies were identified in July 2025 investigating two new comparisons: smartphone app + text messaging; higher versus lower dose nicotine replacement therapy.

## Additional information

### Registration and protocol

Protocol DOI: 10.1002/14651858.CD016058. Cochrane approved the proposal for this review in April 2024.

For all updates to the protocol, see: https://doi.org/10.17605/OSF.IO/6AC9J.

### Data, code and other materials

As part of the published Cochrane review, the following are made available for users of the Cochrane Library: our search strategy Supplementary material 1; full citations for all included studies, all ongoing studies, and relevant excluded studies in the reference section of the review; study data, including study information, study arms, and risk of bias assessments in our characteristics of studies tables (Supplementary material 2; Supplementary material 3; Supplementary material 4); analysis data, including overall estimates, subgroup estimates, and individual data rows (all the rows in all the analyses), which are in the main review and in Supplementary material 6. Data supporting the results of this systematic review are from published information and are available in the review. Analyses were conducted with Cochrane’s authoring tool, RevMan, using the inbuilt computation methods; for details of the computational methods, see https://documentation.cochrane.org/revman-kb/statistical-methods-210600101.html. Data were extracted in Covidence and are available from the authors on reasonable request. The data are shared within the published review directly from RevMan.

### History

Protocol first published: Issue 5, 2024 Review first published: Issue 1, 2025
