# Supplementary material for: Interventions for quitting vaping
Source: Cochrane Database Syst Rev. 2025 Nov 25;2025(11):CD016058. doi: 10.1002/14651858.CD016058.pub3 (PMC12645533; doi:10.1002/14651858.CD016058.pub3)
Supplement: Supplementary file 1 — Supplementary material 1 Search strategies [file CD016058-SUP-01-searchStrategy.html]

Search strategies


# Supplementary material 1 to: Interventions for quitting vaping

Butler AR, Lindson N, Livingstone-Banks J, Notley C, Turner T, Rigotti NA, Fanshawe TR, Begh R, Wu AD, Brose L, Conde M, Simonavičius E, Hartmann-Boyce J
  
https://doi.org/10.1002/14651858.CD016058.pub3

The material in this section has been supplied by the author(s) for publication under a Licence for Publication and the author(s) are solely responsible for the material. Cochrane has peer reviewed this material in accordance with its editorial policies, but Cochrane has not copyedited, formatted or proofread. Cochrane accordingly gives no representations or warranties of any kind in relation to, and accepts no liability for any reliance on or use of, such material.

Back to top

# Search strategies

## Initial search strategies

### Ovid databases (MEDLINE, Embase, PsycINFO)

1. (e-cig\* or ecig\* or electr\* cigar\* or electronic nicotine).mp. or (vape or vapes or vaporizer or vapourizer or vaporiser or vapouriser or vaper or vapers or vaping).ti,ab. or exp Electronic Nicotine Delivery Systems/ or exp Vaping/

2. (randomized controlled trial or controlled clinical trial).pt. or randomized.ab. or placebo.ab. or clinical trials as topic.sh. or randomly.ab. or trial.ti.

3. exp animals/ not human/

4. 2 not 3

5. 1 and 4

6. exp Smoking Cessation/

7. "Tobacco-Use-Cessation"/

8. (nicotine dependence or tobacco dependence).mp.

9. exp Smoking/th

10. "Tobacco-Use-Disorder"/

11. Smoking reduction/

12. exp Pipe smoking/ or exp Tobacco smoking/ or exp Tobacco Products/

13. ((quit\* or stop\* or cessation or ceas\* or giv\* or abstain\* or abstinen\* or reduc\*) adj5 (e-cig\* or ecig\* or electr\* cigar\* or electronic nicotine or vape or vapes or vaporizer or vapourizer or vaporiser or vapouriser or vaper or vapers or vaping)).ti,ab.

14. exp Tobacco/ or exp Nicotine/

15. 6 or 7 or 8 or 9 or 10 or 11 or 12 or 13 or 14

16. 5 and 15

### CENTRAL (via CRS-Web)

1. (e-cig\* or ecig\* or electr\* cigar\* or electronic nicotine):TI,AB,KY,MH,EMT,KW,XKY,EH,KY

2. (vape or vapes or vaporizer or vapourizer or vaporiser or vapouriser or vaper or vapers or vaping):TI,AB,KY,MH,EMT,KW,XKY,EH,KY

3. MESH DESCRIPTOR Electronic Nicotine Delivery Systems EXPLODE ALL or MESH DESCRIPTOR Vaping EXPLODE ALL

4. (Quit\* or stop\* or cessation or ceas\* or giv\* or reduc\* or abstain\* or abstinen\*):TI,AB,KY,MH,EMT,KW,XKY,EH,KY

5. MESH DESCRIPTOR Smoking Cessation EXPLODE ALL or MESH DESCRIPTOR Tobacco Use Disorder EXPLODE ALL

6. #1 OR #2 OR #3

7. #4 OR #5

8. #6 AND #7

## Ongoing search strategies

### Ovid databases (MEDLINE, Embase, PsycINFO)

1. exp case control studies/ or exp cohort studies/ or Case control.tw. or (cohort adj (study or studies)).tw. or Cohort analy$.tw. or (Follow up adj (study or studies)).tw. or (observational adj (study or studies)).tw. or Longitudinal.tw.

2. (e-cig\* or ecig\* or electr\* cigar\* or electronic nicotine).mp. or (vape or vapes or vaporizer or vapourizer or vaporiser or vapouriser or vaper or vapers or vaping).ti,ab. or exp Electronic Nicotine Delivery Systems/

3. (randomized controlled trial or controlled clinical trial).pt. or randomized.ab. or placebo.ab. or clinical trials as topic.sh. or randomly.ab. or trial.ti.

4. exp animals/ not human/

5. 3 not 4

6. 2 and 5

7. 1 and 2

8. 6 or 7

9. smoking cessation.mp. or exp Smoking Cessation/

10. tobacco cessation.mp. or "Tobacco-Use-Cessation"/

11. (nicotine dependence or tobacco dependence).mp.

12. exp Smoking/th

13. "Tobacco-Use-Disorder"/

14. Smoking reduction/ or Smoking reduction.mp.

15. exp Pipe smoking/ or exp Tobacco smoking/ or exp Tobacco Products/

16. ((quit$ or stop$ or ceas$ or giv$ or abstain\* or abstinen\*) adj5 (smoking or smoke\* or tobacco)).ti,ab.

17. exp Tobacco/ or exp Nicotine/

18. 9 or 10 or 11 or 12 or 13 or 14 or 15 or 16 or 17

19. 8 and 18

### CENTRAL (via CRS-Web)

1. (e-cig\* or ecig\* or electr\* cigar\* or electronic nicotine):TI,AB,KY,MH,EMT,KW,XKY,EH,KY

2. (vape or vapes or vaporizer or vapourizer or vaporiser or vapouriser or vaper or vapers or vaping):TI,AB,KY,MH,EMT,KW,XKY,EH,KY

3. MESH DESCRIPTOR Electronic Nicotine Delivery Systems EXPLODE ALL or MESH DESCRIPTOR Vaping EXPLODE ALL

4. #1 OR #2 OR #3
