# Supplementary material for: Interventions for quitting vaping
Source: Cochrane Database Syst Rev. 2025 Nov 25;2025(11):CD016058. doi: 10.1002/14651858.CD016058.pub3 (PMC12645533; doi:10.1002/14651858.CD016058.pub3)
Supplement: Supplementary file 3 — Supplementary material 3 Characteristics of excluded studies [file CD016058-SUP-03-characteristicsOfExcludedStudies.html]

Characteristics of excluded studies


# Supplementary material 3 to: Interventions for quitting vaping

Butler AR, Lindson N, Livingstone-Banks J, Notley C, Turner T, Rigotti NA, Fanshawe TR, Begh R, Wu AD, Brose L, Conde M, Simonavičius E, Hartmann-Boyce J
  
https://doi.org/10.1002/14651858.CD016058.pub3

The material in this section has been supplied by the author(s) for publication under a Licence for Publication and the author(s) are solely responsible for the material. Cochrane has reviewed this material, but Cochrane has not copyedited, formatted or proofread. Cochrane accordingly gives no representations or warranties of any kind in relation to, and accepts no liability for any reliance on or use of, such material.

Back to top

# Characteristics of excluded studies

## Table of contents

- Studies ordered by Study ID
  - Chen 2025
  - Clausen 2023
  - Haug 2022
  - Jenssen 2021
  - Krishnan 2023
  - Meltzer 2017
  - NCT03670264
  - NCT04951193
  - NCT04982978
  - NCT05112562
  - NCT05906082
  - NCT05985538
  - NCT06129123
  - Orfin 2024
  - Palmer 2022
  - Tran 2025
  - Vickerman 2021
  - Webb 2023
- References to studies

## Studies ordered by Study ID

| Study | Reason for exclusion |
| --- | --- |
| Chen 2025 | Ineligible study design |
| Clausen 2023 | Ineligible outcomes. Less than 3 months. |
| Haug 2022 | Ineligible outcome. Abstinence from e-cigarettes and combustible cigarettes was combined. |
| Jenssen 2021 | Ineligible outcome |
| Krishnan 2023 | Ineligible outcomes. Four-week study. AEs not reported. |
| Meltzer 2017 | Ineligible comparator |
| NCT03670264 | Ineligible outcomes. Less than 3 months of follow-up. |
| NCT04951193 | Ineligible outcomes |
| NCT04982978 | Ineligible outcomes |
| NCT05112562 | Ineligible outcomes. Abstinence from e-cigarettes not measured at 3 and 4 months of follow-up. |
| NCT05906082 | Ineligible outcomes |
| NCT05985538 | Ineligible outcomes. Use of vaping measured at 4 weeks. Other relevant short-term outcomes not assessed. |
| NCT06129123 | Ineligible outcomes |
| Orfin 2024 | Ineligible study design. Single arm. |
| Palmer 2022 | Ineligible outcomes. Less than 3 months of follow up. |
| Tran 2025 | Ineligible outcomes. |
| Vickerman 2021 | Ineligible interventions |
| Webb 2023 | Ineligible outcomes. Short follow-up and no relevant short term outcomes |

## References to studies

### Chen 2025 {published data only}

- Chen Y, Veldhuizen S, Selby P, Zawertailo L. Is a traditional smoking cessation program effective for vaping cessation? Drug and Alcohol Dependence 2025;267(Supplement):111640. [DOI: 10.1016/j.drugalcdep.2024.111640]

### Clausen 2023 {published data only}

- Clausen B, Rinck M, Nizio P, Matoska CT, Zappi C, Smits JAJ et al. Study protocol for approach bias retraining for nicotine addiction among dual combustible and electronic cigarette users. Contemporary clinical trials 2023;128():107145 2023;128:107145. [DOI: 10.1016/j.cct.2023.107145]

### Haug 2022 {published data only}

- Haug S, Boumparis N, Wenger A, Schaub MP, Paz Castro R. Efficacy of a mobile app-based coaching program for addiction prevention among apprentices: a cluster-randomized controlled trial. International Journal of Environmental Research and Public Health 2022;19(13):15730. [DOI: 10.3390/ijerph192315730]
- Haug S, Castro RP, Wenger A, Schaub MP. Efficacy of a smartphone-based coaching program for addiction prevention among apprentices: study protocol of a cluster-randomised controlled trial. BMC Public Health 2020;20(1):1910. [DOI: 10.1186/s12889-020-09995-6]
- ISRCTN59908406. Efficacy of ready4life: a digital addiction prevention program for young people. https://trialsearch.who.int/Trial2.aspx?TrialID=ISRCTN59908406 2020.

### Jenssen 2021 {published data only}

- Jenssen BP, Hannan C, Kelly MK, Ylioja T, Schnoll RA, Fiks AG. Response to: more detail needed for pilot e-cigarette treatment trial in youth and young adults. Nicotine & Tobacco Research 2021;23(11):1983-4. [DOI: DOI: 10.1093/ntr/ntab103]
- Jenssen BP; Hannan C, Kelly MK, Ylioja T, Schnoll RA, Fiks AG. Inability to recruit adolescents for a vaping cessation clinical trial within a large pediatric health system. Nicotine & Tobacco Research 2021;23(9):1633-4. [DOI: DOI: 10.1093/ntr/ntab034]

### Krishnan 2023 {published data only}

- Krishnan N, Berg CJ, Le D, Ahluwalia J, Graham AL,  Abroms LC. A pilot randomized controlled trial of automated and counselor-delivered text messages for e-cigarette cessation. Tobacco Prevention & Cessation 2023;9:04. [DOI: 10.18332/tpc/157598]

### Meltzer 2017 {published data only}

- \*Meltzer LR, Simmons VN, Sutton SK,  Drobes DJ, Quinn GP, Meade CD et al. A randomized controlled trial of a smoking cessation self-help intervention for dual users of tobacco cigarettes and E-cigarettes: Intervention development and research design. Contemporary clinical trials / 2017;60(101242342):56-62 2017;60:56-62. [DOI: 10.1016/j.cct.2017.06.014]
- NCT02416011. Smoking cessation self-help for dual users of tobacco cigarettes and e-cigarettes. https://clinicaltrials.gov/show/NCT02416011.

### NCT03670264 {published data only}

- NCT03670264. Be smokefree: behavioral economics incentives to engage adolescents in smoking cessation. https://clinicaltrials.gov/show/nct03670264 2018;(first accessed 13 May 2024).

### NCT04951193 {published data only}

- \*NCT04951193. Goal2quitvaping for nicotine vaping cessation among adolescents. https://clinicaltrials.gov/show/NCT04951193 (first received June 27 June 2024).
- Dahne J, Wahlquist AE, Levins O, Natale N, Carpenter MJ, Squeglia LM. Feasibility trial of vapex: a digital health intervention to promote vaping cessation among adolescents with depressive symptoms. In: SRNT 30th Annual Meeting Edinburgh March. Vol. PPS18-1. 2024.

### NCT04982978 {published data only}

- NCT04982978. Information interventions to reduce vaping in a student population. https://clinicaltrials.gov/show/NCT04982978 2021 2021;(accessed 22 July 2024).

### NCT05112562 {published data only}

- NCT05112562. Attentional bias modification training for e-cigarette users. https://clinicaltrials.gov/show/NCT05112562 2021;(accessed 22 July 2024).

### NCT05906082 {published data only}

- NCT05906082. Vape-free text-messaging: pilot study. https://clinicaltrials.gov/ct2/show/NCT05906082 (accessed 22 July 2024).

### NCT05985538 {published data only}

- NCT05985538. Impact of e-cigarette prevention messages on adolescents. https://clinicaltrials.gov/ct2/show/NCT05985538 (accessed 22 July 2024).

### NCT06129123 {published data only}

- NCT06129123. An online intervention to reduce e-cigarette use and susceptibility to smoking in young adults. https://clinicaltrials.gov/ct2/show/NCT06129123 2023;(accessed 22 July 2024).

### Orfin 2024 {published data only}

- Orfin RH, Ramos Santiago JW, Decena Soriano R, Romero Acosta E, Bermudez D, Rodriguez YL et al. Kick Vaping: feasibility, acceptability, and preliminary impact of a vaping cessation text messaging intervention for Latino young adults. Journal of Ethnicity in Substance Abuse 2024;Aug:1-13. [DOI: 10.1080/15332640.2024.2397422]

### Palmer 2022 {published data only}

- Palmer AM, Tomko RL, Squeglia LM, Gray KM, Carpenter MJ, Smith TT et al. A pilot feasibility study of a behavioral intervention for nicotine vaping cessation among young adults delivered via telehealth. Drug and Alcohol Dependence 2022;232(7513587):109311. [DOI: 10.1016/j.drugalcdep.2022.109311]

### Tran 2025 {published data only}

- Tran DD, Davis, Jordan P, Buch K, Leventhal AM, Ewing SWF et al. A novel online vaping intervention and smoking prevention program for young adults who vape: protocol for a randomized controlled trial. Addiction Science & Clinical Practice 2025;20(1):36. [DOI: 10.1186/s13722-025-00566-x]

### Vickerman 2021 {published data only}

- Vickerman KA, Carpenter KM, Miles LN, Hsu JM, Watt KA, Brandon TH et al. Treatment development, implementation, and participant baseline characteristics: A randomized pilot study of a tailored quitline intervention for individuals who smoke and vape. Contemporary Clinical Trials Communications 2021;24(101671157):100845. [DOI: 10.1016/j.conctc.2021.100845]

### Webb 2023 {published data only}

- Webb J, Lin Y-T, Ang A, Michero D, Majeed A, Eisingerich A. Feasibility and preliminary outcomes of a mobile intervention combining cognitive behavioral therapy, virtual coaching, and nicotine replacement therapy for nicotine vaping cessation. Telemedicine Reports 2023;4(1):48-52. [DOI: DOI: 10.1089/tmr.2023.0009]
