# Supplementary material for: Interventions for quitting vaping
Source: Cochrane Database Syst Rev. 2025 Nov 25;2025(11):CD016058. doi: 10.1002/14651858.CD016058.pub3 (PMC12645533; doi:10.1002/14651858.CD016058.pub3)
Supplement: Supplementary file 4 — Supplementary material 4 Characteristics of ongoing studies [file CD016058-SUP-04-characteristicsOfOngoingStudies.html]

Characteristics of ongoing studies


# Supplementary material 4 to: Interventions for quitting vaping

Butler AR, Lindson N, Livingstone-Banks J, Notley C, Turner T, Rigotti NA, Fanshawe TR, Begh R, Wu AD, Brose L, Conde M, Simonavičius E, Hartmann-Boyce J
  
https://doi.org/10.1002/14651858.CD016058.pub3

The material in this section has been supplied by the author(s) for publication under a Licence for Publication and the author(s) are solely responsible for the material. Cochrane has reviewed this material, but Cochrane has not copyedited, formatted or proofread. Cochrane accordingly gives no representations or warranties of any kind in relation to, and accepts no liability for any reliance on or use of, such material.

Back to top

# Characteristics of ongoing studies

## Table of contents

- Studies ordered by Study ID
  - ACTRN12623000022662
  - ACTRN12625000143426
  - Barnes 2025
  - Borrelli 2025
  - Chadi 2023
  - Champion 2025
  - CTIS2023-504708-27-00
  - CTIS2023-505036-35-00
  - Evans 2025
  - KCT0010346
  - Krishnan-Sarin 2024
  - Lyu 2022
  - McColgan 2024
  - NCT04146714
  - NCT04898075
  - NCT05892445
  - NCT05936099
  - NCT05994209
  - NCT06027840
  - NCT06142877
  - NCT06164678
  - NCT06196489
  - NCT06395415
  - NCT06662305
  - NCT06765291
  - NCT06832098
  - NCT06862050
  - NCT06885606
  - NCT06909500
  - NCT06929520
  - Sanchez 2023
  - TCTR20250203006
- References to studies

## Studies ordered by Study ID

ACTRN12623000022662

| Study name | The OurFutures Vaping Program: A cluster randomised controlled trial to evaluate the efficacy of a school-based eHealth intervention to prevent e-cigarette use among adolescents |
| Methods | RCT, parallel group.  11-15 year olds (<18)  Setting: School  Country: Australia  Study aim: The OurFutures Vaping Program is a universal school-based eHealth prevention program that aims to prevent the uptake, and reduce the use, of e-cigarettes among adolescents. The program is built on the effective “OurFutures” (formerly “Climate Schools”) prevention model which is based on social influence and social competence principles.  Blinding: Participating secondary schools will be randomly allocated to one of two groups: i) an active control group (usual health education) or ii) an intervention group (the OurFutures Vaping Program). |
| Participants | Target enrolment 3360  Inclusion criteria: Eligible participants will be all Year 7 and/or Year 8 students attending participating schools in 2023. Students will be required to be fluent in English, provide informed active consent, and only students who receive parental consent will be eligible to participate.  Exclusion criteria: Schools with fewer than 70 enrolled Year 7/8 students in 2023. -Schools based outside NSW, WA and QLD. |
| Interventions | Intervention: OurFutures Vaping Program. eHealth prevention program to prevent uptake, and reduce use, of e-cigarettes among adolescents. Year 7/8 health education classes. The program consists of 4x40-minute lessons (delivered one week apart over 4 weeks) consisting of a web-based cartoon component completed individually by students (approx. 20mins), followed by optional teacher-facilitated activities (e.g., quizzes, class discussions, role plays). Factsheets (designed specifically for this study) are provided after each lesson to summarise and reinforce key content.  Control: Active control group (usual health education) |
| Outcomes | Baseline, post-test (post-completion of 4-week intervention), 6-, 12-, 24- and 36-month follow-up.  Primary: Uptake of e-cigarette use. To assess this, students will be asked “Have you ever used a vape, even one or two puffs?” (Yes/No). The primary endpoint will be the 12-month follow-up.  Secondary: Intentions to use tobacco cigarettes in the next year; Frequency of e-cigarette use; Quantity of e-cigarette use (articipants who report vaping within the past 30 days are asked how how many sessions a day they vape); Anxiety symptoms (PROMIS Anxiety Paediatric Item Bank); Attitudes towards e-cigarettes; E-cigarette refusal skill techniques; Psychological distress (Kessler 6); Frequency of tobacco cigarette use; Quality of life (Child Health Utlity); Externalising symptoms (Strengths and Difficulties Questionnaire); Depressive symptoms. adolescent version of the Patient Health Questionnaire-8; Self-efficacy to resist peer pressure. Assessed using an adapted version of the Resistive Self-Regulatory Efficacy Scale; Internalising symptoms (Strengths and Difficulties Questionnaire); Quantity of tobacco cigarette use; Knowledge about e-cigarettes and tobacco cigarettes (assessed as a composite outcome); Motives to use e-cigarettes (adapted version of the Tobacco Motives Inventory); Intentions to use e-cigarettes in the next year (single item based on those used in our previous school-based trials); Resource utilisation (self-report resource use questionnaire); Wellbeing (Short Warwick–Edinburgh Mental Well-being Scale); Perceptions of Stress (Perceived Stress Scale); Uptake of tobacco cigarette use. Assessed using a single item: “Have you ever tried smoking a cigarette, even one or two puffs?” (Yes/No). |
| Starting date | Date first enrollment: 24/03/2023 |
| Contact information | Lauren Gardner, The University of Sydney, Level 6, Jane Foss Russell building (G02) University of Sydney NSW 2006 Australia.  lauren.gardner@sydney.edu.au |
| Notes | Funding source: Medical Research Future Fund (MRFF). |

ACTRN12625000143426

| Study name | Public title: Replication trial of the OurFutures Vaping Program: Evaluation of a school-based eHealth program to prevent e-cigarette use among adolescents in NSW Public Schools  Scientific title: Replication trial of the OurFutures Vaping Program: A cluster randomised controlled trial of a school-based eHealth intervention to prevent e-cigarette use among adolescents in NSW Public Schools. |
| Methods | RCT (cluster randomised controlled trial)  Masking: Open (masking not used);Assignment: Parallel. |
| Participants | Target size: 1265  Inclusion criteria: 11-15 years old, Year 7 and/or Year 8 students attending participating NSW Public schools in 2025. Fluent in English, provide informed active consent, only students who receive parental consent will be eligible to participate. Exclusion criteria: -Schools with fewer than 70 enrolled Year 7/8 students in 2025. Schools based outside NSW. Schools that are not public/government schools. |
| Interventions | The OurFutures Vaping Program is a universal school-based eHealth prevention program that aims to prevent the uptake, and reduce the use, of e-cigarettes among adolescents. The program is built on the effective “OurFutures” (formerly “Climate Schools”) prevention model which is based on social influence and social competence principles.  Participating secondary schools will be randomly allocated to one of two groups:  i) an active control group (usual health education) or  ii) an intervention group (the OurFutures Vaping Program).  The OurFutures Vaping Program aligns with the Australian and state-based Health & Physical Education Curriculums and is designed to be delivered during Year 7/8 health education classes. The program consists of 4x40-minute lessons (delivered one week apart over 4 weeks) consisting of a web-based cartoon component completed individually by students (approx. 20mins), followed by optional teacher-facilitated activities (e.g., quizzes, class discussions, role plays). There are quizzes and reflective activities embedded in the cartoons to ensure student engagement, comprehension, and critical thinking. Factsheets are provided after each lesson to summarise and reinforce key content.   The intervention aims to provide students with evidence-based information about e-cigarettes and tobacco cigarettes (e.g., what they are made of, short- and long-term harms, the influence of media and marketing, signs of nicotine addiction, coping and help-seeking information and the benefits of avoiding e-cigarettes and tobacco cigarettes), to modify existing norms, and improve resistance skills (via practicing assertive communication and other refusal skills).   Students and teachers access the intervention materials online via the OurFutures V |
| Outcomes | Measured at baseline, post-test (post-completion of 4-week intervention) and 12-month follow-up. The primary timepoint is 12-months.]  Primary outcome: Past 12-month e-cigarette use.  Secondary outcomes: Quantity of e-cigarette use. Frequency of e-cigarette use.  Frequency of tobacco cigarette use. Quantity of tobacco cigarette use. Uptake of tobacco cigarette use.  Motives to use e-cigarettes. Intentions to use tobacco cigarettes in the next year. Psychological distress. Internalising symptoms. Knowledge related to e-cigarettes, tobacco cigarettes and related harms. Self-efficacy to resist peer pressure. Attitudes towards e-cigarettes. Intentions to use e-cigarettes in the next year |
| Starting date | Date first enrollment: 04/03/2025 |
| Contact information | Lauren Gardner, lauren.gardner@sydney.edu.au  University of Sydney |
| Notes | Funding: National Health and Medical Research Council  Added to second update (2025) |

Barnes 2025

| Study name | Efficacy of a text-message based intervention in preventing adolescent e-cigarette use  Setting: Australia |
| Methods | RCT 2x2 factorial design |
| Participants | Adolescents aged 12 to 15 years.  Aim: 120  Recruited as part of a larger factorial trial (ACTRN12623000079640). Baseline: 40 adolescents and 41 parents. At 6 months: 30 adolescents and 35 parents  Recruitment: a number of channels including social media advertising, school newsletters, advertisements on community noticeboards. Parents who had participated in previous research studies conducted by the research team were also contacted.  Eligibility: (1) parents and adolescents must have sufficient English proficiency to engage with the intervention and (2) adolescents must own or have exclusive access to a mobile phone. Adolescents will not be excluded if they report ever e-cigarette and/or cigarette use; however, they will be analysed separately. |
| Interventions | Parent-adolescent dyads will be randomly allocated to:  1. Adolescent text-messages  2. Parent text-messages  3. Parent and adolescent text-messages  4. Control information only control (EC factsheet provided to parents only). |
| Outcomes | Baseline, 6, 12 and 24 months  Collect EC use (secondary outcome) and CC use. |
| Starting date | March 2023 |
| Contact information | Dr Courtney Barnes; Courtney.Barnes@health.nsw.gov.au  Hunter New England Population Health, Hunter New England Local Health District, Wallsend, New South Wales, Australia |

Borrelli 2025

| Study name | Virtual reality for the prevention and cessation of nicotine vaping in youth: Protocol for a randomized controlled trial. |
| Methods | RCT |
| Participants | 150 (98 intervention and 52 control)  13-19 years |
| Interventions | Intervention: Virtual Reality Program, Virtual Reality vaping cessation and prevention program.  The VR Program will be pre-installed on Meta Quest 2 headsets, approximately the length of one school class period (approximately 30-40 minutes). Each participant will experience the program once a week for 3 weeks. Participants who missed a week will complete the program by the 4th or 5th week.  VR program participants will be able to download a mobile app that complements and reinforces the school-based VR session.  Control: Assessment Only. Students in classrooms randomized to this group will not receive the VR intervention but will complete questionnaire assessment only. |
| Outcomes | 5 weeks  Past 30-days vaping frequency; Past 7-days vaping frequency; Frequency of current vaping; Motivation to quit (or avoid) vaping within the next 30-days; Motivation to quit (or avoid) vaping |
| Starting date | Approved April 2, 2024, and data collection began in April 2024 and concluded in June 2024, |
| Contact information | Belinda Borrelli belindab@bu.edu |

Chadi 2023

| Study name | A brief digital screening and intervention tool for parental and adolescent tobacco and electronic cigarette use in pediatric medical care in Canada: protocol for a pilot randomized controlled trial (canCEASE) |
| Methods | RCT.  Single-center, pragmatic, single-blind, pilot RCT comparing the CEASE intervention to usual care (control condition).  Recruitment: various clinics within the Sainte-Justine University Hospital Centre, (Montreal, Quebec, Canada), including the general pediatrics clinic, the adolescent medicine clinic, and the orthopedics and sports medicine clinics.  Setting: Tertiary care paediatric hospital in Montreal, Quebec, Canada  Country: Canada  Study aim: To demonstrate the feasibility and evaluate the preliminary effectiveness of the CEASE program for parental smoking cessation and its adapted version for adolescent smoking cessation and adolescent and parental vaping cessation. |
| Participants | 130  Age group: Both under and over 18. Parents or guardians of children aged between 0 and 17 years, and adolescent patients aged between 14 and 17 year.  Participants may be dual users of vapes and tobacco cigarettes.  Inclusion: Parents or legal guardians of children 0-17 years old and who smoke or use nicotine vaping products. "User" will be defined as those who answer "Yes" to the screening questions: "Have you smoked a single cigarette, even a puff, in the past 7 days?" (smoking); "Have you used an e-cigarette or vaping device containing nicotine, even a puff, in the past 7 days? (vaping). For CEASE-A, adolescent patients aged 14-17 years who smoke or use nicotine vaping products will be considered. "Adolescent User" will be defined by those who answer "Yes" to the same screening question as parents. Parents will be eligible if 1) they are at least 18 years old, 2) their child is 0-17 years of age, 3) are attending a regular scheduled medical appointment, and 4) are sufficiently proficient in either French or English (able to read and answer a written questionnaire). Adolescents will be eligible if they are 14-17 years-old, meet criteria 3) and 4) above, and have provided informed consent (in Québec, adolescents aged 14 years can provide consent). Adolescents whose parent(s) (if present) are not agreeable to their participation will be excluded.  Exclusion: Families presenting to the clinic without a scheduled medical appointment will be excluded. There will be no other exclusion criteria. |
| Interventions | CEASE Intervention arm, behavioural intervention. CEASE (for parents) and CEASE-A (for adolescents) are evidence-based tobacco and vaping cessation interventions delivered in pediatric practices. CEASE and CEASE-A are based on the 5A's model of smoking cessation: Ask about smoking, Advise to quit, Assess readiness to quit, Assist with a quit plan and Arrange follow-up. Given that CEASE and CEASE-A are one-time interventions, "Arrange" is removed, and the fourth step "Assist" is divided into two parts: a) providing phone/text/app quit support and b) providing NRT.  Control usual care. The control condition will be care as is usually delivered in participating clinics with the possibility of receiving direct linkage with cessation services delivered via CEASE/CEASE-A at the end of the 6-month study period. Current practice does not include routine provision of assistance for parental/adolescent smoking or e-cigarette cessation (e.g., referral to quitlines, NRT prescription). |
| Outcomes | Baseline, 1, 3 and 6 months  Vaping abstinence and combustible cigarette abstinence at 6 months (7-day, self-reported and cotinine confirmed ).  Recruitment rates; feasibilty; at 12 mths. Retention at 6 months.  Secondary outcomes: attempts to quit; intent to quit at 6 months. |
| Starting date | Satrt dtate: 22 Feb 2023  Completion date: 10 June 2024 |
| Contact information | Nicholas Chadi, Sainte-Justine University Hospital Research Centre, Montreal, QC, QC, Canada.  o.drouin@umontreal.ca (Olivier Drouin) |
| Notes | Funding: This study is supported by a bridge grant from the Canadian Institutes of Health Research (CIHR). OD and NC are supported by Clinician Scholar Awards from the Fonds de recherche du Québec-Santé. MPS is supported by a Junior 2 Clinician Scholar Award from the Fonds de recherche du Québec-Santé. |

Champion 2025

| Study name | Optimising a digital intervention to support parents experiencing socio-economic disadvantage to improve adolescent health behaviours: protocol for the Health4Life Parents & Teens factorial trial |
| Methods | 24 factorial trial  All parents will receive six online modules.  This is a follow-up study of ACTRN12619000431123 (The Health4Life Study). The current trial aims to develop a parent intervention to accompany the school-based Health4Life intervention evaluated in ACTRN12619000431123. |
| Participants | 389 parents of adolescents in Australia. Families experiencing socio-economic disadvantage. |
| Interventions | Digital intervention for families experiencing socio-economic disadvantage  Intervention: All parents will receive six online modules (screen time, smoking/vaping, alcohol, sleep, food/nutrition, physical activity). Parents will be randomised to receive 1 of 16 combinations of additional components: text messages, tailored feedback, stress management, and health coaching. Parents will complete surveys at baseline and 3-months post-baseline.  Comparator: "Because of the MOST study design, there is no comparator/control treatment as such. Instead, the main effects of, and interactions between, intervention components will be determined." |
| Outcomes | Baseline, 3 months  Parent's e-cigarette use (vaping). Participant reported frequency of e-cigarette smoking in the past 6 months. Baseline and 3-month follow-up  Parent's alcohol use (frequency).  Parent's cigarette smoking |
| Starting date | Date submitted: 2/12/2024  Date first enrollment: 23/04/2025  Estimated last data collection date: 31/10/2025 |
| Contact information | Dr Katrina Champion, katrina.champion@sydney.edu.au |
| Notes | Added to second update (2025) |

CTIS2023-504708-27-00

| Study name | Cytisine for nicotine-containing electronic cigarette and tobacco cigarette cessation: a randomized placebo-controlled trial |
| Methods | RCT |
| Participants | Target: 600  18-65  Inclusion  Exclusion: Pregnancy/breastfeeding, currently use any smoking cessation medication, including the nicotine-containing products and cytisine within at screening, a known hypersensitivity to cytisine or to any of the excipients, hospitalization for any of the following medical conditions in the previous 3 months: myocardial infarct/severe angina, stroke, severe arrhythmia or another severe heart-related condition, self-report diagnosis of pheochromocytoma, heart failure (IV NYHA), untreated active peptic ulcer/gastroesophageal reflux disorder, moderate/severe renal insufficiency, epilepsy or untreated hyperthyroidism, uncontrolled hypertension (systolic blood pressure > 160 mmHg, diastolic blood pressure > 100 mmHg), diagnosis of any non-treated and unstable psychotic disorders, including schizophrenia. |
| Interventions | Cytisine |
| Starting date | Date first enrollment: 02/11/2024 |
| Contact information | Prof. Piotr Tutka, ptutka@ur.edu.pl University of Rzeszow |
| Notes | Added to second update. |

CTIS2023-505036-35-00

| Study name | A study of nicotine freshmint mouthspray in e-cigarette users willing to quit |
| Methods | RCT. A multicenter, parallel group, double-blind, placebo-controlled, randomized.  Interventional clinical trial of medicinal product |
| Participants | Aim: 800  Eligibilty: daily nicotine intake from vaping for at least 3 months prior to screening visit; Want to become nicotine free with ‘high’ to ‘very high’ motivation based on the screening motivation assessment., Are classified as either moderately or highly dependent on the Penn State Electronic Cigarette Dependence Index (PS-ECDI), which corresponds to a total score of 9 or greater., Are exclusive e-cigarette users. No pregnancy or spouse pregant.  Exclusion criteria: 1. Use of other forms of tobacco / nicotine-containing product(s) other than EC within 7 days before screening; marijuana for 28 days prior to screening; clinically significant severe, acute or chronic, medical or psychiatric condition(s). Use of other forms of tobacco / nicotine-containing product(s) other than EC between the screening and baseline visits; Use of other smoking cessation medications or aid(s) within 28 days before screening (e.g. NRT, buproprion, varenicline, cytisine, counselling, apps, acupuncture, or hypnosis). |
| Interventions | 1. Nicorette Mint Spray 1 mg/Sprühstoß Spray zur Anwendung in der Mundhöhle, Lösung, Product Code:PRD2156181, Pharmaceutical Form: OROMUCOSAL SPRAY, SOLUTION,  2. Control: Placebo mint mouthspray (identical in appearance and formulation, except it does not contain nicotine). |
| Outcomes | Weeks 2, 4 and 6, 30, and 52  Continuous self-reported abstinence from EC and CO-verified abstinence from conventional cigarettes from the visit at week 2 up to and including the visits at weeks 6, 30, and 52 respectively, with cotinine verification at visits weeks 30 and 52.  7-day point-prevalence abstinence from EC. To assess the efficacy of Nicotine Freshmint Mouthspray vs placebo on craving and withdrawal symptoms, To document the compliance with the trial products., To document other tobacco / nicotine-containing product status and vaping status throughout the trial., To evaluate the safety of the trial products.  Safety will be monitored and assessed by reviewing the collection, evaluation, and analysis of participant-reported adverse events.  Separate urge-to-vape and urge-to-smoke severity in the last 24 hours recorded daily in a participant electronic diary (eDiary) from baseline up to the week 2 visit. E-cigarette consumption recorded daily in the eDiary from screening up to the week 2 visit.  Average daily number of trial product doses recorded in the CRFs at all post-baseline visits up to and including the visit at week 26.  EC consumption recorded in the CRFs at all post-baseline visits.  Point-prevalence 7-day abstinence from e-cigarettes at all post-baseline visits with COverification for smoking abstinence at all visits except for visits at weeks 8, 16, and 20 and with cotinine verification at visits weeks 30 and 52.  Continuous self-reported abstinence from e-cigarettes from the visit at week 2 up to and including the visits at weeks 4, 8, 12, 16, 20, and 26, respectively, with CO-verification of smoking abstinence at visits weeks 2, 4, 12, and 26.  Separate severity ratings of urge-to-vape and urge-to-smoke in the last 24 hours recorded in the CRFs at weeks 2, 4 and 6 visits. Severity ratings of individual and composite withdrawal symptom scores in the last 24 hours recorded in the CRFs at visits weeks 2, 4 and 6. |
| Starting date | First enrollment: 25/04/2024 |
| Contact information | McNeil AB, RA-JX2-ClinicalRegul@kenvue.com |
| Notes | Added to second update. |

Evans 2025

| Study name | Digital media for cancer control (to prevent vaping and smoking behavior)  Official title: Digital Media for Cancer Control: Randomized Controlled Trial and Dose-Response Effects |
| Methods | RCT. Factorial assignment. |
| Participants | Actual enrollment: 2800 NCT record  Evans 2025: 8437 participants, stratified by vaper (n=5026) and nonvaper (n=3321) status  Inclusion: Young adults age 18-24 who are members of the Ipsos/Knowledge Panel |
| Interventions | Digital intervention: Anti-vaping and anti-smoking digital advertising. Delivery of social media based advertising to prevent vaping and smoking among young adults ages 18-24.  Control: No intervention. No exposure control condition |
| Outcomes | Year 1, year 5.  Frequency of vaping during the past 30 days |
| Starting date | Study completion: 2024-10-05 |
| Contact information | Sponsor: George Washington University  Collaborator: National Institutes of Health (NIH)  Evans 2025. Funding This research was funded by the National Cancer Institute (grant CA253013). |
| Notes | New ongoing added to 2025 update. |

KCT0010346

| Study name | Digital interventions for smoking cessation among young adults in Korea: a randomized controlled trial |
| Methods | RCT |
| Participants | 19-39  Aim: 60  Inclusion criteria: This study targets adults aged 19 to 39 who are current smokers of either conventional cigarettes or EC, can receive face-to-face smoking cessation counseling at the Seoul Smoking Cessation Support Center, and agree to participate in the study after understanding its purpose.  Exclusion criteria: individuals who have previously received smoking cessation counseling at the Seoul Tobacco Cessation Center and those currently using NRT (e.g., nicotine patches, nicotine gum, or Nicotinell lozenges) or pharmacological smoking cessation treatments (e.g., varenicline, bupropion). |
| Interventions | Intervention: Digital-based smoking cessation messages. Intervention group receives standard smoking cessation counseling (at least three face-to-face sessions at the Seoul Tobacco Control Center), two brief text messages for smoking cessation verification, and additional digital-based smoking cessation interventions (text messages, photo messages, and short-form videos) three times per week.  Control group receives standard smoking cessation counseling (at least three face-to-face sessions at the Seoul Tobacco Control Center) and two brief text messages for smoking cessation verification. |
| Outcomes | Smoking cessation rate. |
| Starting date | Date of registration 2025-03-28 |
| Contact information | Jisoo Chae, 52, Ewhayeodae-gil, Seodaemun-gu, Seoul, jisoochae@ewha.ac.kr Ewha Womans University |
| Notes | New ongoing added to 2025 update. Funding: Ministry of Health & Welfare |

Krishnan-Sarin 2024

| Study name | Examining the use of a virtual youth-focussed, e-cigarette cessation intervention combining in-person CBT with abstinence-contingent incentives |
| Methods | Virtual RCT  Setting: virtual  Country: USA  Recruitment: recruited from local schools in Conneticut and online social media (Snapchat, Instagram, Facebook, TikTok) |
| Participants | 109 participant (99 at FU)  47 M, 50 F, 2 non-binary; M=17.6 years old; using e-cigarettes on 6.8 + 0.5 days/week, with a baseline urine cotinine level of M=1348.2+1075.1 ng/mL.  Youth (aged 13-20) who wanted to quit using e-cigarettes and used e-cigarettes regularly (at least 4 days/week; urine cotinine levels > 200 ng/ml) |
| Interventions | 6 week virtual cessation trial.  Randomised to receive incentives that were contingent (salivary cotinine < 30ng/ml) or non-contingent (providing salivary samples) on abstinence.  All participants received 6 remote weekly CBT sessions which started one week prior to their quit date. CBT content was derived from a youth-focused CBT manual and provided by therapists who were trained and supervised weekly. Starting on quit day participants also used an online program (NuRelm, Inc.) to provide salivary cotinine tests every other day for the 1st 2 weeks, then 2x per week for the final 2 weeks, and were randomized to receive incentives that were contingent (salivary cotinine < 30ng/ml) or non-contingent (providing salivary samples) on abstinence. |
| Outcomes | Baseline, 1,3,6, 12 months.  Primary outcome 7-day point-prevalence abstinence at the end of treatment (EOT at six weeks of treatment) based on self-reports and verified biochemically (saliva cotinine < 30 ng/ml).  Biochemically confirmed self-reports of abstinence at EOT (6 weeks) are at 37%. |
| Starting date | Not stated. |
| Contact information | Suchitra Krishnan-Sarin, University School of Medicine, New Haven, CT, USA. |
| Notes | Funding: Nonprofit grant funding entity. American Heart Association ENACT grant 20YVNR35460041 |

Lyu 2022

| Study name | Delivering vaping cessation interventions to adolescents and young adults on Instagram: protocol for a randomized controlled trial |
| Methods | Design: RCT, parallel assignment. Masking single (outcomes assessor)  Setting: Online (Instagram)  Country: USA  Recruitment: Adolescents and young adults aged 13–21 residing in California who have vaped at least once per week in the past 30 days will be recruited online via Facebook and other social media, augmented by outreach through community partners and youth serving organizations. |
| Participants | 500  Intervention group 250; Control group 250.  Inclusion: 1. English literacy;2. Age between 13-21 years; 3. Indicate they use social media “most” (≥4) days per week; 4. Have vaped at least once per week in the past 30 days; 5. Access to a computer or mobile phone with photo capability to verify abstinence from vaping; 6. considering quitting / interested in quitting within the next 6 months; 7. Reside in California. This is because the funder for this study, the California Tobacco Related Diseases Research Program, requires the research be conducted in California.  Exclusion: 1. No English literacy; 2. Age under 13 or over 21 years; 3. Insufficient social media use (3 or fewer days per week); 4. Have not vaped at least once per week in past 30 days; 5. No access to computer or mobile phone with photo capability to verify abstinence from vaping; 6. Not interested in or considering quitting within the next 6 months; 7. Not California residents. |
| Interventions | Social media intervention, Instagram with up to 3 posts per day for 30 days. Participants will be educated about signs of nicotine dependence and if they express interest in pharmacotherapy will be encouraged to access this through their personal healthcare providers. The Instagram groups will provide educational and social support, troubleshooting and advice about nicotine replacement therapy (NRT) or other forms of treatment.  Control Condition: no intervention. Directed to the Truth Initiative e-cigarette texting quit program. This innovative and free text message program was created with input from teens, college students and young adults who have attempted to, or successfully, quit e-cigarettes using text coaching methods |
| Outcomes | Baseline, 1, 3, 6 months  Point Prevalent Abstinence (PPA) from vaping.  Abstinence from all tobacco products.  Change in the number of participants: with reduction in vaping (by 50% or more); number of vape quit attempts. Change in response on: Stages of Change Questionnaire; Thoughts About Abstinence (TAA) Questionnaire; scores on the Ways of Quitting questionnaire (WOQ).  SRNT some data presented as percentage. Not extractable. |
| Starting date | 18 November 2021  18 June 2024 |
| Contact information | Pamela Ling, MD,University of California, San Francisco |
| Notes | Funding: California Tobacco Related Diseases Research Program. |

McColgan 2024

| Study name | Challenges and strategies for recruitment and retention of exclusive e-cigarette users in clinical trials |
| Methods | RCT  Phase 3 study to compare nicotine mouth spray and placebo over 1 year for abstinence.  Country: Canada, UK, Germany. |
| Participants | 800  Inclusion criteria: exclusive e-cigarette users |
| Interventions | Nicotine mouth spray vs placebo.  Remote visits, behavioral support, and a motivational assessment at screening to encourage subject retention, and the recruitment strategy utilizes AI-powered targeted recruitment through social media platforms most popular with the target demographic. |
| Outcomes | Study length 12 months.  Vaping abstinence |
| Starting date | Ongoing |
| Contact information | Bryan John McColgan, Kenvue, Helsingborg, Sweden, |

NCT04146714

| Study name | Substance use screening to encourage behavior change among young people in primary care  Official title: Screening for Excessive Substance Use in the Waiting Room to Encourage Behavior Change Among Young People (YP-HEALTH): a Multi-center Randomized Controlled Trial in Primary Care |
| Methods | RCT, parallel-group randomized controlled trial  Setting: primary care  Country: Switzerland  Recruitment: primary care practices in the French-speaking part of Switzerland.  Aim: to evaluates whether completing a short screening questionnaire about health behaviours in the waiting room before a primary care consultation decreases excessive substance use in young people aged 14 to 24 years. |
| Participants | 840 (estimated) (approximately 20 per practice)  Both over 18 and under 18 (young people: 14-24 years old).  Inclusion criteria: Patients aged 14 to 24 years consulting at the participating primary care practice for any motive.  Exclusion Criteria: Acute illness requiring immediate attention of the physician; Severe mental health conditions requiring treatment in a specialized setting; Young person not consulting as a patient at the practice (e.g. accompanying friend or partner); Inability to read the trial information in French or to provide independent consent. |
| Interventions | Two different types of confidential pre-consultation screening surveys: one focusing on the assessment of binge drinking and other substance use (intervention group) and the other on physical activity (control group).Substance use questionnaire vs Physical activity questionnaire  Intervention group: Screening questionnaire about substance use based on the Detection of Alcohol and Drug Problems in Adolescents (DEP-ADO) survey.  Control group: Screening questionnaire about physical activity, based on the short version of the International Physical Activity Questionnaire (IPAQ). |
| Outcomes | Baseline and follow-up phone interviews at 3, 6 and 12 months.  Outcome at 3 months follow-up proportion of patients reporting binge drinking (≥1 episode) in the past 30 days.  Secondary outcomes will include the proportion of young people reporting smoking (≥ 1 cigarette a day), electronic cigarette use (≥ once a day) and/or excessive cannabis use (≥1 joint/week) in the past 30 days. Analysis will be by intention to treat and will take into account clustering of participants within practices. |
| Starting date | Estimated start date: 2025-01  Estimated study completion: 2027-12 |
| Contact information | Dagmar M Haller  dagmar.haller-hester@unige.ch |

NCT04898075

| Study name | Quit nicotine: e-cig cessation intervention |
| Methods | Design: RCT  Country: USA  Setting: High school |
| Participants | N=100 (estimated)  Intervention group (CM for nicotine abstinence + CBT) n=50  Control group (CBT) n=50)  13-19 (both under and over 18 years)  Inclusion: High school students between ages 13-20 years. Regular e-cigarette (vape) user (primary EC users with or without other tobacco use who report using EC at least 4 days/week and have urine cotinine levels ≥200 ng/ml).  Exclusion: Substance Use Dependence (SUD) on other psychoactive substances. Current diagnosis of psychosis. Current diagnosis of a significant mental health disorder that is not being treated. |
| Interventions | Intervention: 4-week long web-based, remote Contingency Management (CM) for nicotine abstinence plus weekly individualized Cognitive Behavioral Therapy (CBT). Participants will be paid increasing amounts of payment for each negative saliva cotinine test.  Control. Participants will be paid for providing saliva nicotine test, regardless of whether the test is positive or negative.  Both groups will receive weekly CBT during this time (2 sessions prior to quitting, 4 weekly sessions after quitting). |
| Outcomes | Baseline, 1, 3, 6, and 12 months.  Vaping cessation  Self reported of no EC usage during the 7 days prior and confirmed negativity with cotinine levels of 30ng/mL (Alere iScreen OFD Cotinine Saliva Test; Countrywide Testing). |
| Starting date | April 2021. Estimated completion January 2025 |
| Contact information | Suchitra Krishnan-Sarin, Yale University. |
| Notes | Study collaborator: American Heart Association. |

NCT05892445

| Study name | Impact of aversive warnings on e-cigarette cessation intentions and behaviors among young adults |
| Methods | RCT  Country: USA  Recruitment: market research firms |
| Participants | N=1000 (estimated)  18-29 year old, using vapes at least once per week. |
| Interventions | Intervention: Aversive visual health warnings about the potential health risks of e-cigarette use, delivered through the online survey platform  Control group will not receive any intervention and will complete the same survey as the intervention group |
| Outcomes | 3 months  Follow-up assessments at 3-months post-intervention to investigate impact of aversive visual health warnings on e-cigarette cessation among young adults, including the moderating effects of prior adverse event experience. |
| Starting date | Start date May 2025 |
| Contact information | Raphael Cuomo, University of California, San Diego |

NCT05936099

| Study name | Adolescent inpatient tobacco and ENDS intervention  Official title: Development and implementation of a tobacco and ENDS use intervention for adolescents and young adults in the pediatric hospital |
| Methods | RCT |
| Participants | Age: 14-21  Estimated: 144. 96 Intervention. 48 control  Inclusion criteria: Admitted to the hospital. Screens positive for past 30 day e-cigarette use. Parent/guardian agrees to leave the room.  Exclusion criteria: Age is less than 14 years or over 21 years. Not comfortable speaking/reading English. Too ill to participate. Severe psychiatric illness. Too developmentally delayed/cognitively impaired. |
| Interventions | Randomise 2:1  Intervention: Behavioral: E-cigarette & Tobacco Use Treatment Intervention. The behavioral intervention includes health education on vaping health risk and outcomes, motivational interviewing, and assisted quit planning from a health educator and counseling and NRT (if appropriate) provided by a physician.  Control: No Intervention. Participants will complete the baseline survey and receive an informational brochure |
| Outcomes | Baseline, 3 months  3 month self-reported 30-day abstinence. Cessation verified with biochemical verification of a saliva sample.  Acceptability, feasibility and fidelity of intervention. |
| Starting date | Start date: 2023-08-01. Estim study completion: 2025-12-20 |
| Contact information | Abbey Masonbrink, MD, MPH,Children's Hospital Los Angeles  Sponsor: Children's Mercy Hospital Kansas City  Collaborators: National Institutes of Health (NIH). National Institute on Drug Abuse (NIDA) |
| Notes | New ongoing to 2025 update. |

NCT05994209

| Study name | Testing the feasibility and acceptability of social media and digital therapeutics to decrease vaping behaviors |
| Methods | RCT  Country: USA |
| Participants | N=189 (estimated)  Current vaper product user (P30D). A desire to quit vaping and/or experiencing negative health outcomes due to vaping.  Vaping product user only (i.e., not using vaping as a means to support combustible smoking cessation) |
| Interventions | Intervention Group A: quitSTART Mobile App Intervention. quitSTART  Experimental Group B: quitSTARTMobile App Intervention PLUS Embedded Chatbot Feature  Control group: No Intervention. (Nationwide resource referral and intervention waitlist) |
| Outcomes | Baseline, 6 weeks, 3 months.  Vaping cessation. 7 day self-reported vaping abstinence at follow-up.  Nicotine dependence will be assessed using the 4-item Patient-Reported Outcomes Measurement Information System (PROMIS).  Risk perception related to vaping.  Intervention engagement |
| Starting date | Jan 2024 (estimated). Completion estimated March 2025. |
| Contact information | Patricia Cavazos-Rehg, pcavazos@wust.edu  Washington University School of Medicine |

NCT06027840

| Study name | Concurrent vs. sequential cessation of dual cigarette and e-cigarette use |
| Methods | Randomized parallel assignment  Country: USA |
| Participants | N=40 (estimated)  18 years and older  Inclusion: Report cigarette smoking, and e-cigarette use for at least the past 3 months. Smoke 5 or more cigarettes per day. Report e-cigarette use for at least 14 days in the past month. Interested in quitting both products in the next month and willing to set a quit date.  Exclusion: use of other tobacco or nicotine products besides cigarettes and e-cigarettes > once per week in the last 30 days. |
| Interventions | Varenicline 12 weeks + counselling + booklet  This study examines whether concurrent treatment for cigarettes and e-cigarettes in which an individual quits both products at the same time (QUIT-C) or sequential treatment in which an individual quits cigarettes first followed by e-cigarettes is more effective for quitting both products.  Arm 1 Experimental: QUIT-C (Concurrent). Treatment in this arm will emphasize CONCURRENT cessation of cigarettes and e-cigarettes. All participants will receive 12-weeks of varenicline, weekly individual counseling, and access to cessation resources including a guided self-change booklet and links to free text-based support. Counseling and cessation resources will emphasize CONCURRENT cessation.  Arm 2: Experimental: QUIT-S (Sequential). Treatment in this arm will focus on cessation of cigarettes FOLLOWED SEQUENTIALLY by cessation of e-cigarettes. All participants will receive 12-weeks of varenicline, weekly individual counseling, and access to cessation resources including a guided self-change booklet and links to free text-based support. Counseling and cessation resources will emphasize SEQUENTIAL cessation. |
| Outcomes | 3 months  E-Cigarette abstinence. 7-day point prevalence e-cigarette abstinence at Week 12, biochemically verified by urine cotinine  Cigarette abstinence, 7-day point prevalence cigarette abstinence at Week 12, biochemically verified by breath carbon monoxide.  Biomarkers of tobacco-related harm exposure. Primary urinary biomarkers of tobacco-related harm exposure will be assessed including NNAL (4-(methylnitrosamino)-1-(3-pyridyl)-1-butanol), a tobacco carcinogen linked to lung cancer risk. |
| Starting date | April 2024. Estimated completion date June 2025. |
| Contact information | Lisa Fucito, Yale University, lisa.fucito@yale.edu |

NCT06142877

| Study name | Effects of social media use on young adults' e-cigarette use |
| Methods | RCT  Country: USA |
| Participants | N=200 (estimated)  Age 18-25; daily social media use; smartphone; nicotine vaping on 1-19 days of the past 30 days |
| Interventions | Experimental: Social Media Use Reduction. Participants will be incentivized to reduce their social media use by a pre-specified percentage from baseline.  No Intervention: Social Media Use as Usual |
| Outcomes | Baseline, 1, 3, and 6 months.  Number of days the participant vaped nicotine in the past 30 days.  Episodes per vaping day. Puffs per vaping episode |
| Starting date | December 2023. Estimated completion March 2025 |
| Contact information | Erin A Vogel, erin-vogel@ouhsc.edu |

NCT06164678

| Study name | Vaping cessation using the Ottawa model for smoking cessation among e-cigarette users |
| Methods | RCT  Parallel assignment  Country: Canada |
| Participants | N=180 (estimated)  18 years or over; vaping at least once per week for the past four weeks. |
| Interventions | Arm 1. Experimental: Ottawa Model for Smoking Cessation  The OMSC group will receive counselling and NRT if they choose with follow-up calls to support medication titration. Participants will be provided with quit cards, which are pre-loaded with $300 worth of funds that can only be used by the assigned study participant to purchase NRT (if they choose).  For those in the intervention group, the study counsellor who is a trained Nicotine Addiction Treatment Specialist (NATS) will facilitate follow-up, monitor NRT use, and advise participants to titrate NRT dose as required based on their minimum daily nicotine intake. These counselling calls will be conducted at day 3, 7, 14, 21, 30, 60, 90, and 180 as is standard in OMSC for people who smoke and are interested in quitting. A diary will also be provided to the participants to track their usage.  Arm 2. Control: Usual Care. The usual care group will receive the initial counselling session but no further follow-up or NRT. They will be able to self-initiate a follow-up call if they choose. Participants will not be excluded if they choose to initiate NRT on their own at their own expense. |
| Outcomes | Baseline, 3, 6, 12 months  Vaping cessation (7 day point prevalence), baseline to 3 months.  Continuous abstinence rates at 1-day, 7-day, 1-month, 3-month, 6-month, and 12-month follow up.  Self-reported cessation will be measured by a self-administered salivary cotinine test. 12 month assessment. |
| Starting date | July 2024. Estimated completion December 2025 |
| Contact information | Evyanne Quirouette, equirouette@ottawaheart.ca  Nia Patel, niapatel@ottawaheart.ca  Hassan Mir, Ottawa Heart Institute Research Corporation |
| Notes | Sponsor: Ottawa Heart Institute Research Corporation |

NCT06196489

| Study name | Adapting an intervention for vaping in young veterans |
| Methods | Randomised  Country: USA |
| Participants | N=20  Inclusion: young adult veterans (18-30 years old); daily e-cigarette user for at least the past 6 months; willing to enroll in a program to quit nicotine use within 30 days; smartphone/computer/tablet.  Exclusion: current use of combustible tobacco products at least weekly.  Population: Young veterans |
| Interventions | Arm 1. Experimental: receive the adapted vaping cessation intervention by telephone.  Arm 2. Experimental: will receive the adapted vaping cessation intervention by video telehealth. |
| Outcomes | Baseline, 2 months, 3 month follow-up  Number of days using e-cigarettes in the past 7 days at the time of assessment  Number of e-cigarette uses per day.  Client Satisfaction Questionnaire (CSQ-8) |
| Starting date | January 2024. Estimated study completion June 2024. |
| Contact information | Neal M Doran, neal.doran@va.gov, Veterans Medical Research Foundation.  Collaborator University of California |

NCT06395415

| Study name | Development of a mobile health intervention for electronic cigarette use among young adults (EQUIP)  Country: USA |
| Methods | RCT |
| Participants | Estimated enrollment: 46  Inclusion criteria: Age 18-26 years old. Current ENDS user (at least 4 out of 7 days per week of use for the past one month); for focus groups, at least half of participants will report current cigarette smoking (at least 1 cigarette per day on at least 1 day per week in the past month). Report interest in quitting or reducing EC (at least 6/10 scale in interest of quitting or reducing. Does not currently (in past one year) meet criteria for major psychiatric disorder including severe alcohol or substance use disorder (excluding tobacco use disorder), schizophrenia, bipolar disorder, and obsessive compulsive disorder. Not pregnant/breastfeeding. Not using smoking cessation medication (i.e., Varenicline, Bupropion. No history of adverse reactions to nicotine replacement therapy  Exclusion: No mobile phone. No interest in quitting. EC use <4 days per week. Psychiatric illness. Using smoking cessation medication. |
| Interventions | 1. Intervention: mobile health (mHealth) Participants in this arm will receive a 6-week mHealth message based intervention through their smartphone. Insight® platform. They will also have the option of requesting free nicotine replacement therapy (NRT) throughout the treatment period. 2 stage intervention. 1) Motivational enhancement (weeks 1-2) and 2) Skills and information (weeks 3-6). The first stage of messages will utilize personalized feedback and motivational interviewing prompts, while the second stage will provide health information on ENDS and skills, such as deep breathing. Content is tailored to motivation level (low/medium or high) and dual use status.  2. Standard care. Participants in this arm will receive a 5-10 minute walk-through of electronic nicotine delivery system (ENDS) and tobacco education and skills based on the most recent evidence. They will also have the options of requesting a sample of NRT. |
| Outcomes | Baseline, 6 weeks, 12 weeks  EC abstinence. ENDS cessation will be measured using a modified Timeline Followback Interview using 7-day point prevalence abstinence with study staff and then confirmed via a mailed saliva cotinine test.  EC use. Frequency of ENDS use as determined by Timeline Followback Interview with study staff will be measured as a secondary outcome. |
| Starting date | Start date: 2025-04-25. Estimated completion date: 2026-02 |
| Contact information | Emma Brett, PhD, ebrett@bsd.uchicago.edu  Sponsor: University of Chicago |
| Notes | New ongoing added to 2025 update |

NCT06662305

| Study name | Kick-Nic! Youth Quit Vaping App. Quit smoking study for people who use e-cigarettes  Yale University, USA |
| Methods | RCT |
| Participants | Estimated: 306  Inclusion criteria: 13-19 years old; regular current (past month) EC users using nicotine-containing EC at least 1 day/week; want to quit using EC; smartphone with data/WIFI plan to access and utilize the KickNic! app.  Exclusion criteria: non-stable (<2 months) use of anxiolytics, antidepressants, and other psychostimulants or history of psychosis or any significant current psychiatric/medical condition that would increase risk will be excluded. |
| Interventions | Intervention: Behavioral: Kick-Nic! mobile phone application. The Kick-Nic! app has seven modules that cover core CBT strategies using skills, graphic illustrations, and interactive components to help adolescents quit using e-cigarettes.  Control (active comparator): referral to the NCI Quit Vaping website. The NCI webpage provides quitting tobacco resources for youth, including for e-cigarettes. The information provided is similar to the CBT skills modeled by the Kick-Nic! app. |
| Outcomes | Baseline, 8 weeks, 3 months, 6 months  Follow-up visits will be conducted 1, 2, 3, and 6 months after the 8 week treatment period.  Vaping abstinence. (Self-report) 7-day point prevalence of EC use confirmed by saliva cotinine <30ng/mL.  Percentage days EC free and continuous abstinence of EC use at 8 weeks, 3 month, and 6 month follow up. |
| Starting date | 202.5-01-07. Estimated study completion: 2029-08-31 |
| Contact information | Thomas Liss. thomas.liss@yale.edu  Sponsor: Yale University |
| Notes | New ongoing study added to 2025 update. |

NCT06765291

| Study name | Behavioral intervention for youth to promote vaping cessation |
| Methods | RCT  Massachusetts General Hospital, USA |
| Participants | Estimated enrollment: 400  Inclusion critera: Age 14-18 inclusive. Self-report of at least weekly nicotine vaping for the prior ≥3 months and semiquantitative saliva screening for cotinine positive for recent nicotine use. Self-report of no regular combusted tobacco use (5 consecutive days of smoked tobacco use) in the 2 months prior to enrollment and exhaled CO <10 ppm. Report willingness to try to quit vaping in the next 30 days.  Exclusion criteria: Use of a smoking cessation medication in the prior month (nicotine patch, gum, nasal spray, or inhaler, varenicline, bupropion). Unwilling to abstain during the study from using smoking cessation aids other than those provided by the study. Unwilling to provide saliva or urine samples. |
| Interventions | 1. QuitVaping intervention plus texting support. Participants assigned to the QuitVaping intervention will receive referral to TIQ texting app and 12 brief, weekly, study intervention visits with a 36-week follow up.  2. EUC only. TIQ texting app referral to support vaping cessation. |
| Outcomes | Continuous 4-week nicotine vaping abstinence at end of treatm (weeks 9 to 12)  Continuous continine-verified nicotine vaping abstinence at the end of follow up (Weeks 9-36)  Percentage self-reporting nicotine vaping abstinence since the last visit on the timeline followback interview and have cotinine <10ng/mL, assessed at study weeks 9-12 and weeks 9-36. |
| Starting date | Estimated start date: 2025-03. Estimated completion date: 2028-11 |
| Contact information | A. Eden Evins, MD, MPH. aeevins@mgh.harvard.edu  Julia Jashinski, MSW. jjashinski@mgh.harvard.edu |
| Notes | New ongoing added to 2025 update. |

NCT06832098

| Study name | Nicotine patch plus nicotine mouth spray versus nicotine reduction for vaping cessation.  Official title: The New Zealand Quit Vaping Trial: Combination nicotine replacement therapy versus nicotine reduction. |
| Methods | RCT. A single-blind, two-arm, pragmatic community-based randomised trial.  Country: New Zealand  Recruitment throughout New Zealand, using multi-media advertising with targeted promotion to reach indigenous Māori, Pacific, low socio-economic groups, and people with disabilities, given their disproportionately higher prevalence of vaping.  Study aim: To evaluate the effectiveness, safety and acceptability of combination NRT plus behavioural support compared with vape nicotine tapering plus behavioural support, on six-month vaping abstinence. |
| Participants | Estimated enrollment 774 (387 per group).  Inclusion criteria: live in New Zealand, vape nicotine at least weekly, used to smoke tobacco regularly but not in the past 6-months OR have never smoked tobacco, are aged ≥16 years, and have no contraindications to the study treatment. Participants must be motivated to quit-vaping in the next eight weeks, have access to the internet, and be able to provide consent.  Exclusion criteria: another person in their household currently enrolled in the study; smoke tobacco currently or were a recent regular user of tobacco; enrolled in another vaping cessation programme/trial; self-report having had a serious cardiovascular event, or hospitalisation for a cardiovascular complaint, in the previous four weeks (e.g. stroke, myocardial infarction, unstable angina, cardiac arrhythmia, coronary artery bypass graft and angioplasty); self-report uncontrolled hypertension; strong preference to use/not use NRT or nicotine tapering; current users of smoking cessation pharmacotherapy. |
| Interventions | 1. Intervention: Combination NRT plus written behavioral support. 8 weeks of combination NRT (21mg patch plus 1mg mouth spray). Transdermal HABITROL® patches (21mg/24 hrs) plus NICORETTE® QUICKMIST mouth spray (1mg nicotine/spray dose). Other Names: HABITROL patches; NICORETTE QUICKMIST. advised to start using the patches daily, with the mouth spray used to relieve any 'breakthrough' cravings. Advice on reducing use of both products over the 8 week period will also be provided, following the manufacturers guidelines when these products are used for smoking cessation. Participants will also receive written behavioural support.  2. Comparator: 8 week Nicotine Tapering Plan plus written behavioral support. Tapering plan based on their current e-juice nicotine concentration and frequency of vaping upon entry to the trial. Participants will use their own vapes and nicotine e-liquid. Participants will also receive written behavioural support. |
| Outcomes | Vape-free and tobacco-free: self-reported continuous abstinence from vaping at six-months post-end of treatment. Self-report of no device use (defined as not more than five vaping sessions since end of treatment), and self-report of not smoking (defined as no use of any tobacco, but individuals may or may not be using NRT. The tobacco-free status will be verified using exhaled carbon-monoxide (CO) measurement with a Bedfont Smokerlyzer (≤5 ppm signifying abstinence).  Other outcomes at baseline and 6 mths: SAE; vape type; strength; flavour; fequency; vaping other substances; cannabis use; alcohol use; body mass index; COPD; coughing; shortness of breath; vape-free; vape and tobacco-free; nicotine-free; tobacco cigarettes per day; continuation of study product; |
| Starting date | 2025-03-25. Estimated completion date 2027-04-30 |
| Contact information | Amanda Calder, PhD. amanda.calder@auckland.ac.nz  Chris Bullen, PhD, MBChB. c.bullen@auckland.ac.nz  Current responsible party: George Laking, PhD, MBChB, University of Auckland, New Zealand  Original responsible party: Natalie Walker, University of Auckland, New Zealand  Collaborators: University College, London. Medical University of South Carolina. Flinders University |
| Notes | New ongoing study added to 2025 update. |

NCT06862050

| Study name | BREATHE free: a pilot feasibility trial. |
| Methods | RCT  Country: Augusta, Georgia, USA.  Study aim: to assess the feasibility and acceptability of an in-person faith-based intervention to help people stop tobacco use. The study will address use of different types of tobacco products, such as smoking combustible cigarettes, cigarillos and little filtered cigars, and using/vaping electronic nicotine delivery systems (vape pens, e-cigs, and JUUL). |
| Participants | Estimated: 90  Inclusion Criteria: Daily use of one or more tobacco products  Exclusion Criteria: Current use of tobacco cessation medications; Enrolled in tobacco treatment program |
| Interventions | Intervention: BREATHE Free Curriculum. Curriculum will teach practices: love, perspective, prudence, hope, humility, forgiveness, spirituality, self-control, teamworking, perseverance, gratitude, and joy. Attend 12 in-person weekly meetings and go through Breathe Free curriculum.  Control: This group will serve as a control group and will receive usual care, referral to tobacco quit line. |
| Outcomes | Baseline and weeks 12 and 16  Number of cigarettes (or cigars) smoked per day (CPD).  Smoking abstinence, biochemically verified point prevalence abstinence (7 days).  Expired Carbon Monoxide (CO) will be assessed in all participants. |
| Starting date | Estimated study start date: 2025-03-01. Estimated study completioj date: 2026-12-25 |
| Contact information | Ban Majeed, Augusta University, bmajeed@augusta.edu |
| Notes | New ongoing added to 2025 update. |

NCT06885606

| Study name | The use of transcranial direct current stimulation (tDCS) for vaping reduction |
| Methods | RCT. Double-blind sham-controlled randomized trial |
| Participants | Estimated enrollment: 40  Inclusion criteria: 18 - 65 years; daily regular use of nicotine-containing EC for at least the past 6 months; willing to attend daily appointments for tDCS for two consecutive weeks (Monday through Friday); not interested in or planning to quit vaping in the next 30 days.  Exclusion criteria: Substance use disorder (other than nicotine dependence) (M.I.N.I. SCID) (confirmed with urine drug screen); Current regular use of tobacco cigarettes, nicotine replacement therapy or other medications for smoking cessation; Unstable psychiatric condition; Recent clinically significant head trauma; History of seizures and/or epilepsy\*; Pacemakers or implanted electrical devices such as cochlear implants; Metal embedded in the skull; Skin lesions, open wounds, bruising, or similar injuries on the scalp. |
| Interventions | 1. Active tDCS stimulation group.  Transcranial direct current stimulation (tDCS) is a form of non-invasive brain stimulation that involves brief (e.g., 20-min) application of weak electric current (e.g., 2 mA) to the scalp. Active tDCS intervention increases excitability of neurons at the anode with 20-30% of the current going through the brain from anode to cathode so that both cortical and subcortical structures are stimulated. The procedure is very safe, convenient, and fast-acting with well-established parameters. It has the ability to modulate plasticity in specific brain areas and has established efficacy in human laboratory models of addictive motivation and has been shown to decrease craving for cigarettes when the anode is placed over the left dorsolateral prefrontal cortex (DLPFC).  2. Control. Sham tDCS stimulation group.  Sham tDCS applies a 2mA current for the initial 30 seconds, followed by 0 mA for the remaining 19.5 minutes to simulate active tDCS stimulation. The cathode electrode is placed on the right dorsolateral prefrontal cortex (DLPFC), and the anode is positioned on the left DLPFC, aligned with the electrode placement used in active tDCS. |
| Outcomes | Baseline, 2 weeks. Telephone foloow up at 1 month and 3 months.  Vaping frequency (puffs/day and nicotine pods/week) at end of treatment (2 weeks). The secondary outcome will be e-cigarette craving. Participants will be followed-up via the phone at 1 month and 3 months post randomization respectively. |
| Starting date | Estimated study start date: 2025-04. Estimated study completion date: 2026-05 |
| Contact information | Eunice Chen, M.Sc.yixuan.chen@camh.ca  Kameron Iturralde, B.Sc. Kameron.Iturralde@camh.ca |
| Notes | New ongoing study added to 2025 update. |

NCT06909500

| Study name | A digital intervention (ACT on Vaping App) for vaping cessation in young adult e-cigarette users  Official title: ACT on Vaping: Digital Therapeutic for Young Adult Vaping Cessation |
| Methods | RCT  Country: Fred Hutchinson Cancer Center. USA |
| Participants | Estimated: 1372  Inclusion criteria: Age 18-30; Current weekly user of EC product(s) for the last 30 days; Has a smartphone; either an Android (running version 12 or higher) or iPhone (running iOS version 17 or higher, iPhone 11 or more recent).  Exclusion criteria: using other tobacco cessation treatments; Member of the same household as another research participant; Currently in prison. |
| Interventions | Smartphone App called Acceptance and Commitment Therapy (ACT) on Vaping for helping young adults quit using vapes.  1. Participants use the ACT on Vaping app (Version A) and receive a text messaging program to motivate and support quitting. Participants also receive incentivized text message check-ins at 2 weeks, 2 months, and 4 months to assess their vaping status.  2. Participants use the ACT on Vaping app (Version B) and receive incentivized text message check-ins at 2 weeks, 2 months, and 4 months to assess their vaping status. The control app will also be called ACT on Vaping in participant-facing materials, for blinding purposes; Any outcome evaluator who has direct contact with participants will remain blinded to treatment group assignment. |
| Outcomes | After completion of study intervention, participants are followed up at 3 and 6 months.  Plan to share Individual Participant Data (IPD).  Self-reported 7-day point prevalence abstinence from vaping  Self-reported 30-day point prevalence abstinence from vaping  Biochemically confirmed 7-day abstinence from all nicotine and tobacco (excluding FDA-approved pharmacotherapies).  Self-reported 7-day point prevalence abstinence from all nicotine and tobacco  Self-reported 30-day point prevalence abstinence from all nicotine and tobacco  Prolonged self-reported abstinence from all nicotine and tobacco for 90 days |
| Starting date | Estimated study start date: 2025-09-01. Estimated study completion date: 2027-05-01 |
| Contact information | Jaimee Heffner, PhD, jheffner@fredhutch.org |
| Notes | New ongoing study added to 2025 update. |

NCT06929520

| Study name | The development and evaluation of a culturally grounded ENDS intervention for rural Hawaiian youth |
| Methods | RCT. Randomized Sequential Assignment.  Setting: University of Hawaii, USA |
| Participants | Estimated: 500  Age: 10-14 years  Inclusion Criteria: 6th-8th grade students attending a public or public-charter school on Hawaii Island.  Exclusion Criteria: K-5th grade students and 9th-12th grade students on Hawaii Island. |
| Interventions | 1. Behavioral: ENDS Prevention Intervention (TBD)  The ENDS Prevention Intervention will consist of (1) a modular classroom curriculum with ENDS-focused lessons and lessons covering alcohol, tobacco, and other drug use; (2) social media content that is related to the classroom curriculum and video content, and (3) a print media campaign across school campuses on Hawaii Island.  2. No Intervention: Treatment-as-usual control. Participants will receive treatment-as-usual, which consists of the standard health curriculum delivered in public schools. |
| Outcomes | Baseline and up to 19 months  Past 30-day use of e-cigarettes and/or vaping devices  Past 30-day alcohol, tobacco, and other drug use |
| Starting date | Estimated start date: 2025-10-15. Estimated completion date: 2027-05-15 |
| Contact information | Scott K Okamoto, PhD, University of Hawaii, okamotos@hawaii.edu  Sponsor: University of Hawaii. Collaborators: University of California, Riverside |
| Notes | New ongoing study added at 2025 update. |

Sanchez 2023

| Study name | Supporting youth vaping cessation with the crush the crave smartphone app: protocol for a randomized controlled trial |
| Methods | RCT  Country: Canada  Recruitment: through the Vaping Dependence Cohort—an existing panel of youth enrolled in a prospective cohort study at the University of Toronto who provided consent for recontact in future studies at the Ontario Tobacco Research Unit. |
| Participants | N=600 (estimated)  Age 16-18 and19-29; nicotine vape user (used in the previous 30 days)  Arm 1: Crush the crave = 300 (estimated)  Arm 2: Control = 300 (estimated) |
| Interventions | App based intervention vs control  Intervention arm: Crush the Crave app for vaping cessation. enables users to customize a quit plan. As a tracker app, Crush the Crave monitors the amount of money saved and the number of vape-free days since the user’s quit date. The app tracks cravings and vaping habits. App displays supportive messages and images and links to evidence-based resources, such as quitlines  Control arm: email invitationto complete the baseline assessment, and follow-up assessment questionnaires asking about e-cigarette use and abstinence. After last follow up they will be invited to try the Crush the Crave app, if interested.  Each participant in the intervention and control arms will receive compensation (CAD $10 [US $7.46] electronic gift card) upon completion of the baseline survey questionnaire and each time they complete a follow-up survey throughout the course of the study. |
| Outcomes | Baseline, 3, 6, 9, and 12 months.  self-reported 30-day PPA at 3 months, operationalized as not having vaped, even a puff, in the last 30 days.  Intention to quit smoking in the next 6 months (yes or no), number of puffs per vaping session, number of vape sessions per day, and number of sessions in the past 30 days. |
| Starting date | Recruitment started March 4 2022. |
| Contact information | Michael Chaiton, Michael.Chaiton@camh.ca  Institute for Mental Health Policy Research Centre for Addiction and Mental Health, Toronto, Canada )N, M5s 2S1 |
| Notes | Trial Registration: OSF Registries osf.io/hmd87; https://doi.org/10.17605/OSF.IO/HMD87 |

TCTR20250203006

| Study name | Effectiveness of text messaging as an adjuvant to psychotherapy in school-based smoking cessation programs: a randomized control trial |
| Methods | RCT  Setting: Faculty of Medicine Siriraj Hospital, Mahidol University, Thailand |
| Participants | Age 12-18  Inclusion criteria: 1. Adolescents aged 12-18 years. 2. Have smoked traditional cigarettes and/or e-cigarettes in the 30 days prior to joining the study. 3. Interested in quitting smoking. 4. Able to use electronic devices that can send and receive messages via the Line application.   Exclusion criteria: 1. Adolescents diagnosed with mental health disorders, such as depression, schizophrenia, bipolar disorder, etc. |
| Interventions | Baseline, 3 months  Smoking cessation program, psychotherapy, text messaging,  Screen for tobacco use including other substances. Psycho-education, psycho-support and advise to quit their tobacco use and then 3 months follow-up to assess progress and offer support ex. behavioral treatment if needed. Standard of care as intervention arm 1 plus psychotherapy provided by a psychologist specializing in Cognitive Behavioral Therapy (CBT). Volunteers will receive 3 to 6 counseling sessions, with each session lasting 45 to 60 minutes. Volunteers are required to attend at least 3 sessions, with each session spaced 2 to 4 weeks apart, depending on other co-occurring issues such as family problems, emotional difficulties, or academic challenges. Standard of care and psychotherapy provided by a psychologist as intervention arm 2 plus text messaging. Volunteers will be invited to join the Line official account named Prompt Quit We Can Do It. Each participant will receive one appointment reminder message one week prior to each scheduled therapy session. Additionally, they will receive two messages per week related to smoking cessation, educational content, and motivational support. The messages sent during each period will align with the topics covered in the psychotherapy sessions and will include both text and illustrative content.  No Intervention Behavioral, Active Comparator Behavioral, Experimental Behavioral |
| Outcomes | Baseline, 3 months.  Vaping behavior at start and 3 months after start of the intervention self report questionnaire,  Smoking abstinence at 3 months after start of the intervention self report questionnaire  Risk behaviors and co-occurring problems associated with vaping at start and 3 months after start of the intervention self report questionnaire, factors associated with vaping cessation behavior at start and 3 months after start of the intervention self report questionaire |
| Contact information | Supinya In iw, supinya.ine@mahidol.ac.th  Department of Pediatrics, Faculty of Medicine, Siriraj Hospital, Mahidol University. Princess Maha Chakri Building 2 Wang Lang, Siriraj, Bangkok Noi 10700 Bangkok Thailand  siethics@mahidol.ac.th Human research protection unit. Faculty of Medicine Siriraj Hospital, Mahidol University |
| Notes | New ongoing added to 2025 update. |

## References to studies

### ACTRN12623000022662 {published data only}

- ACTRN12623000022662. The OurFutures Vaping Program: a cluster randomised controlled trial to evaluate the efficacy of a school-based eHealth intervention to prevent e-cigarette use among adolescents. https://trialsearch.who.int/Trial2.aspx?TrialID=ACTRN12623000022662 2023;(accessed 23 July 2024).
- Gardner LA, Rowe AL, Stockings E, Champion KE, Hides L, McBride N et al. Study protocol of the Our Futures Vaping Trial: a cluster randomised controlled trial of a school-based eHealth intervention to prevent e-cigarette use among adolescents. BMC Public Health 2023 Apr 12;23(1):683. [DOI: 10.1186/s12889-023-15609-8]

### ACTRN12625000143426 {published data only}

- ACTRN12625000143426. Replication trial of the OurFutures Vaping Program: evaluation of a school-based eHealth program to prevent e-cigarette use among adolescents in NSW public schools. https://trialsearch.who.int/Trial2.aspx?TrialID=ACTRN12625000143426 accessed 25 June 2025.

### Barnes 2025 {published data only}

- ACTRN12623000079640. Efficacy of a text-message based intervention in preventing adolescent e-cigarette use. Scientific title: A randomised controlled trial to examine the potential effect of a text-message based intervention on preventing adolescent e-cigarette use. https://www.anzctr.org.au/Trial/Registration/TrialReview.aspx?id=385130&showOriginal=true (accessed 25 June 2025).
- Barnes C, Janssen L, Mantach S, McCrabb S, Turon H, Groombridge D et al. Are text-message based programmes targeting adolescents and their parents an acceptable approach to preventing adolescent e-cigarette use? Health Promotion Journal of Australia: official journal of Australian Association of Health Promotion Professionals / 2025;36(2):e70019 2025;36(2):e70019. [DOI: 10.1002/hpja.70019]
- Barnes C, Turon H, McCrabb S, Mantach S, Janssen L, Duffy M, et al. Factorial randomised controlled trial to examine the potential effect of a text message-based intervention on reducing adolescent susceptibility to e-cigarette use: a study protocol. BMJ Open 2024 Aug 17;14(8):e083251. [DOI: 10.1136/bmjopen-2023-083251]

### Borrelli 2025 {published data only}

- Borrelli B, Weinstein D, Endrighi R, Ling N, Koval K, Quintiliani LM, et al. Virtual reality for the prevention and cessation of nicotine vaping in youth: Protocol for a randomized controlled trial. JMIR Research Protocols 2025 May 15;14:e71961. [DOI: 10.2196/71961]
- NCT06003439. Vaping prevention and vaping in youth (Vapechat). https://clinicaltrials.gov/study/NCT06003439 (accessed 25 June 2025).

### Chadi 2023 {published data only}

- Chadi N, Diamant E, Perez T, Al-Saleh A, Sylvestre M-P, O'Loughlin J et al. A brief digital screening and intervention tool for parental and adolescent tobacco and electronic cigarette use in pediatric medical care in Canada: protocol for a pilot randomized controlled trial. JMIR research protocols 2023;12(101599504):e47978-. [DOI: https://dx.doi.org/10.2196/47978]
- NCT05366790. A brief digital screening tool to address tobacco and e-cigarette use in pediatric medical care.. https://clinicaltrials.gov/ct2/show/NCT05366790 2022.

### Champion 2025 {published data only}

- ACTRN12624001492549. Health4Life Parents & Teens: Optimisation of a parent-based intervention to reduce modifiable cancer risk factors among socio-economically disadvantaged adolescents. https://www.anzctr.org.au/Trial/Registration/TrialReview.aspx?ACTRN=12624001492549 (accessed 25 June 2025).
- Champion KE, Davidson L, Hunter E, Thornton L, Spring B, Osman B, et al. Optimising a digital intervention to support parents experiencing socio-economic disadvantage to improve adolescent health behaviours: protocol for the Health4Life Parents & Teens factorial trial. Contemporary clinical trials 2025 2025 Jul;154:107958. [DOI: 10.1016/j.cct.2025.107958]

### CTIS2023-504708-27-00 {published data only}

- CTIS2023-504708-27-00 2023. Cytisine for nicotine-containing electronic cigarette and tobacco cigarette cessation: a randomized placebo-controlled trial. https://trialsearch.who.int/Trial2.aspx?TrialID=CTIS2023-504708-27-00 (accessed 25 June 2025).

### CTIS2023-505036-35-00 {published data only}

- CTIS2023-505036-35-00. A study of nicotine freshmint mouthspray in e-cigarette users willing to quit. https://trialsearch.who.int/Trial2.aspx?TrialID=CTIS2023-505036-35-00.

### Evans 2025 {published data only}

- Evans WD, Ichimiya M, Bingenheimer JB, Cantrell J, D'Esterre AP, Pincus O et al. Design and baseline evaluation of social media vaping prevention trial: Randomized controlled trial study. Journal of Medical Internet Research 2025;27:e. [DOI: 10.2196/72002]
- NCT04867668. Digital media for cancer control (to prevent vaping and smoking behavior) (DMCC). https://clinicaltrials.gov/study/NCT04867668.

### KCT0010346 {published data only}

- KCT0010346 2025. Digital interventions for smoking cessation among young adults in Korea: a randomized controlled trial. https://trialsearch.who.int/Trial2.aspx?TrialID=KCT0010346.

### Krishnan-Sarin 2024 {published data only}

- Krishnan-Sarin S, Kong G, Bold K, Davis D, Cavallo D, Lavallee H, et al. A youth-focussed, virtual, e-cigarette cessation intervention combining in-person CBT with incentives. Drug and Alcohol Dependence 2025;267(Supplement):112259. [DOI: 10.1016/j.drugalcdep.2024.112259]
- Krishnan-Sarin S, Kong G, Bold K, Davis D,  Liss T,  Lavallee H et al. Examining the use of a virtual youth-focussed, e-cigarette cessation intervention combining in-person CBT with abstinence-contingent incentives. In: Society for Research on Nicotine and Tobacco (SRNT) 30th Annual Meeting Edinburgh. Vol. SYM17-2. 20-23 March 2024.

### Lyu 2022 {published data only}

- \*Lyu JC, Olson SS, Ramo DE, Ling PM. Delivering vaping cessation interventions to adolescents and young adults on Instagram: protocol for a randomized controlled trial. BMC public health 2022;22(1):2311-. [DOI: https://dx.doi.org/10.1186/s12889-022-14606-7]
- Ling P, Lyu JC, Lisha N, Rosen S, Belohlavek A, Gribben V et al. E-cigarette cessation support groups on social media: a randomized controlled trial,. Society for Research on Nicotine and Tobacco (SRNT) 31st Annual Meeting 2025, New Orleans, USA 2025 March 12-15submission number 3000722.
- NCT04707911. Social media intervention to stop nicotine and cannabis vaping among adolescents. https://clinicaltrials.gov/show/NCT04707911 (first received 8 July 2024).

### McColgan 2024 {published data only}

- McColgan BJ. Challenges and strategies for recruitment and retention of exclusive e-cigarette users in clinical trials. In: Society for Research on Nicotine and Tobacco (SRNT) 30th Annual Meeting Edinburgh. Vol. COM2-1. 20-23 March 2024.

### NCT04146714 {published data only}

- NCT04146714. Substance use screening to encourage behavior change among young people in primary care. https://clinicaltrials.gov/ct2/show/NCT04146714 2019;(accessed 22 July 2024).

### NCT04898075 {published data only}

- NCT04898075. Quit nicotine: e-cig cessation intervention. https://clinicaltrials.gov/show/NCT04898075 2021;(accessed 23 July 2024).

### NCT05892445 {published data only}

- NCT05892445. Impact of aversive warnings on e-cigarette cessation. https://clinicaltrials.gov/show/NCT05892445 2023;(accessed 23 July 2024).

### NCT05936099 {published data only}

- NCT05936099. Adolescent inpatient tobacco and ENDS intervention. https://clinicaltrials.gov/ct2/show/NCT05936099.
- Wooten S, Catley D, Miller MK, Wilson K, Richter KP, Masonbrink A. Treatment of e-cigarette use among hospitalised adolescents and young adults: a protocol for intervention development and evaluation of preliminary efficacy and implementation outcomes in a randomised controlled trial. BMJ Open 2025;15(1):e094323. [DOI: 10.1136/bmjopen-2024-094323]

### NCT05994209 {published data only}

- NCT05994209. Testing the feasibility and acceptability of social media and digital therapeutics to decrease vaping behaviors. https://clinicaltrials.gov/ct2/show/NCT05994209 2023;(accessed 22 July 2024).

### NCT06027840 {published data only}

- NCT06027840. Concurrent vs. sequential cessation of dual cigarette and e-cigarette use. https://clinicaltrials.gov/ct2/show/NCT06027840 2023;(accessed 22 July 2024).

### NCT06142877 {published data only}

- NCT06142877. Effects of social media use on young adults' e-cigarette use. https://clinicaltrials.gov/ct2/show/NCT06142877 2023;(accessed 22 July 2024).

### NCT06164678 {published data only}

- NCT06164678. Vaping cessation using the Ottawa model for smoking cessation among e-cigarette users. https://clinicaltrials.gov/ct2/show/NCT06164678 2023;(accessed 22 July 2024).

### NCT06196489 {published data only}

- Doran N, Hurst S, Liu J, El-Shahawy O, Myers M, Krebs P. Protocol for the development of a vaping cessation intervention for young adult veterans, Contemporary clinical trials communications 2024;39():101309. Contemporary Clinical Trials Communications 2024;39():101309 2024;39:101309. [DOI: 10.1016/j.conctc.2024.101309]
- NCT06196489. Adapting an intervention for vaping in young veterans. https://clinicaltrials.gov/ct2/show/NCT06196489 2023;(accessed 22 July 2024).

### NCT06395415 {published data only}

- NCT06395415 2024. Development of a mobile health intervention for electronic cigarette use among young adults (EQUIP). https://clinicaltrials.gov/ct2/show/NCT06395415.

### NCT06662305 {published data only}

- NCT06662305. Quit smoking study for people who use e-cigarettes. https://clinicaltrials.gov/study/NCT04946825?tab=results.

### NCT06765291 {published data only}

- NCT06765291. Behavioral intervention for youth to promote vaping cessation. https://clinicaltrials.gov/ct2/show/NCT06765291.

### NCT06832098 {published data only}

- NCT06832098. Nicotine patch plus nicotine mouth spray versus nicotine reduction for vaping cessation. https://clinicaltrials.gov/ct2/show/NCT06832098.

### NCT06862050 {published data only}

- NCT06862050. BREATHE free: a pilot feasibility trial. https://clinicaltrials.gov/ct2/show/NCT06862050.

### NCT06885606 {published data only}

- NCT06885606. The use of TDCS for vaping reduction. https://clinicaltrials.gov/ct2/show/NCT06885606.

### NCT06909500 {published data only}

- NCT06909500. A digital intervention (ACT on Vaping App) for vaping cessation in young adult e-cigarette users. https://clinicaltrials.gov/ct2/show/NCT06909500.

### NCT06929520 {published data only}

- NCT06929520. The development and evaluation of a culturally grounded ENDS intervention for rural Hawaiian youth. https://clinicaltrials.gov/ct2/show/NCT06929520.

### Sanchez 2023 {published data only}

- Sanchez S, Deck Al, Baskerville NB, Chaiton M. Supporting youth vaping cessation with the crush the crave smartphone app: protocol for a randomized controlled trial. JMIR research protocols 2023;12(101599504):e42956. [DOI: https://dx.doi.org/10.2196/42956]
- Sanchez S. Effectiveness of the 'crush the crave' smartphone app on vaping cessation among youth and young adults: a randomized controlled trial. In: Society for Research on Nicotine and Tobacco (SRNT) 30th Annual Meeting Edinburgh. Vol. PPS18-6. 20-23 March 2024.

### TCTR20250203006 {published data only}

- TCTR20250203006. Effectiveness of text messaging as an adjuvant to psychotherapy in school-based smoking cessation programs: a randomized control trial. https://trialsearch.who.int/Trial2.aspx?TrialID=TCTR20250203006.
