# Supplementary material for: Interventions for quitting vaping
Source: Cochrane Database Syst Rev. 2025 Nov 25;2025(11):CD016058. doi: 10.1002/14651858.CD016058.pub3 (PMC12645533; doi:10.1002/14651858.CD016058.pub3)
Supplement: Supplementary file 5 — Supplementary material 5 Analyses [file CD016058-SUP-05-analyses.html]

Analyses


# Supplementary material 5 to: Interventions for quitting vaping

Butler AR, Lindson N, Livingstone-Banks J, Notley C, Turner T, Rigotti NA, Fanshawe TR, Begh R, Wu AD, Brose L, Conde M, Simonavičius E, Hartmann-Boyce J
  
https://doi.org/10.1002/14651858.CD016058.pub3

The material in this section has been supplied by the author(s) for publication under a Licence for Publication and the author(s) are solely responsible for the material. Cochrane has reviewed this material, but Cochrane has not copyedited, formatted or proofread. Cochrane accordingly gives no representations or warranties of any kind in relation to, and accepts no liability for any reliance on or use of, such material.

Back to top

# Analyses

## Analysis group 1: Combination NRT versus no/minimal support

| Analysis or subgroup title | No. of studies | No. of participants | Statistical method | Effect size |
| --- | --- | --- | --- | --- |
| 1.1 Vaping cessation at 6 months or longer | 2 | 214 | Risk Ratio (M-H, Random, 95% CI) | 0.96 [0.73, 1.25] |
| 1.2 Combustible tobacco abstinence at 6 months or longer | 1 |  | Risk Ratio (M-H, Random, 95% CI) | Totals not selected |
| 1.3 Number of participants reporting SAEs | 2 |  | Risk Ratio (M-H, Random, 95% CI) | Totals not selected |
| 1.4 Vaping cessation at between 3 & 6 months | 3 | 722 | Risk Ratio (M-H, Random, 95% CI) | 1.01 [0.75, 1.35] |
| 1.5 Combustible tobacco abstinence at between 3 & 6 months | 1 |  | Risk Ratio (M-H, Random, 95% CI) | Totals not selected |
| 1.6 Number of participants reporting AEs | 2 |  | Risk Ratio (M-H, Random, 95% CI) | Totals not selected |
| 1.7 Weight (lbs) at longest follow-up | 1 |  | Mean Difference (IV, Random, 95% CI) | Totals not selected |
| 1.8 Systolic blood pressure (mmHg) at longest follow-up | 1 |  | Mean Difference (IV, Random, 95% CI) | Totals not selected |
| 1.9 Heart rate (bpm) at longest follow-up | 1 |  | Mean Difference (IV, Random, 95% CI) | Totals not selected |

## Analysis group 2: Cytisine versus placebo

| Analysis or subgroup title | No. of studies | No. of participants | Statistical method | Effect size |
| --- | --- | --- | --- | --- |
| 2.1 Number of participants reporting SAEs | 1 |  | Risk Ratio (M-H, Random, 95% CI) | Totals not selected |
| 2.2 Vaping cessation at between 3 & 6 months | 1 |  | Risk Ratio (M-H, Random, 95% CI) | Totals not selected |
| 2.3 Change in combustible tobacco product use (tobacco cigarette use) at between 3 & 6 months | 1 |  | Risk Ratio (M-H, Random, 95% CI) | Totals not selected |
| 2.4 Number of participants reporting AEs | 1 |  | Risk Ratio (M-H, Random, 95% CI) | Totals not selected |
| 2.5 Mean change in systolic blood pressure (mmHg) | 1 |  | Mean Difference (IV, Random, 95% CI) | Totals not selected |
| 2.6 Mean change in diastolic blood pressure (mmHg) | 1 |  | Mean Difference (IV, Random, 95% CI) | Totals not selected |
| 2.7 Mean change in heart rate (bpm) | 1 |  | Mean Difference (IV, Random, 95% CI) | Totals not selected |
| 2.8 Cotinine (ng/ml) at longest follow-up | 1 |  | Mean Difference (IV, Random, 95% CI) | Totals not selected |

## Analysis group 3: Varenicline versus placebo

| Analysis or subgroup title | No. of studies | No. of participants | Statistical method | Effect size |
| --- | --- | --- | --- | --- |
| 3.1 Vaping cessation at 6 months or longer | 2 | 315 | Risk Ratio (M-H, Random, 95% CI) | 2.71 [1.33, 5.49] |
| 3.1.1 18 years and over | 1 | 140 | Risk Ratio (M-H, Random, 95% CI) | 2.00 [1.09, 3.68] |
| 3.1.2 Both under and over 18 years | 1 | 175 | Risk Ratio (M-H, Random, 95% CI) | 4.12 [1.78, 9.54] |
| 3.1.3 Under 18 years | 0 | 0 | Risk Ratio (M-H, Random, 95% CI) | Not estimable |
| 3.2 Number of participants reporting SAEs | 4 | 304 | Risk Ratio (M-H, Random, 95% CI) | 2.82 [0.45, 17.59] |
| 3.2.1 18 years and over | 3 | 130 | Risk Ratio (M-H, Random, 95% CI) | 2.60 [0.11, 62.16] |
| 3.2.2 Both under and over 18 years | 1 | 174 | Risk Ratio (M-H, Random, 95% CI) | 2.93 [0.31, 27.64] |
| 3.2.3 Under 18 years | 0 | 0 | Risk Ratio (M-H, Random, 95% CI) | Not estimable |
| 3.3 Vaping cessation at between 3 & 6 months | 4 | 357 | Risk Ratio (M-H, Random, 95% CI) | 2.04 [1.11, 3.75] |
| 3.3.1 18 years and over | 3 | 182 | Risk Ratio (M-H, Random, 95% CI) | 1.64 [0.98, 2.74] |
| 3.3.2 Both under and over 18 years | 1 | 175 | Risk Ratio (M-H, Random, 95% CI) | 3.71 [2.11, 6.51] |
| 3.3.3 Under 18 years | 0 | 0 | Risk Ratio (M-H, Random, 95% CI) | Not estimable |
| 3.4 Number of participants reporting AEs | 4 | 304 | Risk Ratio (M-H, Random, 95% CI) | 1.10 [0.98, 1.24] |
| 3.4.1 18 years and over | 3 | 130 | Risk Ratio (M-H, Random, 95% CI) | 1.19 [0.84, 1.68] |
| 3.4.2 Both under and over 18 years | 1 | 174 | Risk Ratio (M-H, Random, 95% CI) | 1.09 [0.95, 1.25] |
| 3.4.3 Under 18 years | 0 | 0 | Risk Ratio (M-H, Random, 95% CI) | Not estimable |
| 3.5 Weight (lbs) at longest follow-up | 1 |  | Mean Difference (IV, Random, 95% CI) | Totals not selected |
| 3.6 Systolic blood pressure (mmHg) at longest follow-up | 1 |  | Mean Difference (IV, Random, 95% CI) | Totals not selected |
| 3.7 Diastolic blood pressure (mmHg) at longest follow-up | 1 |  | Mean Difference (IV, Random, 95% CI) | Totals not selected |
| 3.8 Heart rate (bpm) at longest follow-up | 1 |  | Mean Difference (IV, Random, 95% CI) | Totals not selected |

## Analysis group 4: Nicotine/vaping reduction versus minimal support

| Analysis or subgroup title | No. of studies | No. of participants | Statistical method | Effect size |
| --- | --- | --- | --- | --- |
| 4.1 Vaping cessation at 6 months or longer | 1 |  | Risk Ratio (M-H, Random, 95% CI) | Totals not selected |
| 4.2 Vaping cessation at between 3 & 6 months | 1 |  | Risk Ratio (M-H, Random, 95% CI) | Totals not selected |
| 4.3 Weight (lbs) at longest follow-up | 1 |  | Mean Difference (IV, Random, 95% CI) | Totals not selected |
| 4.4 Systolic blood pressure (mmHg) at longest follow-up | 1 |  | Mean Difference (IV, Random, 95% CI) | Totals not selected |
| 4.5 Heart rate (bpm) at longest follow-up | 1 |  | Mean Difference (IV, Random, 95% CI) | Totals not selected |

## Analysis group 5: Text message versus no/minimal support

| Analysis or subgroup title | No. of studies | No. of participants | Statistical method | Effect size |
| --- | --- | --- | --- | --- |
| 5.1 Vaping cessation at 6 months or longer | 2 | 4091 | Risk Ratio (M-H, Random, 95% CI) | 1.32 [1.19, 1.47] |
| 5.1.1 18 years and over | 0 | 0 | Risk Ratio (M-H, Random, 95% CI) | Not estimable |
| 5.1.2 Both under and over 18 years | 1 | 2588 | Risk Ratio (M-H, Random, 95% CI) | 1.29 [1.11, 1.50] |
| 5.1.3 Under 18 years | 1 | 1503 | Risk Ratio (M-H, Random, 95% CI) | 1.35 [1.17, 1.57] |
| 5.2 Combustible tobacco abstinence at 6 months or longer among dual users at baseline | 1 |  | Risk Ratio (M-H, Random, 95% CI) | Totals not selected |
| 5.3 Combustible tobacco use uptake at 6 months or longer among exclusive vape users at baseline | 1 |  | Risk Ratio (M-H, Random, 95% CI) | Totals not selected |
| 5.4 Number of participants reporting SAEs | 3 |  | Risk Ratio (M-H, Random, 95% CI) | Totals not selected |
| 5.4.1 18 years and over | 1 |  | Risk Ratio (M-H, Random, 95% CI) | Totals not selected |
| 5.4.2 Both under and over 18 years | 1 |  | Risk Ratio (M-H, Random, 95% CI) | Totals not selected |
| 5.4.3 Under 18 years | 1 |  | Risk Ratio (M-H, Random, 95% CI) | Totals not selected |
| 5.5 Vaping cessation at between 3 & 6 months | 2 | 576 | Risk Ratio (M-H, Random, 95% CI) | 1.04 [0.84, 1.29] |
| 5.5.1 18 years and over | 1 | 508 | Risk Ratio (M-H, Random, 95% CI) | 1.05 [0.84, 1.31] |
| 5.5.2 Both under and over 18 years | 1 | 68 | Risk Ratio (M-H, Random, 95% CI) | 0.89 [0.28, 2.79] |
| 5.5.3 Under 18 years | 0 | 0 | Risk Ratio (M-H, Random, 95% CI) | Not estimable |
| 5.6 Number of participants reporting AEs | 3 |  | Risk Ratio (M-H, Random, 95% CI) | Totals not selected |
| 5.6.1 18 years and over | 1 |  | Risk Ratio (M-H, Random, 95% CI) | Totals not selected |
| 5.6.2 Both under and over 18 years | 1 |  | Risk Ratio (M-H, Random, 95% CI) | Totals not selected |
| 5.6.3 Under 18 years | 1 |  | Risk Ratio (M-H, Random, 95% CI) | Totals not selected |

## Analysis group 6: Financial incentives versus no/minimal support

| Analysis or subgroup title | No. of studies | No. of participants | Statistical method | Effect size |
| --- | --- | --- | --- | --- |
| 6.1 Vaping cessation at between 3 & 6 months | 1 |  | Risk Ratio (M-H, Random, 95% CI) | Totals not selected |

## Analysis group 7: Media literacy e-learning versus no/minimal support

| Analysis or subgroup title | No. of studies | No. of participants | Statistical method | Effect size |
| --- | --- | --- | --- | --- |
| 7.1 Vaping cessation at between 3 & 6 months | 1 |  | Risk Ratio (M-H, Random, 95% CI) | Totals not selected |

## Analysis group 8: App-based intervention + text messaging versus no/minimal support

| Analysis or subgroup title | No. of studies | No. of participants | Statistical method | Effect size |
| --- | --- | --- | --- | --- |
| 8.1 Participants reporting SAEs | 1 |  | Risk Ratio (M-H, Random, 95% CI) | Totals not selected |
| 8.2 Vaping cessation at between 3 & 6 months | 1 |  | Risk Ratio (M-H, Random, 95% CI) | Totals not selected |
| 8.3 Participants reporting AEs | 1 |  | Risk Ratio (M-H, Random, 95% CI) | Totals not selected |

## Analysis group 9: Media literacy e-learning + financial incentives versus no/minimal support

| Analysis or subgroup title | No. of studies | No. of participants | Statistical method | Effect size |
| --- | --- | --- | --- | --- |
| 9.1 Vaping cessation at between 3 & 6 months | 1 |  | Risk Ratio (M-H, Random, 95% CI) | Totals not selected |

## Analysis group 10: Combination NRT + print-based self-help versus no/minimal support

| Analysis or subgroup title | No. of studies | No. of participants | Statistical method | Effect size |
| --- | --- | --- | --- | --- |
| 10.1 Number of participants reporting SAEs | 1 |  | Risk Ratio (M-H, Random, 95% CI) | Totals not selected |

## Analysis group 11: Combination NRT + text message versus no/minimal intervention

| Analysis or subgroup title | No. of studies | No. of participants | Statistical method | Effect size |
| --- | --- | --- | --- | --- |
| 11.1 Numbers of participants reporting SAEs | 1 |  | Risk Ratio (M-H, Random, 95% CI) | Totals not selected |
| 11.2 Vaping cessation at between 3 & 6 months | 1 |  | Risk Ratio (M-H, Random, 95% CI) | Totals not selected |
| 11.3 Number of participants reporting AEs | 1 |  | Risk Ratio (M-H, Random, 95% CI) | Totals not selected |

## Analysis group 12: High dose (42 mg patch + 4 mg lozenge) versus standard dose (21 mg patch + 4 mg lozenge) NRT

| Analysis or subgroup title | No. of studies | No. of participants | Statistical method | Effect size |
| --- | --- | --- | --- | --- |
| 12.1 Participants reporting AEs | 1 |  | Risk Ratio (M-H, Random, 95% CI) | Totals not selected |

## Analysis group 13: Mid-dose (35 mg patch + 4 mg lozenge) versus standard dose (21 mg patch + 4 mg lozenge) NRT

| Analysis or subgroup title | No. of studies | No. of participants | Statistical method | Effect size |
| --- | --- | --- | --- | --- |
| 13.1 Participants reporting AEs | 1 |  | Risk Ratio (M-H, Random, 95% CI) | Totals not selected |

## Analysis group 14: High dose (42 mg patch + 4 mg lozenge) versus mid-dose (35 mg + 4 mg lozenge) NRT

| Analysis or subgroup title | No. of studies | No. of participants | Statistical method | Effect size |
| --- | --- | --- | --- | --- |
| 14.1 Participants reporting AEs | 1 |  | Risk Ratio (M-H, Random, 95% CI) | Totals not selected |

## Analysis group 15: Combination NRT versus nicotine/vaping reduction

| Analysis or subgroup title | No. of studies | No. of participants | Statistical method | Effect size |
| --- | --- | --- | --- | --- |
| 15.1 Vaping cessation at 6 months or longer | 1 |  | Risk Ratio (M-H, Random, 95% CI) | Totals not selected |
| 15.2 Vaping cessation at between 3 & 6 months | 1 |  | Risk Ratio (M-H, Random, 95% CI) | Totals not selected |
| 15.3 Weight (lbs) at longest follow-up | 1 |  | Mean Difference (IV, Random, 95% CI) | Totals not selected |
| 15.4 Systolic blood pressure (mmHg) at longest follow-up | 1 |  | Mean Difference (IV, Random, 95% CI) | Totals not selected |
| 15.5 Heart rate (bpm) at longest follow-up | 1 |  | Mean Difference (IV, Random, 95% CI) | Totals not selected |

## Analysis group 16: Combination NRT versus text message

| Analysis or subgroup title | No. of studies | No. of participants | Statistical method | Effect size |
| --- | --- | --- | --- | --- |
| 16.1 Numbers of participants reporting SAEs | 1 |  | Risk Ratio (M-H, Random, 95% CI) | Totals not selected |
| 16.2 Vaping cessation at between 3 & 6 months | 1 |  | Risk Ratio (M-H, Random, 95% CI) | Totals not selected |
| 16.3 Number of participants reporting AEs | 1 |  | Risk Ratio (M-H, Random, 95% CI) | Totals not selected |

## Analysis group 17: Media literacy e-learning versus financial incentives

| Analysis or subgroup title | No. of studies | No. of participants | Statistical method | Effect size |
| --- | --- | --- | --- | --- |
| 17.1 Vaping cessation at between 3 & 6 months | 1 |  | Risk Ratio (M-H, Random, 95% CI) | Totals not selected |

# Figures and tables

Analysis 1.1: Vaping cessation at 6 months or longer


Study or Subgroup
Klemperer 2025
Sahr 2021

Total (Wald
a
)
Total events:
Test for overall effect: Z = 0.33 (P = 0.74)

Heterogeneity: Tau² (DL
b
) = 0.00; Chi² = 0.81, df = 1 (P = 0.37); I² = 0%

Combination NRT
Events
51
2
53
Total
102
7

109

No/minimal support
Events
51
1
52
Total
96
9

105
Weight
98.5%
1.5%

100.0%

Risk Ratio
M-H, Random, 95% CI
0.94 [0.72 , 1.23]
2.57 [0.29 , 22.93]

0.96 [0.73 , 1.25]

Risk Ratio
M-H, Random, 95% CI


0.01

0.1

1

10

100


Favours no/minimal support

Favours combination NRT


Risk of Bias
A

?

+
B

?

+
C

−

+
D

+

−
E

+

+
F

+

+
G

+

+

Footnotes

a
CI calculated by Wald-type method.

b
Tau² calculated by DerSimonian and Laird method.
Risk of bias legend

(A) Random sequence generation (selection bias)

(B) Allocation concealment (selection bias)

(C) Blinding of participants and personnel (performance bias)

(D) Blinding of outcome assessment (detection bias)

(E) Incomplete outcome data (attrition bias)

(F) Selective reporting (reporting bias)

(G) Other bias


Analysis 1.2: Combustible tobacco abstinence at 6 months or longer


Study or Subgroup
Klemperer 2025

Combination NRT
Events
43
Total
102

No/minimal support
Events
41
Total
96

Risk Ratio
M-H, Random, 95% CI
0.99 [0.71 , 1.37]

Risk Ratio
M-H, Random, 95% CI


0.1

0.2

0.5

1

2

5

10


Favours no/minimal support

Favours combination NRT

Risk of Bias
A

?
B

?
C

−
D

+
E

+
F

+
G

+

Risk of bias legend

(A) Random sequence generation (selection bias)

(B) Allocation concealment (selection bias)

(C) Blinding of participants and personnel (performance bias)

(D) Blinding of outcome assessment (detection bias)

(E) Incomplete outcome data (attrition bias)

(F) Selective reporting (reporting bias)

(G) Other bias


Analysis 1.3: Number of participants reporting SAEs


Study or Subgroup
Klein 2024
Klemperer 2025

Combination NRT
Events
0
0
Total
248
102

No/minimal support
Events
0
0
Total
260
96

Risk Ratio
M-H, Random, 95% CI
Not estimable
Not estimable

Risk Ratio
M-H, Random, 95% CI


0.1

0.2

0.5

1

2

5

10


Favours combination NRT

Favours no/minimal support

Risk of Bias
A

?

?
B

?

?
C

−

−
D

+

+
E

+

+
F

+

+
G

+

+

Risk of bias legend

(A) Random sequence generation (selection bias)

(B) Allocation concealment (selection bias)

(C) Blinding of participants and personnel (performance bias)

(D) Blinding of outcome assessment (detection bias)

(E) Incomplete outcome data (attrition bias)

(F) Selective reporting (reporting bias)

(G) Other bias


Analysis 1.4: Vaping cessation at between 3 & 6 months


Study or Subgroup
Klein 2024
Klemperer 2025
Sahr 2021

Total (Wald
a
)
Total events:
Test for overall effect: Z = 0.04 (P = 0.97)

Heterogeneity: Tau² (DL
b
) = 0.03; Chi² = 4.33, df = 2 (P = 0.11); I² = 54%

Combination NRT
Events
105
50
3
158
Total
248
102
7

357

No/minimal support
Events
91
51
7
149
Total
260
96
9

365
Weight
48.7%
42.5%
8.8%

100.0%

Risk Ratio
M-H, Random, 95% CI
1.21 [0.97 , 1.51]
0.92 [0.70 , 1.21]
0.55 [0.22 , 1.39]

1.01 [0.75 , 1.35]

Risk Ratio
M-H, Random, 95% CI


0.1

0.2

0.5

1

2

5

10


Favours no/minimal support

Favours combination NRT


Risk of Bias
A

?

?

+
B

?

?

+
C

−

−

+
D

+

+

−
E

+

+

+
F

+

+

+
G

+

+

+

Footnotes

a
CI calculated by Wald-type method.

b
Tau² calculated by DerSimonian and Laird method.
Risk of bias legend

(A) Random sequence generation (selection bias)

(B) Allocation concealment (selection bias)

(C) Blinding of participants and personnel (performance bias)

(D) Blinding of outcome assessment (detection bias)

(E) Incomplete outcome data (attrition bias)

(F) Selective reporting (reporting bias)

(G) Other bias


Analysis 1.5: Combustible tobacco abstinence at between 3 & 6 months


Study or Subgroup
Klemperer 2025

Combination NRT
Events
21
Total
102

No/minimal support
Events
12
Total
96

Risk Ratio
M-H, Random, 95% CI
1.65 [0.86 , 3.16]

Risk Ratio
M-H, Random, 95% CI


0.02

0.1

1

10

50


Favours no/minimal support

Favours combination NRT

Risk of Bias
A

?
B

?
C

−
D

+
E

+
F

+
G

+

Risk of bias legend

(A) Random sequence generation (selection bias)

(B) Allocation concealment (selection bias)

(C) Blinding of participants and personnel (performance bias)

(D) Blinding of outcome assessment (detection bias)

(E) Incomplete outcome data (attrition bias)

(F) Selective reporting (reporting bias)

(G) Other bias


Analysis 1.6: Number of participants reporting AEs


Study or Subgroup
Klein 2024
Klemperer 2025

Combination NRT
Events
66
11
Total
190
102

No/minimal support
Events
24
12
Total
189
96

Risk Ratio
M-H, Random, 95% CI
2.74 [1.79 , 4.17]
0.86 [0.40 , 1.86]

Risk Ratio
M-H, Random, 95% CI


0.1

0.2

0.5

1

2

5

10


Favours combination NRT

Favours no/minimal support

Risk of Bias
A

?

?
B

?

?
C

−

−
D

+

+
E

+

+
F

+

+
G

+

+

Risk of bias legend

(A) Random sequence generation (selection bias)

(B) Allocation concealment (selection bias)

(C) Blinding of participants and personnel (performance bias)

(D) Blinding of outcome assessment (detection bias)

(E) Incomplete outcome data (attrition bias)

(F) Selective reporting (reporting bias)

(G) Other bias


Analysis 1.7: Weight (lbs) at longest follow-up


Study or Subgroup
Sahr 2021

Combination NRT
Mean [lb]
205.8
SD [lb]
46.86
Total
5

No/minimal support
Mean [lb]
171.88
SD [lb]
38.81
Total
6

Mean Difference
IV, Random, 95% CI [lb]
33.92 [-17.57 , 85.41]

Mean Difference
IV, Random, 95% CI [lb]


-100

-50

0

50

100


Risk of Bias
A

+
B

+
C

+
D

−
E

+
F

+
G

+

Risk of bias legend

(A) Random sequence generation (selection bias)

(B) Allocation concealment (selection bias)

(C) Blinding of participants and personnel (performance bias)

(D) Blinding of outcome assessment (detection bias)

(E) Incomplete outcome data (attrition bias)

(F) Selective reporting (reporting bias)

(G) Other bias


Analysis 1.8: Systolic blood pressure (mmHg) at longest follow-up


Study or Subgroup
Sahr 2021

Combination NRT
Mean [mmHg]
136.2
SD [mmHg]
13.04
Total
5

No/minimal support
Mean [mmHg]
127.88
SD [mmHg]
6.94
Total
9

Mean Difference
IV, Random, 95% CI [mmHg]
8.32 [-3.98 , 20.62]

Mean Difference
IV, Random, 95% CI [mmHg]


-50

-25

0

25

50


Risk of Bias
A

+
B

+
C

+
D

−
E

+
F

+
G

+

Risk of bias legend

(A) Random sequence generation (selection bias)

(B) Allocation concealment (selection bias)

(C) Blinding of participants and personnel (performance bias)

(D) Blinding of outcome assessment (detection bias)

(E) Incomplete outcome data (attrition bias)

(F) Selective reporting (reporting bias)

(G) Other bias


Analysis 1.9: Heart rate (bpm) at longest follow-up


Study or Subgroup
Sahr 2021

Combination NRT
Mean [bpm]
71.4
SD [bpm]
17.6
Total
5

No/minimal support
Mean [bpm]
75.63
SD [bpm]
19.62
Total
9

Mean Difference
IV, Random, 95% CI [bpm]
-4.23 [-24.29 , 15.83]

Mean Difference
IV, Random, 95% CI [bpm]


-50

-25

0

25

50


Favours combination NRT

Favours no/minimal support

Risk of Bias
A

+
B

+
C

+
D

−
E

+
F

+
G

+

Risk of bias legend

(A) Random sequence generation (selection bias)

(B) Allocation concealment (selection bias)

(C) Blinding of participants and personnel (performance bias)

(D) Blinding of outcome assessment (detection bias)

(E) Incomplete outcome data (attrition bias)

(F) Selective reporting (reporting bias)

(G) Other bias


Analysis 2.1: Number of participants reporting SAEs


Study or Subgroup
Rigotti 2024

Cytisine
Events
0
Total
106

Placebo
Events
0
Total
53

Risk Ratio
M-H, Random, 95% CI
Not estimable

Risk Ratio
M-H, Random, 95% CI


0.5

0.7

1

1.5

2


Favours cytisine

Favours placebo

Risk of Bias
A

+
B

+
C

+
D

+
E

+
F

+
G

+

Risk of bias legend

(A) Random sequence generation (selection bias)

(B) Allocation concealment (selection bias)

(C) Blinding of participants and personnel (performance bias)

(D) Blinding of outcome assessment (detection bias)

(E) Incomplete outcome data (attrition bias)

(F) Selective reporting (reporting bias)

(G) Other bias


Analysis 2.2: Vaping cessation at between 3 & 6 months


Study or Subgroup
Rigotti 2024

Cytisine
Events
25
Total
107

Placebo
Events
7
Total
53

Risk Ratio
M-H, Random, 95% CI
1.77 [0.82 , 3.82]

Risk Ratio
M-H, Random, 95% CI


0.1

0.2

0.5

1

2

5

10


Favours placebo

Favours cytisine

Risk of Bias
A

+
B

+
C

+
D

+
E

+
F

+
G

+

Risk of bias legend

(A) Random sequence generation (selection bias)

(B) Allocation concealment (selection bias)

(C) Blinding of participants and personnel (performance bias)

(D) Blinding of outcome assessment (detection bias)

(E) Incomplete outcome data (attrition bias)

(F) Selective reporting (reporting bias)

(G) Other bias


Analysis 2.3: Change in combustible tobacco product use (tobacco cigarette use) at between 3 & 6 months


Study or Subgroup
Rigotti 2024

Cytisine
Events
8
Total
107

Placebo
Events
6
Total
53

Risk Ratio
M-H, Random, 95% CI
0.66 [0.24 , 1.81]

Risk Ratio
M-H, Random, 95% CI


0.1

0.2

0.5

1

2

5

10


Favours cytisine

Favours placebo

Risk of Bias
A

+
B

+
C

+
D

+
E

+
F

+
G

+

Risk of bias legend

(A) Random sequence generation (selection bias)

(B) Allocation concealment (selection bias)

(C) Blinding of participants and personnel (performance bias)

(D) Blinding of outcome assessment (detection bias)

(E) Incomplete outcome data (attrition bias)

(F) Selective reporting (reporting bias)

(G) Other bias


Analysis 2.4: Number of participants reporting AEs


Study or Subgroup
Rigotti 2024

Cytisine
Events
54
Total
106

Placebo
Events
29
Total
53

Risk Ratio
M-H, Random, 95% CI
0.93 [0.68 , 1.27]

Risk Ratio
M-H, Random, 95% CI


0.5

0.7

1

1.5

2


Favours cytisine

Favours placebo

Risk of Bias
A

+
B

+
C

+
D

+
E

+
F

+
G

+

Risk of bias legend

(A) Random sequence generation (selection bias)

(B) Allocation concealment (selection bias)

(C) Blinding of participants and personnel (performance bias)

(D) Blinding of outcome assessment (detection bias)

(E) Incomplete outcome data (attrition bias)

(F) Selective reporting (reporting bias)

(G) Other bias


Analysis 2.5: Mean change in systolic blood pressure (mmHg)


Study or Subgroup
Rigotti 2024

Cytisine
Mean [mmHg]
0.3
SD [mmHg]
12.96
Total
89

Placebo
Mean [mmHg]
-0.6
SD [mmHg]
10.74
Total
41

Mean Difference
IV, Random, 95% CI [mmHg]
0.90 [-3.35 , 5.15]

Mean Difference
IV, Random, 95% CI [mmHg]


-10

-5

0

5

10


Risk of Bias
A

+
B

+
C

+
D

+
E

+
F

+
G

+

Risk of bias legend

(A) Random sequence generation (selection bias)

(B) Allocation concealment (selection bias)

(C) Blinding of participants and personnel (performance bias)

(D) Blinding of outcome assessment (detection bias)

(E) Incomplete outcome data (attrition bias)

(F) Selective reporting (reporting bias)

(G) Other bias


Analysis 2.6: Mean change in diastolic blood pressure (mmHg)


Study or Subgroup
Rigotti 2024

Cytisine
Mean [mmHg]
-1.2
SD [mmHg]
9.32
Total
89

Placebo
Mean [mmHg]
1.3
SD [mmHg]
8.41
Total
41

Mean Difference
IV, Random, 95% CI [mmHg]
-2.50 [-5.72 , 0.72]

Mean Difference
IV, Random, 95% CI [mmHg]


-10

-5

0

5

10


Risk of Bias
A

+
B

+
C

+
D

+
E

+
F

+
G

+

Risk of bias legend

(A) Random sequence generation (selection bias)

(B) Allocation concealment (selection bias)

(C) Blinding of participants and personnel (performance bias)

(D) Blinding of outcome assessment (detection bias)

(E) Incomplete outcome data (attrition bias)

(F) Selective reporting (reporting bias)

(G) Other bias


Analysis 2.7: Mean change in heart rate (bpm)


Study or Subgroup
Rigotti 2024

Cytisine
Mean [bpm]
3
SD [bpm]
11.56
Total
89

Placebo
Mean [bpm]
2.4
SD [bpm]
12.2
Total
41

Mean Difference
IV, Random, 95% CI [bpm]
0.60 [-3.84 , 5.04]

Mean Difference
IV, Random, 95% CI [bpm]


-10

-5

0

5

10


Favours cytisine

Favours placebo

Risk of Bias
A

+
B

+
C

+
D

+
E

+
F

+
G

+

Risk of bias legend

(A) Random sequence generation (selection bias)

(B) Allocation concealment (selection bias)

(C) Blinding of participants and personnel (performance bias)

(D) Blinding of outcome assessment (detection bias)

(E) Incomplete outcome data (attrition bias)

(F) Selective reporting (reporting bias)

(G) Other bias


Analysis 2.8: Cotinine (ng/ml) at longest follow-up


Study or Subgroup
Rigotti 2024

Cytisine
Mean [ng/mL]
181.74
SD [ng/mL]
188.91
Total
87

Placebo
Mean [ng/mL]
211.69
SD [ng/mL]
199.37
Total
39

Mean Difference
IV, Random, 95% CI [ng/mL]
-29.95 [-104.05 , 44.15]

Mean Difference
IV, Random, 95% CI [ng/mL]


-100

-50

0

50

100


Favours cytisine

Favours placebo

Risk of Bias
A

+
B

+
C

+
D

+
E

+
F

+
G

+

Risk of bias legend

(A) Random sequence generation (selection bias)

(B) Allocation concealment (selection bias)

(C) Blinding of participants and personnel (performance bias)

(D) Blinding of outcome assessment (detection bias)

(E) Incomplete outcome data (attrition bias)

(F) Selective reporting (reporting bias)

(G) Other bias


Analysis 3.1: Vaping cessation at 6 months or longer


Study or Subgroup

3.1.1 18 years and over
Caponnetto 2023

Subtotal
Total events:
Test for overall effect: Z = 2.23 (P = 0.03)

Heterogeneity: Not applicable

3.1.2 Both under and over 18 years
Evins 2025

Subtotal
Total events:
Test for overall effect: Z = 3.30 (P = 0.0010)

Heterogeneity: Not applicable

3.1.3 Under 18 years

Subtotal
Total events:
Test for overall effect: Not applicable

Heterogeneity: Not applicable

Total (Wald
a
)
Total events:
Test for overall effect: Z = 2.76 (P = 0.006)
Test for subgroup differences: Chi² = 1.86, df = 1 (P = 0.17), I² = 46.3%

Heterogeneity: Tau² (DL
b
) = 0.13; Chi² = 1.91, df = 1 (P = 0.17); I² = 48%

Varenicline
Events
24
24
25
25
0
49
Total
70

70
88

88

0

158

Placebo
Events
12
12
6
6
0
18
Total
70

70
87

87

0

157
Weight
58.2%

58.2%
41.8%

41.8%

100.0%

Risk Ratio
M-H, Random, 95% CI
2.00 [1.09 , 3.68]

2.00 [1.09 , 3.68]
4.12 [1.78 , 9.54]

4.12 [1.78 , 9.54]

Not estimable

2.71 [1.33 , 5.49]

Risk Ratio
M-H, Random, 95% CI


0.1

0.2

0.5

1

2

5

10


Favours placebo

Favours varenicline


Risk of Bias
A

+

+
B

+

+
C

+

+
D

+

+
E

+

+
F

+

+
G

+

+

Footnotes

a
CI calculated by Wald-type method.

b
Tau² calculated by DerSimonian and Laird method.
Risk of bias legend

(A) Random sequence generation (selection bias)

(B) Allocation concealment (selection bias)

(C) Blinding of participants and personnel (performance bias)

(D) Blinding of outcome assessment (detection bias)

(E) Incomplete outcome data (attrition bias)

(F) Selective reporting (reporting bias)

(G) Other bias


Analysis 3.2: Number of participants reporting SAEs


Study or Subgroup

3.2.1 18 years and over
Caponnetto 2023
Fucito 2024
NCT04602494

Subtotal
Total events:
Test for overall effect: Z = 0.59 (P = 0.56)

Heterogeneity: Not applicable

3.2.2 Both under and over 18 years
Evins 2025

Subtotal
Total events:
Test for overall effect: Z = 0.94 (P = 0.35)

Heterogeneity: Not applicable

3.2.3 Under 18 years

Subtotal
Total events:
Test for overall effect: Not applicable

Heterogeneity: Not applicable

Total (Wald
a
)
Total events:
Test for overall effect: Z = 1.11 (P = 0.27)
Test for subgroup differences: Chi² = 0.00, df = 1 (P = 0.95), I² = 0%

Heterogeneity: Tau² (DL
b
) = 0.00; Chi² = 0.00, df = 1 (P = 0.95); I² = 0%

Varenicline
Events
1
0
0
1
3
3
0
4
Total
51
18
1

70
88

88

0

158

Placebo
Events
0
0
0
0
1
1
0
1
Total
44
15
1

60
86

86

0

146
Weight
33.3%

33.3%
66.7%

66.7%

100.0%

Risk Ratio
M-H, Random, 95% CI
2.60 [0.11 , 62.16]
Not estimable
Not estimable

2.60 [0.11 , 62.16]
2.93 [0.31 , 27.64]

2.93 [0.31 , 27.64]

Not estimable

2.82 [0.45 , 17.59]

Risk Ratio
M-H, Random, 95% CI


0.01

0.1

1

10

100


Favours varenicline

Favours placebo


Risk of Bias
A

+

?

+

+
B

+

?

+

+
C

+

+

+

+
D

+

+

+

+
E

+

+

−

+
F

+

+

+

+
G

+

+

+

+

Footnotes

a
CI calculated by Wald-type method.

b
Tau² calculated by DerSimonian and Laird method.
Risk of bias legend

(A) Random sequence generation (selection bias)

(B) Allocation concealment (selection bias)

(C) Blinding of participants and personnel (performance bias)

(D) Blinding of outcome assessment (detection bias)

(E) Incomplete outcome data (attrition bias)

(F) Selective reporting (reporting bias)

(G) Other bias


Analysis 3.3: Vaping cessation at between 3 & 6 months


Study or Subgroup

3.3.1 18 years and over
Caponnetto 2023
Fucito 2024
NCT04602494

Subtotal (Wald
a
)
Total events:
Test for overall effect: Z = 1.88 (P = 0.06)

Heterogeneity: Tau² (DL
b
) = 0.03; Chi² = 2.24, df = 2 (P = 0.33); I² = 11%

3.3.2 Both under and over 18 years
Evins 2025

Subtotal
Total events:
Test for overall effect: Z = 4.56 (P < 0.00001)

Heterogeneity: Not applicable

3.3.3 Under 18 years

Subtotal
Total events:
Test for overall effect: Not applicable

Heterogeneity: Not applicable

Total (Wald
a
)
Total events:
Test for overall effect: Z = 2.29 (P = 0.02)
Test for subgroup differences: Chi² = 4.40, df = 1 (P = 0.04), I² = 77.3%

Heterogeneity: Tau² (DL
b
) = 0.20; Chi² = 6.87, df = 3 (P = 0.08); I² = 56%

Varenicline
Events
28
8
0
36
45
45
0
81
Total
70
20
1

91
88

88

0

179

Placebo
Events
14
6
1
21
12
12
0
33
Total
70
20
1

91
87

87

0

178
Weight
35.2%
24.9%
5.2%

65.3%
34.7%

34.7%

100.0%

Risk Ratio
M-H, Random, 95% CI
2.00 [1.15 , 3.46]
1.33 [0.57 , 3.14]
0.33 [0.03 , 4.19]

1.64 [0.98 , 2.74]
3.71 [2.11 , 6.51]

3.71 [2.11 , 6.51]

Not estimable

2.04 [1.11 , 3.75]

Risk Ratio
M-H, Random, 95% CI


0.01

0.1

1

10

100


Favours placebo

Favours varenicline


Risk of Bias
A

+

?

+

+
B

+

?

+

+
C

+

+

+

+
D

+

+

+

+
E

+

+

−

+
F

+

+

+

+
G

+

+

+

+

Footnotes

a
CI calculated by Wald-type method.

b
Tau² calculated by DerSimonian and Laird method.
Risk of bias legend

(A) Random sequence generation (selection bias)

(B) Allocation concealment (selection bias)

(C) Blinding of participants and personnel (performance bias)

(D) Blinding of outcome assessment (detection bias)

(E) Incomplete outcome data (attrition bias)

(F) Selective reporting (reporting bias)

(G) Other bias


Analysis 3.4: Number of participants reporting AEs


Study or Subgroup

3.4.1 18 years and over
Caponnetto 2023
Fucito 2024
NCT04602494

Subtotal (Wald
a
)
Total events:
Test for overall effect: Z = 0.95 (P = 0.34)

Heterogeneity: Tau² (DL
b
) = 0.02; Chi² = 2.37, df = 2 (P = 0.30); I² = 16%

3.4.2 Both under and over 18 years
Evins 2025

Subtotal
Total events:
Test for overall effect: Z = 1.26 (P = 0.21)

Heterogeneity: Not applicable

3.4.3 Under 18 years

Subtotal
Total events:
Test for overall effect: Not applicable

Heterogeneity: Not applicable

Total (Wald
a
)
Total events:
Test for overall effect: Z = 1.59 (P = 0.11)
Test for subgroup differences: Chi² = 0.18, df = 1 (P = 0.67), I² = 0%

Heterogeneity: Tau² (DL
b
) = 0.00; Chi² = 2.46, df = 3 (P = 0.48); I² = 0%

Varenicline
Events
36
12
1
49
76
76
0
125
Total
51
18
1

70
88

88

0

158

Placebo
Events
29
5
1
35
68
68
0
103
Total
44
15
1

60
86

86

0

146
Weight
19.0%
2.3%
1.1%

22.4%
77.6%

77.6%

100.0%

Risk Ratio
M-H, Random, 95% CI
1.07 [0.81 , 1.41]
2.00 [0.91 , 4.39]
1.00 [0.32 , 3.10]

1.19 [0.84 , 1.68]
1.09 [0.95 , 1.25]

1.09 [0.95 , 1.25]

Not estimable

1.10 [0.98 , 1.24]

Risk Ratio
M-H, Random, 95% CI


0.1

0.2

0.5

1

2

5

10


Favours varenicline 

Favours placebo


Risk of Bias
A

+

?

+

+
B

+

?

+

+
C

+

+

+

+
D

+

+

+

+
E

+

+

−

+
F

+

+

+

+
G

+

+

+

+

Footnotes

a
CI calculated by Wald-type method.

b
Tau² calculated by DerSimonian and Laird method.
Risk of bias legend

(A) Random sequence generation (selection bias)

(B) Allocation concealment (selection bias)

(C) Blinding of participants and personnel (performance bias)

(D) Blinding of outcome assessment (detection bias)

(E) Incomplete outcome data (attrition bias)

(F) Selective reporting (reporting bias)

(G) Other bias


Analysis 3.5: Weight (lbs) at longest follow-up


Study or Subgroup
Caponnetto 2023

Varenicline
Mean [lb]
169.32
SD [lb]
31.97
Total
51

Placebo
Mean [lb]
172.62
SD [lb]
31.09
Total
44

Mean Difference
IV, Random, 95% CI [lb]
-3.30 [-16.00 , 9.40]

Mean Difference
IV, Random, 95% CI [lb]


-50

-25

0

25

50


Risk of Bias
A

+
B

+
C

+
D

+
E

+
F

+
G

+

Risk of bias legend

(A) Random sequence generation (selection bias)

(B) Allocation concealment (selection bias)

(C) Blinding of participants and personnel (performance bias)

(D) Blinding of outcome assessment (detection bias)

(E) Incomplete outcome data (attrition bias)

(F) Selective reporting (reporting bias)

(G) Other bias


Analysis 3.6: Systolic blood pressure (mmHg) at longest follow-up


Study or Subgroup
Caponnetto 2023

Varenicline
Mean [mmHg]
124.4
SD [mmHg]
8.3
Total
51

Placebo
Mean [mmHg]
126
SD [mmHg]
8.2
Total
44

Mean Difference
IV, Random, 95% CI [mmHg]
-1.60 [-4.93 , 1.73]

Mean Difference
IV, Random, 95% CI [mmHg]


-10

-5

0

5

10


Risk of Bias
A

+
B

+
C

+
D

+
E

+
F

+
G

+

Risk of bias legend

(A) Random sequence generation (selection bias)

(B) Allocation concealment (selection bias)

(C) Blinding of participants and personnel (performance bias)

(D) Blinding of outcome assessment (detection bias)

(E) Incomplete outcome data (attrition bias)

(F) Selective reporting (reporting bias)

(G) Other bias


Analysis 3.7: Diastolic blood pressure (mmHg) at longest follow-up


Study or Subgroup
Caponnetto 2023

Varenicline
Mean [mmHg]
78
SD [mmHg]
8.9
Total
51

Placebo
Mean [mmHg]
77.2
SD [mmHg]
8.4
Total
44

Mean Difference
IV, Random, 95% CI [mmHg]
0.80 [-2.68 , 4.28]

Mean Difference
IV, Random, 95% CI [mmHg]


-10

-5

0

5

10


Risk of Bias
A

+
B

+
C

+
D

+
E

+
F

+
G

+

Risk of bias legend

(A) Random sequence generation (selection bias)

(B) Allocation concealment (selection bias)

(C) Blinding of participants and personnel (performance bias)

(D) Blinding of outcome assessment (detection bias)

(E) Incomplete outcome data (attrition bias)

(F) Selective reporting (reporting bias)

(G) Other bias


Analysis 3.8: Heart rate (bpm) at longest follow-up


Study or Subgroup
Caponnetto 2023

Varenicline
Mean [bpm]
74.5
SD [bpm]
8.9
Total
51

Placebo
Mean [bpm]
76.7
SD [bpm]
12.2
Total
44

Mean Difference
IV, Random, 95% CI [bpm]
-2.20 [-6.55 , 2.15]

Mean Difference
IV, Random, 95% CI [bpm]


-10

-5

0

5

10


Favours varenicline

Favours placebo

Risk of Bias
A

+
B

+
C

+
D

+
E

+
F

+
G

+

Risk of bias legend

(A) Random sequence generation (selection bias)

(B) Allocation concealment (selection bias)

(C) Blinding of participants and personnel (performance bias)

(D) Blinding of outcome assessment (detection bias)

(E) Incomplete outcome data (attrition bias)

(F) Selective reporting (reporting bias)

(G) Other bias


Analysis 4.1: Vaping cessation at 6 months or longer


Study or Subgroup
Sahr 2021

Reduction in nicotine concentration and vape frequency
Events
3
Total
8

No/minimal support
Events
1
Total
9

Risk Ratio
M-H, Random, 95% CI
3.38 [0.43 , 26.30]

Risk Ratio
M-H, Random, 95% CI


0.02

0.1

1

10

50


Favours no/minimal support

Favours vaping reduction

Risk of Bias
A

+
B

+
C

+
D

−
E

+
F

+
G

+

Risk of bias legend

(A) Random sequence generation (selection bias)

(B) Allocation concealment (selection bias)

(C) Blinding of participants and personnel (performance bias)

(D) Blinding of outcome assessment (detection bias)

(E) Incomplete outcome data (attrition bias)

(F) Selective reporting (reporting bias)

(G) Other bias


Analysis 4.2: Vaping cessation at between 3 & 6 months


Study or Subgroup
Sahr 2021

Reduction in nicotine concentration and vape frequency
Events
6
Total
8

No/minimal support
Events
7
Total
9

Risk Ratio
M-H, Random, 95% CI
0.96 [0.57 , 1.64]

Risk Ratio
M-H, Random, 95% CI


0.5

0.7

1

1.5

2


Favours no/minimal support

Favours vaping reduction

Risk of Bias
A

+
B

+
C

+
D

−
E

+
F

+
G

+

Risk of bias legend

(A) Random sequence generation (selection bias)

(B) Allocation concealment (selection bias)

(C) Blinding of participants and personnel (performance bias)

(D) Blinding of outcome assessment (detection bias)

(E) Incomplete outcome data (attrition bias)

(F) Selective reporting (reporting bias)

(G) Other bias


Analysis 4.3: Weight (lbs) at longest follow-up


Study or Subgroup
Sahr 2021

Reduction in nicotine concentration and vape frequency
Mean [lb]
185
SD [lb]
31.73
Total
6

No/minimal support
Mean [lb]
171.88
SD [lb]
38.81
Total
6

Mean Difference
IV, Random, 95% CI [lb]
13.12 [-26.99 , 53.23]

Mean Difference
IV, Random, 95% CI [lb]


-100

-50

0

50

100


Risk of Bias
A

+
B

+
C

+
D

−
E

+
F

+
G

+

Risk of bias legend

(A) Random sequence generation (selection bias)

(B) Allocation concealment (selection bias)

(C) Blinding of participants and personnel (performance bias)

(D) Blinding of outcome assessment (detection bias)

(E) Incomplete outcome data (attrition bias)

(F) Selective reporting (reporting bias)

(G) Other bias


Analysis 4.4: Systolic blood pressure (mmHg) at longest follow-up


Study or Subgroup
Sahr 2021

Reduction in nicotine concentration and vape frequency
Mean [mmHg]
129.33
SD [mmHg]
13.16
Total
6

No/minimal support
Mean [mmHg]
127.88
SD [mmHg]
6.94
Total
9

Mean Difference
IV, Random, 95% CI [mmHg]
1.45 [-10.01 , 12.91]

Mean Difference
IV, Random, 95% CI [mmHg]


-20

-10

0

10

20


Risk of Bias
A

+
B

+
C

+
D

−
E

+
F

+
G

+

Risk of bias legend

(A) Random sequence generation (selection bias)

(B) Allocation concealment (selection bias)

(C) Blinding of participants and personnel (performance bias)

(D) Blinding of outcome assessment (detection bias)

(E) Incomplete outcome data (attrition bias)

(F) Selective reporting (reporting bias)

(G) Other bias


Analysis 4.5: Heart rate (bpm) at longest follow-up


Study or Subgroup
Sahr 2021

Reduction in nicotine concentration and vape frequency
Mean [bpm]
71.83
SD [bpm]
12.83
Total
6

No/minimal support
Mean [bpm]
75.63
SD [bpm]
19.62
Total
9

Mean Difference
IV, Random, 95% CI [bpm]
-3.80 [-20.22 , 12.62]

Mean Difference
IV, Random, 95% CI [bpm]


-20

-10

0

10

20


Favours vaping reduction

Favours no/minimal support

Risk of Bias
A

+
B

+
C

+
D

−
E

+
F

+
G

+

Risk of bias legend

(A) Random sequence generation (selection bias)

(B) Allocation concealment (selection bias)

(C) Blinding of participants and personnel (performance bias)

(D) Blinding of outcome assessment (detection bias)

(E) Incomplete outcome data (attrition bias)

(F) Selective reporting (reporting bias)

(G) Other bias


Analysis 5.1: Vaping cessation at 6 months or longer


Study or Subgroup

5.1.1 18 years and over

Subtotal
Total events:
Test for overall effect: Not applicable

Heterogeneity: Not applicable

5.1.2 Both under and over 18 years
Graham 2021

Subtotal
Total events:
Test for overall effect: Z = 3.37 (P = 0.0007)

Heterogeneity: Not applicable

5.1.3 Under 18 years
Graham 2024

Subtotal
Total events:
Test for overall effect: Z = 4.02 (P < 0.0001)

Heterogeneity: Not applicable

Total (Wald
a
)
Total events:
Test for overall effect: Z = 5.24 (P < 0.00001)
Test for subgroup differences: Chi² = 0.17, df = 1 (P = 0.68), I² = 0%

Heterogeneity: Tau² (DL
b
) = 0.00; Chi² = 0.17, df = 1 (P = 0.68); I² = 0%

Text message-based intervention
Events
0
314
314
287
287
601
Total

0
1304

1304
759

759

2063

No/minimal support
Events
0
239
239
208
208
447
Total

0
1284

1284
744

744

2028
Weight
49.2%

49.2%
50.8%

50.8%

100.0%

Risk Ratio
M-H, Random, 95% CI

Not estimable
1.29 [1.11 , 1.50]

1.29 [1.11 , 1.50]
1.35 [1.17 , 1.57]

1.35 [1.17 , 1.57]

1.32 [1.19 , 1.47]

Risk Ratio
M-H, Random, 95% CI


0.5

0.7

1

1.5

2


Favours no/minimal support

Favours text message-based intervention


Risk of Bias
A

+

+
B

+

+
C


D

+

+
E

+

+
F

+

+
G

+

+

Footnotes

a
CI calculated by Wald-type method.

b
Tau² calculated by DerSimonian and Laird method.
Risk of bias legend

(A) Random sequence generation (selection bias)

(B) Allocation concealment (selection bias)

(C) Blinding of participants and personnel (performance bias)

(D) Blinding of outcome assessment (detection bias)

(E) Incomplete outcome data (attrition bias)

(F) Selective reporting (reporting bias)

(G) Other bias


Analysis 5.2: Combustible tobacco abstinence at 6 months or longer among dual users at baseline


Study or Subgroup
Graham 2021

Text message intervention
Events
197
Total
393

No/minimal support
Events
194
Total
400

Risk Ratio
M-H, Random, 95% CI
1.03 [0.90 , 1.19]

Risk Ratio
M-H, Random, 95% CI


0.02

0.1

1

10

50


Favours no/minimal support

Favours text message intervention

Risk of Bias
A

+
B

+
C


D

+
E

+
F

+
G

+

Risk of bias legend

(A) Random sequence generation (selection bias)

(B) Allocation concealment (selection bias)

(C) Blinding of participants and personnel (performance bias)

(D) Blinding of outcome assessment (detection bias)

(E) Incomplete outcome data (attrition bias)

(F) Selective reporting (reporting bias)

(G) Other bias


Analysis 5.3: Combustible tobacco use uptake at 6 months or longer among exclusive vape users at baseline


Study or Subgroup
Graham 2021

Text message intervention
Events
102
Total
511

No/minimal support
Events
101
Total
525

Risk Ratio
M-H, Random, 95% CI
1.04 [0.81 , 1.33]

Risk Ratio
M-H, Random, 95% CI


0.02

0.1

1

10

50


Favours text message intervention

Favours no/minimal support

Risk of Bias
A

+
B

+
C


D

+
E

+
F

+
G

+

Risk of bias legend

(A) Random sequence generation (selection bias)

(B) Allocation concealment (selection bias)

(C) Blinding of participants and personnel (performance bias)

(D) Blinding of outcome assessment (detection bias)

(E) Incomplete outcome data (attrition bias)

(F) Selective reporting (reporting bias)

(G) Other bias


Analysis 5.4: Number of participants reporting SAEs


Study or Subgroup

5.4.1 18 years and over
Klein 2024

5.4.2 Both under and over 18 years

NCT05140915
a

5.4.3 Under 18 years
Graham 2024

Text message intervention
Events
0
0
0
Total
248
39
759

No/minimal support
Events
0
0
0
Total
260
32
744

Risk Ratio
M-H, Random, 95% CI
Not estimable
Not estimable
Not estimable

Risk Ratio
M-H, Random, 95% CI


0.01

0.1

1

10

100


Favours text message-based 

Favours no/minimal support

Risk of Bias
A

?

?

+
B

?

?

+
C

−


D

+

+

+
E

+

+

+
F

+

+

+
G

+

+

+

Footnotes

a
The intervention included a gamification component; it is not clear whether this was also delivered via text message or via another modality
Risk of bias legend

(A) Random sequence generation (selection bias)

(B) Allocation concealment (selection bias)

(C) Blinding of participants and personnel (performance bias)

(D) Blinding of outcome assessment (detection bias)

(E) Incomplete outcome data (attrition bias)

(F) Selective reporting (reporting bias)

(G) Other bias


Analysis 5.5: Vaping cessation at between 3 & 6 months


Study or Subgroup

5.5.1 18 years and over
Klein 2024

Subtotal
Total events:
Test for overall effect: Z = 0.42 (P = 0.67)

Heterogeneity: Not applicable

5.5.2 Both under and over 18 years

NCT05140915
a

Subtotal
Total events:
Test for overall effect: Z = 0.20 (P = 0.84)

Heterogeneity: Not applicable

5.5.3 Under 18 years

Subtotal
Total events:
Test for overall effect: Not applicable

Heterogeneity: Not applicable

Total (Wald
b
)
Total events:
Test for overall effect: Z = 0.38 (P = 0.71)
Test for subgroup differences: Chi² = 0.08, df = 1 (P = 0.78), I² = 0%

Heterogeneity: Tau² (DL
c
) = 0.00; Chi² = 0.08, df = 1 (P = 0.78); I² = 0%

Text message intervention
Events
98
98
5
5
0
103
Total
248

248
36

36

0

284

No/minimal support
Events
98
98
5
5
0
103
Total
260

260
32

32

0

292
Weight
96.5%

96.5%
3.5%

3.5%

100.0%

Risk Ratio
M-H, Random, 95% CI
1.05 [0.84 , 1.31]

1.05 [0.84 , 1.31]
0.89 [0.28 , 2.79]

0.89 [0.28 , 2.79]

Not estimable

1.04 [0.84 , 1.29]

Risk Ratio
M-H, Random, 95% CI


0.1

0.2

0.5

1

2

5

10


Favours no/minimal support

Favours text message intervention


Risk of Bias
A

?

?
B

?

?
C

−


D

+

+
E

+

+
F

+

+
G

+

+

Footnotes

a
The intervention included a gamification component; it is not clear whether this was also delivered via text message or via another modality

b
CI calculated by Wald-type method.

c
Tau² calculated by DerSimonian and Laird method.
Risk of bias legend

(A) Random sequence generation (selection bias)

(B) Allocation concealment (selection bias)

(C) Blinding of participants and personnel (performance bias)

(D) Blinding of outcome assessment (detection bias)

(E) Incomplete outcome data (attrition bias)

(F) Selective reporting (reporting bias)

(G) Other bias


Analysis 5.6: Number of participants reporting AEs


Study or Subgroup

5.6.1 18 years and over
Klein 2024

5.6.2 Both under and over 18 years

NCT05140915
a

5.6.3 Under 18 years
Graham 2024

Text message intervention
Events
43
0
0
Total
181
39
759

No/minimal support
Events
47
0
0
Total
198
32
744

Risk Ratio
M-H, Random, 95% CI
1.00 [0.70 , 1.44]
Not estimable
Not estimable

Risk Ratio
M-H, Random, 95% CI


0.2

0.5

1

2

5


Favours text message-based intervention

Favours no/minimal support

Risk of Bias
A

?

?

+
B

?

?

+
C

−


D

+

+

+
E

+

+

+
F

+

+

+
G

+

+

+

Footnotes

a
The intervention included a gamification component; it is not clear whether this was also delivered via text message or via another modality
Risk of bias legend

(A) Random sequence generation (selection bias)

(B) Allocation concealment (selection bias)

(C) Blinding of participants and personnel (performance bias)

(D) Blinding of outcome assessment (detection bias)

(E) Incomplete outcome data (attrition bias)

(F) Selective reporting (reporting bias)

(G) Other bias


Analysis 6.1: Vaping cessation at between 3 & 6 months


Study or Subgroup
Michaud 2025

Financial incentives + text messaging
Events
5
Total
10

No/minimal support
Events
5
Total
20

Risk Ratio
M-H, Random, 95% CI
2.00 [0.75 , 5.33]

Risk Ratio
M-H, Random, 95% CI


0.01

0.1

1

10

100


Favours no/minimal support

Favours financial incentives


Analysis 7.1: Vaping cessation at between 3 & 6 months


Study or Subgroup
Michaud 2025

Media literacy e-learning
Events
2
Total
11

No/minimal support
Events
8
Total
19

Risk Ratio
M-H, Random, 95% CI
0.43 [0.11 , 1.68]

Risk Ratio
M-H, Random, 95% CI


0.02

0.1

1

10

50


Favours no/minimal support

Favours media literacy e-learning

Risk of Bias
A

?
B

?
C


D

+
E

+
F

?
G

+

Risk of bias legend

(A) Random sequence generation (selection bias)

(B) Allocation concealment (selection bias)

(C) Blinding of participants and personnel (performance bias)

(D) Blinding of outcome assessment (detection bias)

(E) Incomplete outcome data (attrition bias)

(F) Selective reporting (reporting bias)

(G) Other bias


Analysis 8.1: Participants reporting SAEs


Study or Subgroup
Heffner 2025

App-based + text messaging
Events
0
Total
31

No/minimal support
Events
0
Total
30

Risk Ratio
M-H, Random, 95% CI
Not estimable

Risk Ratio
M-H, Random, 95% CI


0.02

0.1

1

10

50


Favours app + text messaging

Favours no/minimal support

Risk of Bias
A

+
B

+
C


D

+
E

−
F

+
G

+

Risk of bias legend

(A) Random sequence generation (selection bias)

(B) Allocation concealment (selection bias)

(C) Blinding of participants and personnel (performance bias)

(D) Blinding of outcome assessment (detection bias)

(E) Incomplete outcome data (attrition bias)

(F) Selective reporting (reporting bias)

(G) Other bias


Analysis 8.2: Vaping cessation at between 3 & 6 months


Study or Subgroup
Heffner 2025

App-based + text messaging
Events
3
Total
31

No/minimal support
Events
0
Total
30

Risk Ratio
M-H, Random, 95% CI
6.78 [0.37 , 125.95]

Risk Ratio
M-H, Random, 95% CI


0.01

0.1

1

10

100


Favours no/minimal support

Favours app + text messaging


Analysis 8.3: Participants reporting AEs


Study or Subgroup
Heffner 2025

App-based + text messaging
Events
1
Total
31

No/minimal support
Events
3
Total
30

Risk Ratio
M-H, Random, 95% CI
0.32 [0.04 , 2.93]

Risk Ratio
M-H, Random, 95% CI


0.02

0.1

1

10

50


Favours app + text messaging

Favours no/minimal support


Analysis 9.1: Vaping cessation at between 3 & 6 months


Study or Subgroup
Michaud 2025

Media literacy e-learning + financial incentives
Events
3
Total
10

No/minimal support
Events
3
Total
9

Risk Ratio
M-H, Random, 95% CI
0.90 [0.24 , 3.38]

Risk Ratio
M-H, Random, 95% CI


0.02

0.1

1

10

50


Favours no/minimal support

Favours media literacy e-learning + financial incentives


Analysis 10.1: Number of participants reporting SAEs


Study or Subgroup
Palmer 2023

Combination NRT + print-based self-help
Events
0
Total
12

No/minimal support
Events
0
Total
11

Risk Ratio
M-H, Random, 95% CI
Not estimable

Risk Ratio
M-H, Random, 95% CI


0.01

0.1

1

10

100


Favours combination NRT + print-based self-help

Favours no/minimal support

Risk of Bias
A

?
B

?
C

−
D

+
E

+
F

?
G

+

Risk of bias legend

(A) Random sequence generation (selection bias)

(B) Allocation concealment (selection bias)

(C) Blinding of participants and personnel (performance bias)

(D) Blinding of outcome assessment (detection bias)

(E) Incomplete outcome data (attrition bias)

(F) Selective reporting (reporting bias)

(G) Other bias


Analysis 11.1: Numbers of participants reporting SAEs


Study or Subgroup
Klein 2024

Combination NRT + text message-based intervention
Events
0
Total
122

No/minimal support
Events
0
Total
134

Risk Ratio
M-H, Random, 95% CI
Not estimable

Risk Ratio
M-H, Random, 95% CI


0.1

0.2

0.5

1

2

5

10


Favours combination NRT + text messages

Favours no/minimal support

Risk of Bias
A

?
B

?
C

−
D

+
E

+
F

+
G

+

Risk of bias legend

(A) Random sequence generation (selection bias)

(B) Allocation concealment (selection bias)

(C) Blinding of participants and personnel (performance bias)

(D) Blinding of outcome assessment (detection bias)

(E) Incomplete outcome data (attrition bias)

(F) Selective reporting (reporting bias)

(G) Other bias


Analysis 11.2: Vaping cessation at between 3 & 6 months


Study or Subgroup
Klein 2024

Combination NRT + text message-based intervention
Events
53
Total
122

No/minimal support
Events
46
Total
134

Risk Ratio
M-H, Random, 95% CI
1.27 [0.93 , 1.72]

Risk Ratio
M-H, Random, 95% CI


0.5

0.7

1

1.5

2


Favours no/minimal support

Favours combination NRT + text messages

Risk of Bias
A

?
B

?
C

−
D

+
E

+
F

+
G

+

Risk of bias legend

(A) Random sequence generation (selection bias)

(B) Allocation concealment (selection bias)

(C) Blinding of participants and personnel (performance bias)

(D) Blinding of outcome assessment (detection bias)

(E) Incomplete outcome data (attrition bias)

(F) Selective reporting (reporting bias)

(G) Other bias


Analysis 11.3: Number of participants reporting AEs


Study or Subgroup
Klein 2024

Combination NRT + text message-based intervention
Events
33
Total
94

No/minimal support
Events
14
Total
102

Risk Ratio
M-H, Random, 95% CI
2.56 [1.46 , 4.47]

Risk Ratio
M-H, Random, 95% CI


0.1

0.2

0.5

1

2

5

10


Favours combination NRT + text messages

Favours no/minimal support


Analysis 12.1: Participants reporting AEs


Study or Subgroup
Palmer 2025

High dose combination NRT
Events
10
Total
15

Standard dose combination NRT
Events
9
Total
16

Risk Ratio
M-H, Random, 95% CI
1.19 [0.68 , 2.08]

Risk Ratio
M-H, Random, 95% CI


0.02

0.1

1

10

50


Favours high dose

Favours standard dose


Analysis 13.1: Participants reporting AEs


Study or Subgroup
Palmer 2025

Mid-dose combination NRT
Events
13
Total
15

Standard dose combination NRT
Events
9
Total
16

Risk Ratio
M-H, Random, 95% CI
1.54 [0.96 , 2.48]

Risk Ratio
M-H, Random, 95% CI


0.02

0.1

1

10

50


Favours mid-dose

Favours standard dose


Analysis 14.1: Participants reporting AEs


Study or Subgroup
Palmer 2025

High dose combination NRT
Events
10
Total
15

Mid-dose combination NRT
Events
13
Total
15

Risk Ratio
M-H, Random, 95% CI
0.77 [0.51 , 1.16]

Risk Ratio
M-H, Random, 95% CI


0.02

0.1

1

10

50


Favours high dose

Favours mid-dose


Analysis 15.1: Vaping cessation at 6 months or longer


Study or Subgroup
Sahr 2021

Combination NRT
Events
2
Total
7

Reduction in nicotine concentration and vape frequency
Events
3
Total
8

Risk Ratio
M-H, Random, 95% CI
0.76 [0.17 , 3.33]

Risk Ratio
M-H, Random, 95% CI


0.1

0.2

0.5

1

2

5

10


Favours vaping reduction

Favours combination NRT

Risk of Bias
A

+
B

+
C

+
D

−
E

+
F

+
G

+

Risk of bias legend

(A) Random sequence generation (selection bias)

(B) Allocation concealment (selection bias)

(C) Blinding of participants and personnel (performance bias)

(D) Blinding of outcome assessment (detection bias)

(E) Incomplete outcome data (attrition bias)

(F) Selective reporting (reporting bias)

(G) Other bias


Analysis 15.2: Vaping cessation at between 3 & 6 months


Study or Subgroup
Sahr 2021

Combination NRT
Events
3
Total
7

Reduction in nicotine concentration and vape frequency
Events
6
Total
8

Risk Ratio
M-H, Random, 95% CI
0.57 [0.22 , 1.47]

Risk Ratio
M-H, Random, 95% CI


0.1

0.2

0.5

1

2

5

10


Favours vaping reduction

Favours combination NRT

Risk of Bias
A

+
B

+
C

+
D

−
E

+
F

+
G

+

Risk of bias legend

(A) Random sequence generation (selection bias)

(B) Allocation concealment (selection bias)

(C) Blinding of participants and personnel (performance bias)

(D) Blinding of outcome assessment (detection bias)

(E) Incomplete outcome data (attrition bias)

(F) Selective reporting (reporting bias)

(G) Other bias


Analysis 15.3: Weight (lbs) at longest follow-up


Study or Subgroup
Sahr 2021

Combination NRT
Mean [lb]
205.8
SD [lb]
46.86
Total
5

Reduction in nicotine concentration and vape frequency
Mean [lb]
185
SD [lb]
31.73
Total
6

Mean Difference
IV, Random, 95% CI [lb]
20.80 [-27.49 , 69.09]

Mean Difference
IV, Random, 95% CI [lb]


-100

-50

0

50

100


Risk of Bias
A

+
B

+
C

+
D

−
E

+
F

+
G

+

Risk of bias legend

(A) Random sequence generation (selection bias)

(B) Allocation concealment (selection bias)

(C) Blinding of participants and personnel (performance bias)

(D) Blinding of outcome assessment (detection bias)

(E) Incomplete outcome data (attrition bias)

(F) Selective reporting (reporting bias)

(G) Other bias


Analysis 15.4: Systolic blood pressure (mmHg) at longest follow-up


Study or Subgroup
Sahr 2021

Combination NRT
Mean [mmHg]
136.2
SD [mmHg]
13.04
Total
5

Reduction in nicotine concentration and vape frequency
Mean [mmHg]
129.33
SD [mmHg]
13.16
Total
6

Mean Difference
IV, Random, 95% CI [mmHg]
6.87 [-8.67 , 22.41]

Mean Difference
IV, Random, 95% CI [mmHg]


-50

-25

0

25

50


Risk of Bias
A

+
B

+
C

+
D

−
E

+
F

+
G

+

Risk of bias legend

(A) Random sequence generation (selection bias)

(B) Allocation concealment (selection bias)

(C) Blinding of participants and personnel (performance bias)

(D) Blinding of outcome assessment (detection bias)

(E) Incomplete outcome data (attrition bias)

(F) Selective reporting (reporting bias)

(G) Other bias


Analysis 15.5: Heart rate (bpm) at longest follow-up


Study or Subgroup
Sahr 2021

Combination NRT
Mean [bpm]
71.4
SD [bpm]
17.6
Total
5

Reduction in nicotine concentration and vape frequency
Mean [bpm]
71.83
SD [bpm]
12.83
Total
6

Mean Difference
IV, Random, 95% CI [bpm]
-0.43 [-18.96 , 18.10]

Mean Difference
IV, Random, 95% CI [bpm]


-50

-25

0

25

50


Favours combination NRT

Favours vaping reduction

Risk of Bias
A

+
B

+
C

+
D

−
E

+
F

+
G

+

Risk of bias legend

(A) Random sequence generation (selection bias)

(B) Allocation concealment (selection bias)

(C) Blinding of participants and personnel (performance bias)

(D) Blinding of outcome assessment (detection bias)

(E) Incomplete outcome data (attrition bias)

(F) Selective reporting (reporting bias)

(G) Other bias


Analysis 16.1: Numbers of participants reporting SAEs


Study or Subgroup
Klein 2024

Combination NRT
Events
0
Total
126

Text message-based intervention
Events
0
Total
126

Risk Ratio
M-H, Random, 95% CI
Not estimable

Risk Ratio
M-H, Random, 95% CI


0.1

0.2

0.5

1

2

5

10


Favours combination NRT

Favours text message-based intervention

Risk of Bias
A

?
B

?
C

−
D

+
E

+
F

+
G

+

Risk of bias legend

(A) Random sequence generation (selection bias)

(B) Allocation concealment (selection bias)

(C) Blinding of participants and personnel (performance bias)

(D) Blinding of outcome assessment (detection bias)

(E) Incomplete outcome data (attrition bias)

(F) Selective reporting (reporting bias)

(G) Other bias


Analysis 16.2: Vaping cessation at between 3 & 6 months


Study or Subgroup
Klein 2024

Combination NRT
Events
52
Total
126

Text message-based intervention
Events
45
Total
126

Risk Ratio
M-H, Random, 95% CI
1.16 [0.84 , 1.58]

Risk Ratio
M-H, Random, 95% CI


0.5

0.7

1

1.5

2


Favours text message-based intervention

Favours combination NRT

Risk of Bias
A

?
B

?
C

−
D

+
E

+
F

+
G

+

Risk of bias legend

(A) Random sequence generation (selection bias)

(B) Allocation concealment (selection bias)

(C) Blinding of participants and personnel (performance bias)

(D) Blinding of outcome assessment (detection bias)

(E) Incomplete outcome data (attrition bias)

(F) Selective reporting (reporting bias)

(G) Other bias


Analysis 16.3: Number of participants reporting AEs


Study or Subgroup
Klein 2024

Combination NRT
Events
33
Total
96

Text message-based intervention
Events
10
Total
87

Risk Ratio
M-H, Random, 95% CI
2.99 [1.57 , 5.70]

Risk Ratio
M-H, Random, 95% CI


0.1

0.2

0.5

1

2

5

10


Favours combination NRT

Favours text message-based intervention

Risk of Bias
A

?
B

?
C

−
D

+
E

+
F

+
G

+

Risk of bias legend

(A) Random sequence generation (selection bias)

(B) Allocation concealment (selection bias)

(C) Blinding of participants and personnel (performance bias)

(D) Blinding of outcome assessment (detection bias)

(E) Incomplete outcome data (attrition bias)

(F) Selective reporting (reporting bias)

(G) Other bias


Analysis 17.1: Vaping cessation at between 3 & 6 months


Study or Subgroup
Michaud 2025

Media literacy e-learning
Events
2
Total
11

Financial incentives
Events
5
Total
10

Risk Ratio
M-H, Random, 95% CI
0.36 [0.09 , 1.47]

Risk Ratio
M-H, Random, 95% CI


0.02

0.1

1

10

50


Favours financial incentives

Favours media literacy e-learning

Risk of Bias
A

?
B

?
C


D

+
E

+
F

?
G

+

Risk of bias legend

(A) Random sequence generation (selection bias)

(B) Allocation concealment (selection bias)

(C) Blinding of participants and personnel (performance bias)

(D) Blinding of outcome assessment (detection bias)

(E) Incomplete outcome data (attrition bias)

(F) Selective reporting (reporting bias)

(G) Other bias
