# Supplementary material for: Interventions for quitting vaping
Source: Cochrane Database Syst Rev. 2025 Nov 25;2025(11):CD016058. doi: 10.1002/14651858.CD016058.pub3 (PMC12645533; doi:10.1002/14651858.CD016058.pub3)
Supplement: Supplementary file 7 — Supplementary material 7 Justification and methods for 'Living Review' approach [file CD016058-SUP-07-other.html]

Justification and methods for 'Living Review' approach


# Supplementary material 7 to: Interventions for quitting vaping

Butler AR, Lindson N, Livingstone-Banks J, Notley C, Turner T, Rigotti NA, Fanshawe TR, Begh R, Wu AD, Brose L, Conde M, Simonavičius E, Hartmann-Boyce J
  
https://doi.org/10.1002/14651858.CD016058.pub3

The material in this section has been supplied by the author(s) for publication under a Licence for Publication and the author(s) are solely responsible for the material. Cochrane has reviewed this material, but Cochrane has not copyedited, formatted or proofread. Cochrane accordingly gives no representations or warranties of any kind in relation to, and accepts no liability for any reliance on or use of, such material.

Back to top

# Justification and methods for 'Living Review' approach

**Justification for ‘Living Review’ status**

This review will be updated to incorporate eligible new evidence as it becomes available [1]. We are following a living review approach because the evidence currently available is limited and there are high degrees of uncertainty (low- and very low-certainty evidence) for most outcomes, due to the small number of included randomized controlled trials, and the resulting imprecision in effect estimates. This means that some conclusions are likely to change substantially as new evidence emerges. The evidence base is rapidly evolving and our living approach will mean that this review will be able to provide the most up-to-date evidence to decision-makers.

**LSR methodological considerations**

The methods outlined below are specific to maintaining this review of ‘Interventions for vaping cessation’ as a ‘living systematic review’ on the Cochrane Library.

**Search methods for identification of studies**

We will conduct database searches monthly, starting the month that this protocol is published. Search results will be screened on a monthly basis in order to assess whether an update should be triggered (see below: Triggering review updates). We will review our search strategies on an ongoing basis every 12 months, as indexing terms and keywords may change, and new search filters may be published. Such changes will be managed by input from experienced information specialists.

**Contacting authors of ongoing studies**

One review author will contact corresponding authors of potentially relevant ongoing studies as they are identified and ask them to advise when results are available, or to share early or unpublished data. Based on the information and projected time scales shared, we will contact corresponding authors on an ongoing basis to retrieve new evidence as it becomes available.

**Sharing monthly search findings**

We make results of our monthly searches publicly available on our dedicated website, in our two monthly updated briefing documents (plain language and policy makers/researchers) and via social media (X).

**Data synthesis**

We will incorporate the new data into meta-analyses and tables in RevMan Web [2] and supplementary data files (as necessary), and carry out GRADE assessments (GRADEpro GDT) [3]. In-line with Cochrane guidance on carrying out living systematic reviews and the Cochrane Handbook we will not carry out any statistical error adjustments for repeated meta-analyses [1, 4].

**Triggering review updates**

We will conduct a full update of the review (full incorporation and interpretation of all new data within the review and re-publishing) when the accumulating evidence leads to changes in any one of:

- The direction of effect or clinical significance of the findings for one or more outcomes;
- The certainty (e.g. GRADE rating) of one or more outcomes;
- The availability of studies investigating new settings, populations, interventions, comparisons or outcomes.

Formal sequential meta-analysis approaches will not be used for updated meta-analyses, in line with Cochrane guidance for LSRs.

**Future updates of review methods**

The LSR approach acknowledges that reviews may cease to need to be ‘living’ over time, as the review findings become stable, or the question is no longer a priority for decision-makers (Brooker 2019). Eighteen months into this review’s ‘living’ status we will evaluate the LSR approach, including the likely benefits of and challenges to continuing this methodology for this evidence base, and whether such an approach remains warranted. If the evidence is high certainty for all outcomes and all comparisons at that point, meaning further studies are judged very unlikely to impact the effect estimate, we would consider ceasing living mode for this review. If, as is more likely, some or all outcomes are not yet certain, we will facilitate discussions within the author team and Cochrane, as well as engaging with a wider PPI panel and key decision-makers, e.g. policymakers, in order to determine next steps. If the decision is made to continue in living mode, we will review, and if necessary revise, the living review methods described in this Appendix before continuing.

## References

1. Brooker J, Synnot A, McDonald S, Elliott J, Tari Turner T. Guidance for the production and publication of Cochrane living systematic reviews: Cochrane reviews in living mode. https://community.cochrane.org/sites/default/files/uploads/inline-files/Transform/201912\_LSR\_Revised\_Guidance.pdf 2019 (accessed 1 August 2024).
2. Review Manager (RevMan). Version 8.1.1. The Cochrane Collaboration, 2024. Available at https://revman.cochrane.org.
3. GRADEpro GDT. Version accessed 1 November 2023. Hamilton (ON): McMaster University (developed by Evidence Prime), 2023. Available at https://www.gradepro.org.
4. Higgins JP, Thomas J, Chandler J, Cumpston M, Li T, Page MJ, et al, editor(s). *Cochrane Handbook for Systematic Reviews of Interventions* Version 6.4 (updated August 2023). Cochrane, 2023. Available from https://cochrane.org/handbook.
